# Transcription Factor TFAP2C Regulates Major Programs Required for Murine Fetal Germ Cell Maintenance and Haploinsufficiency Predisposes to Teratomas in Male Mice

**DOI:** 10.1371/journal.pone.0071113

**Published:** 2013-08-13

**Authors:** Jana Schemmer, Marcos J. Araúzo-Bravo, Natalie Haas, Sabine Schäfer, Susanne N. Weber, Astrid Becker, Dawid Eckert, Andreas Zimmer, Daniel Nettersheim, Hubert Schorle

**Affiliations:** 1 University of Bonn Medical School, Institute of Pathology, Department of Developmental Pathology, Bonn, Germany; 2 Max Planck Institute for Molecular Biomedicine, Department of Cell and Developmental Biology, Münster, Germany; 3 University of Bonn Medical School, Institute for Reconstructive Neurobiology, Life&Brain Center, Bonn, Germany; University Hospital of Münster, Germany

## Abstract

Maintenance and maturation of primordial germ cells is controlled by complex genetic and epigenetic cascades, and disturbances in this network lead to either infertility or malignant aberration. Transcription factor TFAP2C has been described to be essential for primordial germ cell maintenance and to be upregulated in several human germ cell cancers. Using global gene expression profiling, we identified genes deregulated upon loss of *Tfap2c* in embryonic stem cells and primordial germ cell-like cells. We show that loss of *Tfap2c* affects many aspects of the genetic network regulating germ cell biology, such as downregulation of maturation markers and induction of markers indicative for somatic differentiation, cell cycle, epigenetic remodeling and pluripotency. Chromatin-immunoprecipitation analyses demonstrated binding of TFAP2C to regulatory regions of deregulated genes (*Sfrp1, Dmrt1*, *Nanos3*, *c-Kit*, *Cdk6*, *Cdkn1a*, *Fgf4*, *Klf4*, *Dnmt3b* and *Dnmt3l*) suggesting that these genes are direct transcriptional targets of TFAP2C in primordial germ cells. Since *Tfap2c* deficient primordial germ cell-like cells display cancer related deregulations in epigenetic remodeling, cell cycle and pluripotency control, the *Tfap2c*-knockout allele was bred onto 129S2/Sv genetic background. There, mice heterozygous for *Tfap2c* develop with high incidence germ cell cancer resembling human pediatric germ cell tumors. Precursor lesions can be observed as early as E16.5 in developing testes displaying persisting expression of pluripotency markers. We further demonstrate that mice with a heterozygous deletion of the TFAP2C target gene *Nanos3* are also prone to develop teratomas. These data highlight TFAP2C as a critical and dose-sensitive regulator of germ cell fate.

## Introduction

Germ cell cancers (GCCs) are usually diagnosed between the age of 20–40 years and are the most common cancer type of young men [1]. In infants and pre-pubertal adolescents, teratomas and yolk-sac tumors (Type I GCC) are detected in gonads, cranium or along the body midline. These tumors characteristically consist of tissues of all three germ layers and are generally benign in nature, with rare malignant transformation. It is assumed that the precursor cells of these tumors are primordial germ cells/gonocytes which fail to progress into spermatogonia [2];3;4] and transform into embryonal carcinoma (EC) cells which appear at embryonic day (E) 15.5 [5]. Knowledge of the regulatory network of germ cell specification, maintenance and differentiation is required to further understand the molecular basis of this malignancy and some of the molecular key players have been determined in the past years. Specification of murine primordial germ cells (PGCs) occurs at E6.75 and is mediated by BMP signaling (BMP4/BMP8b) [6]; [7]; [8], which leads to induction of *Prdm1* and *Prdm14.* PRDM1 (BLIMP1) together with PRDM14 are viewed as the key regulators since they orchestrate the re-acquisition of pluripotency and repression of the somatic program in PGCs [9]; [10]; [11]. Single cell analyses in *Blimp1* deficient PGCs suggest that the transcription factor TFAP2C is a downstream target of BLIMP1, as *Tfap2c* level was found to be dramatically reduced in *Blimp1* deficient PGCs [10]. TFAP2C (Tcfap2c/AP-2γ) is a member of the activator protein-2 (AP-2) family, which comprises of five closely related members, namely *Tfap2a-e*. They are characterized by a highly conserved DNA-binding and dimerization motif at the C-terminus. AP-2 transcription factors bind to DNA as functional dimers and their activation is mediated by an N-terminal transactivation domain [12]. *Tfap2c* is expressed in murine PGCs shortly after their specification from E7.25 up to E12.5 after they have migrated and colonized the genital ridges [13]. After arrival in the genital ridges, PGCs initiate further differentiation indicated by downregulation of pluripotency markers *Nanog, Oct3/4, Sox2* [14], exit of mitosis [15], upregulation of germ cell markers *Mutvh* and *Dazl* [16]; [17] and epigenetic remodeling (remethylation, upregulation of *de novo* methylases) [18]. Similar to *Blimp1* [9], *Tfap2c* deficient PGCs are lost shortly after specification at E8.5. In *Tfap2c^−/−^*PGCs, germ cell markers (like *Stella/Dppa3*, *Nanos3*, *Dazl*, *Mutvh*) are downregulated and the somatic gene program is upregulated (*Hoxb1*, *Hoxa1*, *T*) resulting in loss of PGC-fate and somatic differentiation of the cells [13]. In humans, TFAP2C expression is detected in fetal germ cells from week 12 to 37 of pregnancy and in a small subset of cells (gonocytes) in the infantile testes (until 4 months of postnatal age) [19]. Furthermore, high levels of TFAP2C protein were observed in precursor lesions of GCC (carcinoma in situ (CIS)) and classical seminomas [19]; [20].

In this study, we analyzed the consequences of lack of *Tfap2c* in PGC-like cells (PGCLCs). Global cDNA expression profiling revealed that *Tfap2c* deficiency affects cell cycle exit, epigenetic remodeling, germ cell differentiation and regulation of pluripotency. Using chromatin-immunoprecipitation (ChIP) analyses we show that *Sfrp1*, *Dmrt1*, *Nanos3*, *c-Kit*, *Cdk6*, *Cdkn1a*, *Fgf4*, *Klf4, Dnmt3b* and *Dnmt3l* are direct transcriptional target genes of TFAP2C. The data suggest that TFAP2C governs many aspects of PGC development, some of them being also involved in GCC formation. In line with this, we demonstrate that haploinsufficiency of *Tfap2c* or its target gene *Nanos3* lead to high rate of GCC in 129S2/Sv mice, resembling human pediatric germ cell tumors.

## Materials and Methods

### Animals/Ethics Statement

All experiments were conducted according to the German law of animal protection and in agreement with the approval of the local institutional animal care committees (Landesamt für Natur, Umwelt und Verbraucherschutz, North Rhine-Westphalia (approval ID: #8.87-50.10.31.08.238). The experiments were conducted in accordance with the International Guiding Principles for Biomedical Research Involving Animals as announced by the Society for the Study of Reproduction.

### Generation of Blimp1mVenus/Tfap2c^−/−^ ESCs

The derivation of embryonic stem cells (ESCs) from blastocysts was performed by mating Blimp1mVenus (*Tg^(Prdm1-Venus1)Sait^*, MGI:3805969) [21] with *Tfap2c^flox/flox^* (*Tfap2c^tm1Hsc^*, MGI:2176695) [22] mice. ES cells were derived according to published protocols to obtain *Blimp1mVenus/Tfap2c^flox/flox^* ESCs [23]. Transient transfection with a pGK-Cre plasmid resulted in Cre-mediated excision of the floxed sites leading to *Blimp1mVenus/Tfap2c^−/−^*ESCs. Genotyping of *Tfap2c* and *Blimp1mVenus* alleles was done as described previously [21]; [22].

### Embryoid Body Formation

For gene expression analysis *Blimp1mVenus/Tfap2c^ctrl^* and *Blimp1mVenus/Tfap2c^−/−^*ESCs were cultured for embryoid body (EB) formation in DMEM/F12/Neurobasal media (1∶1) supplemented with Glutamax, N2-Supplement (1×), B27-Supplement (1×), L-Glutamine (1×) (all Gibco, Life Technologies, Darmstadt, Germany), BSA (2,5 mg/ml; Sigma Aldrich; Munich, Germany), ß-Mercaptoethanol (100 µM, PAA; GE Healthcare; USA), Insulin (1 mg/ml; Sigma Aldrich; Munich, Germany), PD0325901 (1 µM; Stemgent, San Diego, USA), CHIR99021 (3 µM; Stemgent, San Diego, USA) and Lif (1000 U/ml; Esgro, Merck Millipore, Darmstadt, Germany). Cells were dissociated with Accutase (PAA; GE Healthcare; USA). PGCLC differentiation was performed as described by Hayashi et al. [24]. *Blimp1mVenus/Tfap2c^ctrl^*-PGCLCs and *StellaGFP* ESCs [25] (which were differentiated into EBs for 5 days as described by Young et al. [26]) were used for ChIP analyses. For *StellaGFP*-PGCLC *in vitro* differentiation DMEM supplemented with GlutaMAX, Sodium Pyruvat (Gibco, Life Technologies, Darmstadt, Germany), ß-mercaptoethanol (100 µM; PAA; GE Healthcare; USA), nonessential amino acids (1×; PAA; GE Healthcare; USA); essential amino acids (1×; PAA; GE Healthcare; USA); L-Glutamine (2 mM; PAA; GE Healthcare; USA); Penicillin/Streptomycin (1×; PAA; GE Healthcare; USA); LIF (1000 U/ml; Esgro, Merck Millipore, Darmstadt, Germany); 15% ES FCS (Hyclone; Thermo Scientific; USA) and BMP4 (100 ng; R&D Systems; Wiesbaden, Germany) was used.

### Fluorescence Activated Cell Sorting (FACS)

Embryoid bodies were washed with PBS, dissociated with Accutase (PAA; GE Healthcare; USA) and filtered through a cell strainer (40 µm, BD Biosciences, Heidelberg, Germany). Analysis was performed using FACS Aria III analytical flow cytometer (BD Biosciences, Heidelberg, Germany). All Blimp1mVenus positive cells were recorded per experiment. Data were analyzed using BD FACSDiva 6.3.1 software. Significance was calculated by 2 paired t-test. A p-value of <0.05 was considered to be significant.

### Transfection of TCam-2 Cells

siRNA mediated knockdown of TFAP2C in TCam-2 cells was performed as described by [27]. Scrambled and TFAP2C siRNA were obtained from Origene (TFAP2C SR304789, Origene Technologies, Rockville, USA).

### Chromatin-immunoprecipitation

Aliquots of 1×10^5^ PGCLCs were cross-linked with 1% formaldehyde for 7 min and incubated with 0.1 M glycine for 5 min at room temperature. Cells were resuspended in 200 µl SDS Lysis Buffer and sonicated for 2 cycles (5 min) and 1 cycle (2 min) (30 sec “ON”/30 sec “OFF”). Chromatin-immunoprecipitation (ChIP) experiments were performed as described in [28]. For immunoprecipitation 3 µg antibody against TFAP2C (clone H77/sc-8977 X; SantaCruz, Santa Cruz, USA) and as control antibody against rabbit IgG (Merck Millipore, Darmstadt, Germany) on protein G coupled Dynabeads (Life Technologies, Darmstadt, Germany) were used. Immunoprecipitated DNA was amplified using GenomePlex® Single Cell Whole Genome Amplification Kit (WGA4) (Sigma Aldrich; Munich, Germany) according to manufacturer’s protocol. Amplified ChIP DNA was subjected to qPCR using a Maxima SYBR Green/ROX (Fermentas, Thermo Scientific; USA) in a ViiA™ 7 Real-Time PCR System (Applied Biosystemş Life Technologies, Darmstadt, Germany) according to the manufacturer’s specified parameters. Amplicons were normalized to the non-immunoprecipitated input DNA. Primers used for qPCR are listed in Table S4.

### Semiquantitative RT-PCR

Total RNA from ESCs and sorted PGC-like cells were isolated using RNeasy Mini Kit (Qiagen, Hilden, Germany). First-strand cDNA were synthesized using RevertAid Premium reverse transcriptase (Fermentas, Thermo Scientific; USA). RNA from gonads was extracted using the Nucleo Spin FFPE RNA Kit (Macherey & Nagel, Düren, Germany); 100 ng was used for cDNA synthesis (SuperScriptIII; Invitrogen, Karlsruhe, Germany). RT-PCR-primers are listed in Table S5.

### qRT-PCR

RNA from PGCLCs and gonads was isolated using RNeasy Mini/Micro Kit (Qiagen, Hilden, Germany), respectively. Total RNA from TCam-2 cells was extracted by Trizol (Sigma Aldrich; Munich, Germany) according to manufactureŕs instructions. For amplification of PGCLCs, gonadal and TCam-2 mRNA, SYBR GreenER Reagent Mix (Invitrogen, Karlsbad, Germany)/Maxima SYBR Green/ROX (Fermentas, Thermo Scientific; USA) was used. Housekeeping genes *βActin* and *Gapdh* were used for data normalization. The experiments were performed in biological triplicates. Primer sequences for qRT-PCR are given in Table S6.

### Transcriptome Microarray Analysis

For the microarray experiments GeneChip Mouse Genome 430 2.0 Arrays (Affymetrix, Santa Clara, CA, USA) were used. RNA was extracted from ESCs and sorted PGCLCs using the RNeasy Mini Kit (Qiagen, Hilden, Germany). RNA quality was tested using the Agilent 2100 Bioanalyzer (Agilent Technologies, Böblingen, Germany). For the Affymetrix array, 100 ng of total RNA were used. The arrays were washed and stained according to the manufacturer’s recommendations and scanned in a GeneChip scanner 3000 (Affymetrix, Santa Clara, CA, USA). The normalization was calculated with the RMA (Robust Multi-array Analysis) algorithm [29]. Data post-processing and graphics were performed with in-house developed functions in Matlab. Hierarchical clustering of genes and samples was performed with one minus correlation metric and the unweighted average distance (UPGMA) (also known as group average) linkage method. The gene ontology analysis based on AMIGO gene ontology database [30]. The significance of the gene ontology terms of the differentially expressed genes was analyzed using an enrichment approach based on the hypergeometric distribution. All sets of gene ontology terms were backpropagated from the final term appearing in the gene annotation until the root term of each ontology. The significance (p-value) of the gene ontology terms enrichment was calculated using the hypergeometric distribution. The multitest effect influence was corrected through controlling the false discovery rate using the Benjamini-Hochberg correction at a significance level α = 0.05. The data discussed in this publication have been deposited in NCBI’s Gene Expression Omnibus and are accessible through GEO Series accession number GSE45941.

### Laser Microdissection

Paraffin-embedded tissue was sectioned to 10 µm thickness and mounted on P.A.L.M. Frame slides (Carl-Zeiss Micro Imaging, Göttingen, Germany) and stained with SSEA-1 antibody (R&D Systems, Minneapolis, USA). SSEA-1 positive areas were dissected using P.A.L.M. Microlaser Technology (Carl-Zeiss Micro Imaging, Göttingen, Germany). Dissected tissue was collected in lysis buffer (1 mM EDTA pH 8, 10 mM Tris-HCl pH 8, 0.5% Tween20, 100 mg/ml proteinase K) and incubated at 37°C for 3 hrs, heated to 85°C for 10 min. DNA was extracted with QiaAmp DNA Micro Kit (Qiagen, Hilden, Germany). RNA was extracted with Nucleo Spin FFPE RNA Kit (Macherey & Nagel, Düren, Germany).

### Histology and Immunohistochemistry

Sections and cells were fixed and stained as described by Weber et al. [13]. The following antibodies were used: TFAP2C (1∶300–1∶500, SantaCruz, Santa Cruz, USA), OCT3/4 (1∶100, SantaCruz, Santa Cruz, USA), SSEA1 (1∶100, R&D Systems, Minneapolis, USA), anti-rabbit biotinylated (1∶500; DAKO, Hamburg, Germany), anti-rat biotinylated (1∶200; DAKO, Hamburg, Germany), anti-rabbit Alexa fluor 594 (1∶1000; Life Technologies, Darmstadt, Germany); anti-mouse Alexa fluor 488 (1∶1000; Life Technologies, Darmstadt, Germany). Nuclei were stained using 1∶500 33342 Hoechst (1 mg/ml; Life Technologies, Darmstadt, Germany).

## Results

### *Tfap2c* Deficient ESCs Generate Less PGCLCs

In order to analyze the molecular consequences of *Tfap2c* deficiency in PGCs we took advantage of protocols allowing PGCLC derivation from embryonic stem cells through an epiblast-like cell (EpiLC) intermediate [24]. *Blimp1mVenus* reporter mice [21] were bred into the *Tfap2c^flox/flox^* background [22] and two *Blimp1mVenus/Tfap2c^flox/flox^* embryonic stem cell (ESC) lines were established. Next, the ESC-lines were transiently transfected with a plasmid encoding the Cre-Protein (pgk-Cre) mediating the deletion of the *Tfap2c^flox/flox^* alleles resulting in *Blimp1mVenus/Tfap2c^−/−^* ESCs (#1-*Tfap2c^−/−^* and #2-*Tfap2c^−/−^*) (Fig. S1 A, B, C). For all following experiments, the parental *Blimp1mVenus/Tfap2c^flox/flox^* ESC-lines served as control (ctrl). Under serum and feeder-free conditions ESCs were differentiated for two days into EpiLCs, which display a flattened epithelial-like structure (Fig. S2 A). The expression of marker genes in #1-*ctrl*, #1-*Tfap2c^−/−^*, #2-*ctrl* and #2-*Tfap2c^−/−^* cells was analyzed by semiquantitative RT-PCR to address the question, whether loss of TFAP2C affects differentiation from ESCs to EpiLCs. Upon differentiation into EpiLCs, marker genes for ESCs, *Klf4* and *Prdm14*, were downregulated and epiblast associated genes *Fgf5* and *Dnmt3b* were upregulated (Fig. S2 B). The results suggest that the generation of EpiLCs is not affected by lack of TFAP2C.

Next, PGCLC differentiation was induced by cultivating EpiLCs in medium supplemented with BMP4, BMP8b, SCF, LIF and EGF [24]. Induction of the *Blimp1mVenus* reporter indicated the generation of PGCLCs, which were quantified using fluorescence activated cell sorting (FACS) (Fig. S1 C/S2 C). Using *Tfap2c^−/−^* ESC-lines, we obtained less Blimp1mVenus positive PGCLCs (#1∶9.3% vs. 2.7% (significant); #2∶3% vs. 1.5% (non-significant)) (Fig. S2 D). RT-PCR demonstrated that lack of *Tfap2c* did not affect the expression of the early germ cell marker *Prdm14*, but lead to a reduction of late markers (*Stella, Nanos3*) (Fig. S2 E). Expression of *Prdm14* and the *Blimp1mVenus* reporter indicate that PGCLCs are specified in the absence of TFAP2C. However, the reduction of the number of PGCLCs strongly suggests a role of TFAP2C in the maintenance of the PGC-fate. The fact, that the expression of late germ cells markers is impaired argues against the possibility that the *Tfap2c^−/−^*PGCLCs undergo premature differentiation.

### Transcriptome Analysis of *Tfap2c* Deficient ESCs and *in vitro* Differentiated PGCLCs

**Figure 1:**
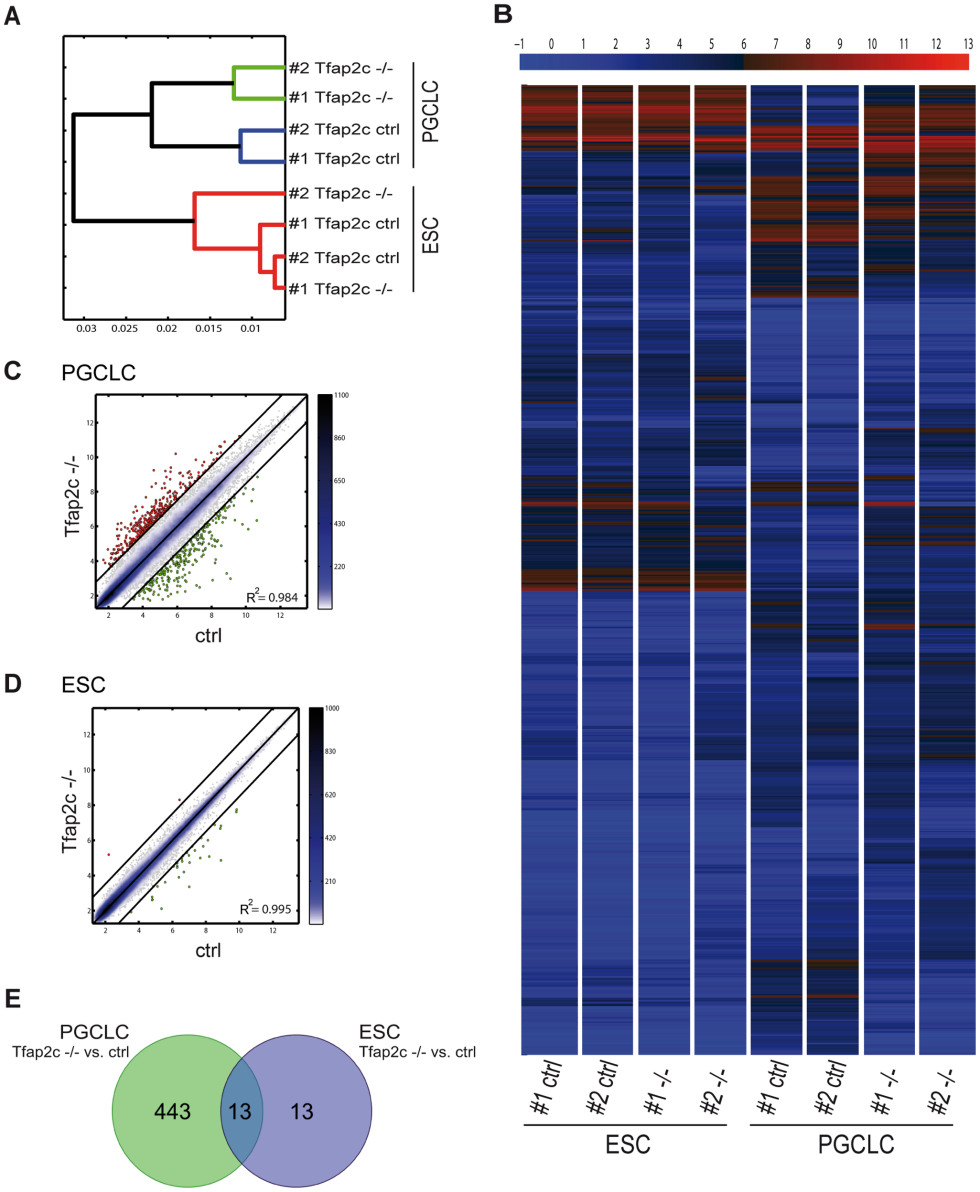
Genes regulated in PGCLCs and ESCs by TFAP2C. Affymetrix microarray gene expression analysis performed with RNA extracted from #1-*ctrl*; #1-*Tfap2c^−/−^* and #2-*ctrl*; #2-*Tfap2c^−/−^* ESCs and PGCLCs. (A) Hierarchical clustering. The red bars cluster the ESCs, green bars the *Tfap2c^−/−^*PGCLCs and blue bars ctrl PGCLCs, respectively. The shorter the horizontal bar that connects two branches the closer are the populations. (B) Heat map was performed with the probes whose range or variation across all samples was at least 3. Color bar on top codifies the gene expression in log2 scale. Red and blue indicate higher and lower relative expression. (C-D) Pairwise scatter plot of global gene expression in ctrl versus *Tfap2c^−/−^*PGCLCs (C) and ESCs (D). Black lines indicate 1.5 fold-change in log2 scale of gene expression levels between paired PGCLCs and ESCs. Color bars on the side display the scattering density with light blue indicating lower and blue higher scatter density. Genes upregulated in *Tfap2c^−/−^* samples are shown as red dots; genes downregulated are shown as green dots. R^2^ = Fisher’s correlation coefficient. (E) Venn diagram; in PGCLCs 455 genes are deregulated; in ESCs 26 genes are deregulated. The intersection part show the commonly deregulated genes (n = 13) by TFAP2C in PGCLCs and ESCs (Fold-change >1.5 in log2 scale).

To gain insight into the consequences of lack of *Tfap2c* on a genome-wide level, gene expression analysis using ESCs and PGCLCs (#1-*ctrl;* #1-*Tfap2c^−/−^*; #2-*ctrl* and #2-*Tfap2c^−/−^*) was performed with Affymetrix Mouse 430 2.0 microarrays. Hierarchical clustering showed that *Tfap2c^−/−^*PGCLCs cluster close together and distant from control samples indicating the high similarity of gene expression profiles within the *Tfap2c^−/−^* and *ctrl* pairs (Fig. 1 A). Heat map shows differentially expressed transcripts between the *Tfap2c^−/−^* and *ctrl* PGCLCs (Fig. 1 B). Scatter plotting of *Tfap2c^−/−^* vs. *ctrl* PGCLCs identified 455 significantly deregulated genes (Fold-change >1.5 in log2 scale) (Fig. 1 C; Table S1). Of note, TFAP2C has only moderate effects in ESCs (albeit *Tfap2c* is expressed in 10–15% of ESCs) [23] as indicated by hierarchical clustering, heat mapping and scatter plotting (Fig. 1 A, B, D). In ESCs, only 26 genes were deregulated after loss of TFAP2C (Table S2). Comparing the PGCLC with ESC datasets revealed an overlap of 13 genes (Fig. 1 E; Table S3). These results indicate that TFAP2C controls a diverse set of genes in ESCs and PGCLCs and that the lack of this protein in PGCLCs affects the expression of far more genes compared to ESCs.

Next we performed gene ontology analysis [30] (Data S1) and categorized the genes deregulated in *Tfap2c^−/−^*PGCLCs in relation to their biological function. Here, it became evident that in *Tfap2c^−/−^*PGCLCs many genes associated with somatic differentiation (category I) are upregulated, whereas genes associated with germ cell maintenance and maturation (category II) are downregulated (Table 1). Of note, we also included few genes in Table 1 with relevance to the respective category displaying a fold-change rate below our initial cutoff (≤1.5 in log2 scale, see Table 1). These data suggest an impairment of PGC to gonocyte progression and derepression of somatic differentiation upon loss of TFAP2C. These results confirm earlier data, where markers indicative for somatic differentiation were induced in *Tfap2c* deficient EBs [13]. In addition, we found upregulation of genes associated with cell cycle regulation (category III), pluripotency (category IV) and epigenetic modification (category V) in *Tfap2c^−/−^*PGCLCs. The data indicate that in migrating PGCs, TFAP2C represses the cell cycle, pluripotency program and DNA methylation.

**Table 1:** Categorized deregulated genes in *Tfap2^−/−^*PGCLCs based on gene ontology analysis.

Category	Upregulatedgenes	FC	Function	Downregulated genes	FC	Function
**I:**	**Bcl11a**	1.93	leukemogenesis and hematopoiesis	**Gata2**	−1.87	hematopoiesis
**Somatic**	**Cited2**	1.87	CNS development	**Lhx1**	−1.59	mesodem formation
**differentiation**	**Chd7**	1.67	CNS development	**Slit2**	−2.34	neurogenesis
	**Cobl**	2.43	neural tube closure	**Kctd15**	−3.20	inhibit neural crest formation
	**Ehna**	2.01	neural tube closure	**Pecam1/CD31**	−1.50	mesoderm development
	**Epha2**	1.53	neural tube development			
	**Gbx2**	2.36	gastrulation-brain specific			
	**Gli2**	3.48	neural tube development			
	**Hck**	2.83	hemopoietic cell kinase			
	**Hhex**	1.52	hematopoietically expressed homeobox gene			
	**Hoxa5**	1.97	mammary gland- lung development			
	Hoxa1	1.13	central nervous system neuron differentiation			
	Hoxa3	1.13	cartilage- and thymus development			
	Hoxb1	1.03	CNS development			
	**Mtap1b**	1.70	CNS development			
	**Ncam1**	2.42	neuron-neuron adhesion			
	**Nefl**	3.56	neurogenesis			
	**Neurod1**	1.57	neurogenesis			
	**Pou4f2**	1.58	expressed in brain			
	**Pten**	1.63	CNS development			
	**Robo1**	1.79	neurogenesis			
	**Sfrp1**	3.12	neural tube closure and development			
	**Sfrp2**	1.66	neural tube development			
	**Smarca1**	2.06	neuron differentiation, brain development			
	**Tcf7l2**	2.56	neural tube development			
	**Zic2**	3.71	CNS development; neural tube closure			
	**Zic5**	3.14	neural tube closure			
**II:**	**Cited2**	1.87	sex determination	**Aurkc**	−2.06	key regulator mitosis; meiosis
**Germ cell**	**Pten**	1.63	tumor suppressor; apoptosis	**Bik**	−2.20	spermatogenesis, apoptosis
**maintenance**	**Sprink3**	2.54	inhibits calcium uptake in spermatozoa	Cdk16	−1.00	required for spermatogenesis
**and**				**Ceacam10**	−2.98	sperm surface protein
**maturation**				**Chdh**	−4.80	localized in mitochondrial
				**Cxcr4**	−1.94	germ cell migration
				Dazl	−1.40	gametogenesis; spermatogenesis
				**Dmrt1**	−2.68	male sex determination and differentiation
				**Hk1**	−3.04	expressed in meiosis and postmeiotic germ cells
				**Immp2l**	−1.57	spermatogenesis
				**Kit**	−1.91	cell survival, migration and proliferation
				**Phf13/Spoc1**	−1.85	spermatogenesis
				**Rhox5**	−4.29	spermatogenesis, sperm maturation
				**Rhox4a**	−2.70	in fetal germ cells expressed
				**Rhox6**	−6.11	germ cell lineage determination
				**Rhox9/Psx2**	−5.59	oogenesis
				**Sept4**	−1.63	sperm capacitation; mitochondrion organization
				**Slco4c1**	−2.32	sperm maturation
				**Spa17**	−2.30	sperm surface protein
				**Stella/Dppa3**	−4.27	PGC specific protein
				**Stk31**	−2.40	male germ cell specific expression
				**Tes**	−2.04	cell adhesion, cell proliferation
**III:**	**Ccnd1**	2.33	interacts with Cdk4/Cdk6	**Urgcp**	−3.17	regulation of Ccnd1
**Cell cycle**	**Ccnt2**	1.88	interacts with Cdk9			
	**Cdc14a**	1.57	cell cycle; cell division			
	**Cdk6**	1.50	promotes G1/S transition			
	Cdk7	1.17	cell cycle; cell division			
	Cdkn1a/p21	1.02	bind and inhibit cyclin-dependent kinase			
	**Plk2**	1.68	involved in G1/S phase transition			
	**S100a10**	2.15	cell cycle progression and differentiation			
**IV:**	**Eras**	3.52	maintenance of ESC pluripotency			
**Pluripotency**	**Fgf4**	2.94	regulation cell proliferation, differentiation			
	FoxD3	1.03	early direct response gene of Oct3/4			
	**Gbx2**	2.36	cell pluripotency and differentiation			
	**Jam2**	2.47	early direct response gene of Oct3/4			
	**Klf4**	2.22	maintenance of ESC pluripotency			
	**Msi2**	2.31	RNA binding protein; self-renewal of ESC			
	**Nr0b1**	1.62	maintenance of ESC pluripotency			
	Tdgf1	1.36	early direct response gene of Oct3/4			
**V:**	Dnmt3b	1.34	genome-wide de novo methylation	**Pcgf5**	−2.40	chromatin remodeling; histone modification
**Epigenetics**	**Dnmt3l**	3.21	DNA methyltransferase like enzyme			
	Smarca6/Hells	1.09	de novo or maintenance of DNA methylation			
	**Mbd2**	1.54	recruits Hdacs and Dnmts; gene silencing			
	Tet2	1.27	conversion of 5mC to 5hmC			
	Uhrf1	1.45	major role in the G1/S transition			

bold: genes with fold-change >1.5 in log2 scale; regular: genes with fold-change ≤1.5 in log2 scale.

**Figure 2:**
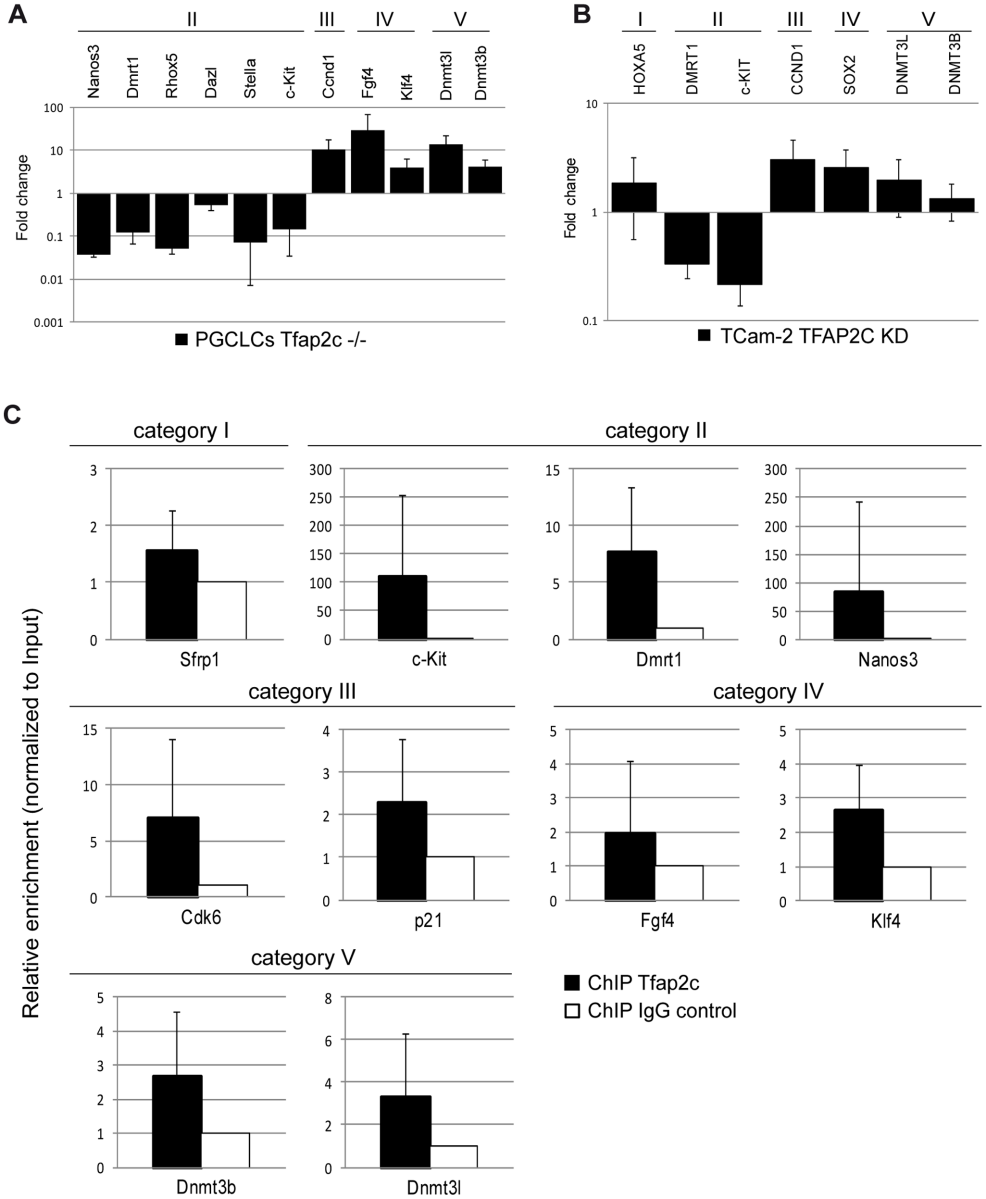
Validation of cDNA microarray data. (A) Quantitative RT-PCR of a subset of markers with RNA isolated from ctrl and *Tfap2c^−/−^*PGCLCs. Expression levels were normalized to *βActin* and expression level of *ctrl* PGCLCs were set to 1. qRT-PCR was performed in biological triplicates. Error bars indicate standard deviation. (B) Quantitative RT-PCR of a subset of markers with RNA isolated from TCam-2 cells after siRNA mediated knockdown of TFAP2C. Expression levels were normalized to GAPDH and expression levels of scrambled-siRNA-transfection were set to 1. qRT-PCR was performed in biological triplicate. Error bars indicate standard deviation. (C) ChIP/qPCR analysis for Tfap2c was performed with four biological replicates of PGCLCs. The qPCR results were calculated with the percentage input method and ChIP analyses with IgG antibody served as control and were set to 1. Error bars indicate standard deviation. ChIP analysis demonstrates increased binding of TFAP2C at indicated loci. (A) – (C) Marker genes were grouped in categories: somatic differentiation (category I), germ cell maintenance and maturation (category II), cell cycle regulation (category III), pluripotency (category IV) and epigenetic modification (category V).

Subsequently, differential expression seen in the cDNA-microarray of selected marker genes was validated using qRT-PCR in PGCLCs (Fig. 2 A) (downregulated: *Nanos3* (−4.78), *Rhox5* (−4.26), *Stella* (−3.84), *Dmrt1* (−3.07), *c-Kit* (−2.78) and *Dazl* (−0.88) and upregulated: *Fgf4* (4.84), *Dnmt3l* (3.79), *Ccnd1* (3.36), *Dnmt3b* (2.07) *and Klf4* (1.95) [fold-changes in log2 scale]). Furthermore, expression of several markers was determined after siRNA mediated knockdown of TFAP2C in the human seminoma-like cell line TCam-2 [31] (Fig. 2 B). Here, after reduction of TFAP2C (to ∼30% compared to the control level) *c-KIT* (−2.22) and *DMRT1* (−1.58) were downregulated and *CCND1* (1.60), *SOX2* (1.34), *DNMT3L* (0.98), *HOXA5* (0.88), and *DNMT3B* (0.39) were upregulated (all fold-changes in log2 scale). So, in both systems all selected genes were deregulated in agreement with the array analysis. This suggests a conserved function of TFAP2C/Tfap2c in humans and mice.

### ChIP Analysis Reveals Direct Transcriptional Targets

To evaluate direct binding of TFAP2C to promotor regions of deregulated genes we performed TFAP2C specific ChIP-analysis using PGCLCs derived from *Stella-GFP* ESCs [25] and *Blimp1mVenus/Tfap2c^ctrl^* ESCs. Putative TFAP2C binding sites identified by the rVista algorithm (based on TRANSFAC) [32] (sequence information Table S4) in genomic regions of *Sfrp1, Dmrt1*, *Nanos3*, *c-Kit*, *Cdk6*, *Cdkn1a*, *Fgf4*, *Klf4*, *Dnmt3b* and *Dnmt3l* were enriched for TFAP2C protein (vs. IgG control) (Fig. 2 C), implicating those genes as direct target genes of TFAP2C.

### Male Mice Heterozygous for the *Tfap2c* Deletion Develop Teratomas

**Figure 3:**
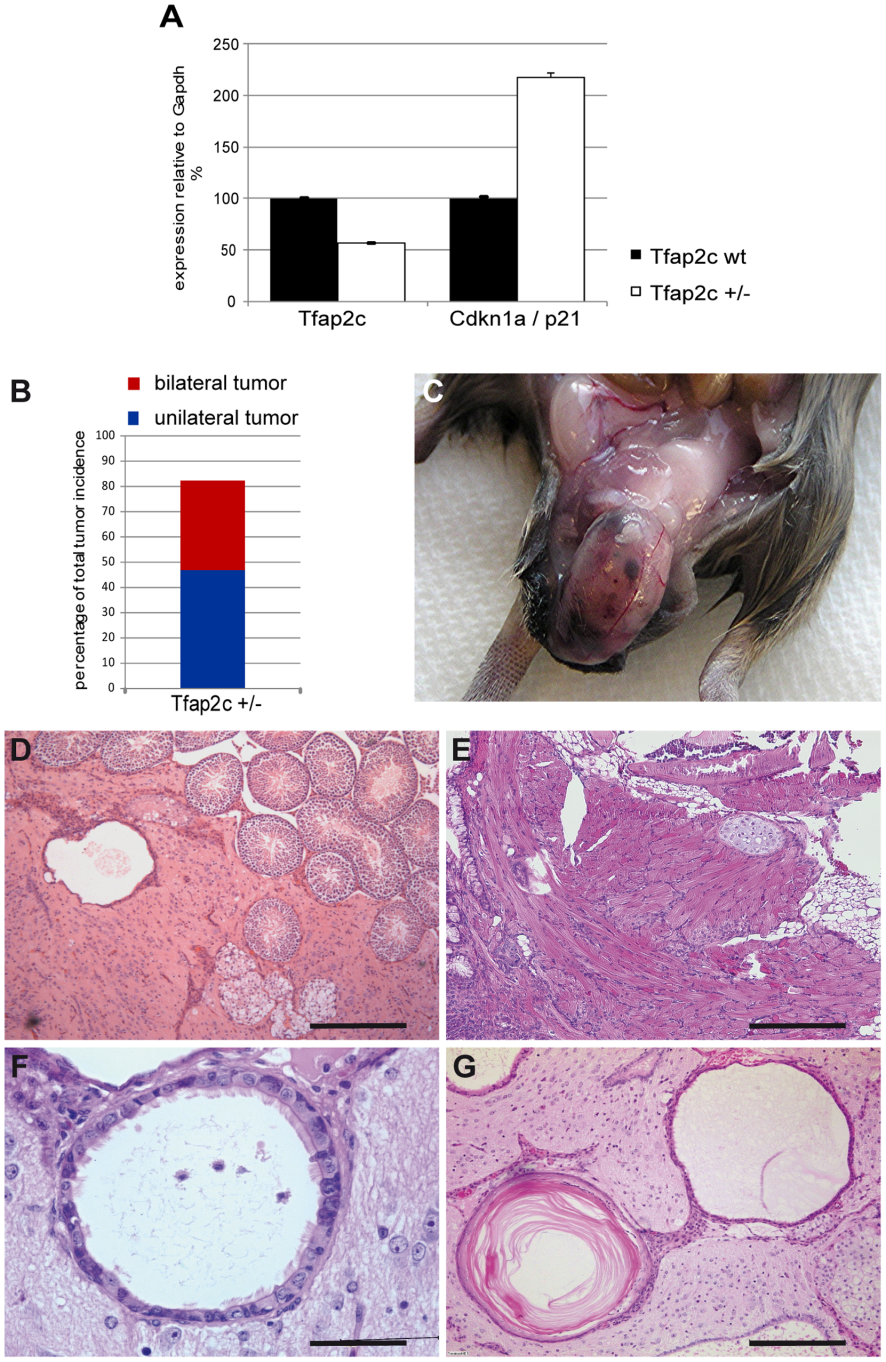
Teratoma development in *Tfap2c* heterozygous mice in 129S2/Sv genetic background. (A) Quantitative RT-PCR with RNA isolated from E12.5 genital ridges of wt and *Tfap2c^+/−^* embryos was performed. Expression levels of *Tfap2c* and *p21* were normalized to *Gapdh*. qRT-PCR was performed in biological duplicates. Error bars indicate standard deviation. (B) Percentage of total tumor incidence in *Tfap2c* heterozygous 129S2/Sv male mice. The seventh generation in 129S2/Sv shows 82% (n = 51) testicular tumors. Red bar: bilateral cases (35%), blue bar: unilateral tumors (47%). (C) Gross pathology of testicular teratoma in *Tfap2c^+/−^* male mice. (D–G) HE-staining of testicular teratomas of 3–6 month old mice. Tumors show immature glia (D), mature cartilage, muscle (E), respiratory epithelium (F) and squamous epithelium (G). Scale bars: 200 µm.

Our analyses revealed, that TFAP2C is not only involved in suppression of the somatic program but also controls cell cycle, epigenetic modifications and pluripotency. Since these pathways are discussed to be deregulated in GCC, we wondered whether reduction of *Tfap2c* could contribute to the initiation of these tumors. The *Tfap2c^tm1Hsc^* (MGI: 93391) allele [22] was bred into the 129S2/Sv genetic background and screened for effects of haploinsufficiency. To determine whether loss of one allele of *Tfap2c* affected the overall levels of *Tfap2c*-mRNA, E12.5 genital ridges of control and *Tfap2c^+/−^* embryos were dissected, RNA isolated and qRT-PCR analysis performed. Deletion of one *Tfap2c*-allele leads to reduction of *Tfap2c*-mRNA (Fig. 3 A) and to upregulation of the direct target *Cdkn1a*/*p21* (Fig. 3 A). Starting from the seventh generation in 129S2/Sv 82% (n = 51) of all heterozygous males developed testicular tumors (Fig. 3 B) (35% presented as bilateral cases), whereas wildtype littermates (n = 22) remained tumor-free. Tumors presented as teratomas (Fig. 3 C) and displayed a variety of tissues like immature glia (Fig. 3 D) and mature cartilage (Fig. 3 E), muscle (Fig. 3 E), respiratory epithelium (Fig. 3 F) as well as squamous epithelium (Fig. 3 G). Of note, the tumors did not have a negative effect on life expectancy of affected animals, as selected mice were observed for over 1.5 years without apparent impairment of their general well-being.

### Pluripotency is Retained in Foci of *Tfap2c* Heterozygous Testes

**Figure 4:**
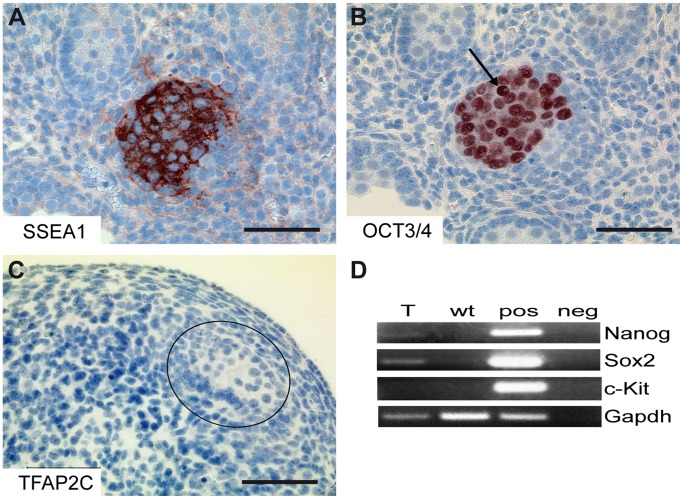
Pluripotency maintaining and differentiation block show EC like nature of lesion. (A–C) IHC staining of E16.5 embryonal testis. (A) SSEA1, (B) OCT3/4 and (C) TFAP2C. Scale bars: 50 µm. (B) Black arrow show cells with large, highly condensated nuclei as well as clearly visible nucleoli. (D) RT-PCR from RNA of microdissected SSEA-1 positive foci of E16.5 testes detecting *Nanog*, *Sox2* and *c-Kit*. Comparison of microdissected SSEA-1 positive tissue (T) and SSEA-1 negative tissue (wt). *Gapdh* served as control.

Tumors were reminiscent of early childhood germ cell cancer (Type I) in humans. These tumors initiate early during germ cell development and are characterized by failure of PGCs to downregulate pluripotency markers. The lesions represent hallmarks of embryonal carcinomas [1]. Here, testes of E15.5 and E16.5 *Tfap2c^+/−^* embryos were dissected, paraffin embedded and sectioned. Immunohistochemical staining of *Tfap2c^+/−^* gonads revealed foci of cells which maintain strong SSEA1 (Fig. 4 A) and OCT3/4 (Fig. 4 B) protein levels. The cells of these foci were still localized within the confines of the seminiferous tubule at this time point. Serial sections suggest that OCT3/4 positive foci were negative for TFAP2C (Fig. 4 C). SSEA-1 positive foci of E16.5 testes were micro-dissected and subjected to RT-PCR and adjacent (SSEA-1 negative) testes tissue served as control. In SSEA1-positive foci *Nanog* and *Sox2* mRNA was detectable (Fig. 4 D). The fact, that pluripotency genes are expressed and cells within the foci possess large, highly condensated nuclei as well as clearly visible nucleoli (Fig. 4 B) indicate that these cells resemble embryonal carcinoma (EC) cells [33]. The absence of *c-Kit* expression further corroborates the EC-like nature of this lesion (Fig. 4 D).

### Teratoma Development in Male Mice Heterozygous for *Nanos3*

**Figure 5:**
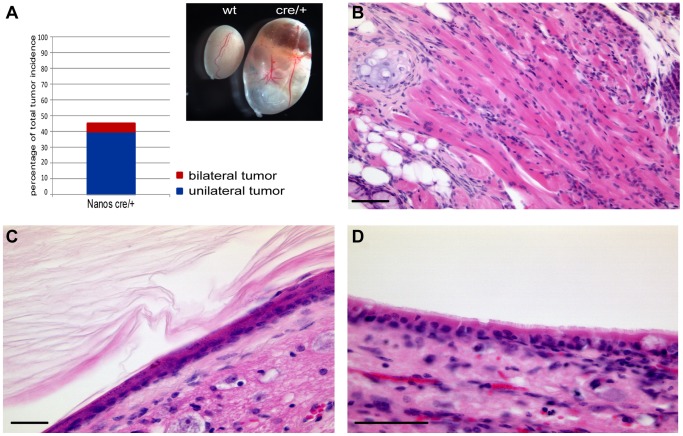
Teratoma development in *Nanos3* heterozygous mice in 129S2/Sv genetic background. (A) Percentage of total tumor incidence in *Nanos3* heterozygous 129S2/Sv male mice. 45% (n = 20) showed testicular tumors. Red bar: bilateral cases (5%); blue bar: unilateral cases (40%). Testes of control (wt) and *Nanos3* heterozygous (cre/+) mice (inset in A). (B–D) HE-staining of testicular teratomas of 4–8 month old mice. Tumors show tissues of all three germ layers: muscle (B), squamous epithelium (C) and respiratory epithelium (D). Scale bars: 50 µm.

Since we identified *Nanos3* as a direct transcriptional target of TFAP2C we addressed the question, whether *Nanos3* heterozygous mice also develop tumors. Therefore, we bred the *Nanos3*-Cre knock-in allele (*Nanos3^tm2.1(cre)Ysa^*, MGI:4358405), which represents a null allele, [34] into the 129S2/Sv genetic background. Interestingly, from the fourth generation in 129S2/Sv 45% of *Nanos3* heterozygous males showed teratoma formation (n = 20), with 5% showing bilateral tumors (Fig. 5 A), whereas control animals (n = 17) remained tumor-free. The histology of teratomas found in *Nanos3* heterozygous animals is comparable to those of *Tfap2c* heterozygous mice (Fig. 5 B mesoderm; Fig. 5 C ectoderm; Fig. 5 D endoderm). These data suggest that teratoma susceptibility mediated by *Tfap2c* in 129S2/Sv genetic background is at least in part due to reduction of *Nanos3*. However, tumor incidence in *Nanos3* heterozygous mice was lower and tumor progression was slower compared to *Tfap2c* heterozygous animals, arguing for additional targets of TFAP2C involved in GCC pathogenesis.

## Discussion

In this study, we analyzed the network of genes regulated by TFAP2C during germ cell development. Using an *in vitro* PGC differentiation protocol we observed deregulation of genes controlling cell cycle, somatic differentiation, epigenetic modification, pluripotency and germ cell maintenance and maturation upon loss of *Tfap2c*. ChIP analysis indicates direct binding of TFAP2C to *Sfrp1, Dmrt1*, *Nanos3*, *c-Kit*, *Cdk6*, *Cdkn1a*, *Fgf4*, *Klf4*, *Dnmt3b* and *Dnmt3l*. Since some of the genes deregulated upon loss of *Tfap2c* are known to induce GCC in mice, we bred the *Tfap2c* mutation onto the 129S2/Sv genetic background. We demonstrate that reduction of TFAP2C protein level leads to persisting expression of pluripotency and cell cycle markers in foci representing precursor lesions. Consequently, incidence of GCC increases up to 85% in *Tfap2c^+/−^* males. Tumor susceptibility seems at least in part to be mediated by the TFAP2C target gene *Nanos3* since *Nanos3* heterozygous mice also develop GCC.

The microarray analysis identified a set of 455 deregulated genes in *Tfap2c^−/−^*PGCLCs compared to the control. Interestingly, the deregulated genes in *Tfap2c^−/−^*PGCLCs could be categorized in five groups representing diverse biological functions; somatic differentiation (category I), germ cell maintenance and maturation (category II), cell cycle (category III), pluripotency (category IV) and epigenetics (category V). In addition, ChIP analyses demonstrated that TFAP2C binds to regulatory elements of genes of all these categories. Thus, aside from the role as inhibitor of somatic differentiation [13], [35], TFAP2C seems to orchestrate all aspects of germ cell development instead of controlling only one category which, after deregulation would affect all others as a secondary effect.

Genes related to neural tube development/closure and central nervous system development are the GO terms significantly enriched in *Tfap2c^−/−^*PGCLCs. Interestingly, we observed that tumors in *Tfap2c^+/−^* and *Nanos3^+/−^* testes predominantly consist of undifferentiated neuronal tissue (glia cells). In general, Stevens described that teratomas induced by grafting genital ridges consist predominantly of neural tissue [36]. This suggests that TFAP2C specifically inhibits neural tube development/closure and central nervous system development during germ cell development. Here, the direct transcriptional target *Sfrp1* (Secreted frizzled-related protein 1) might be involved. It acts by antagonizing BMP-signaling in the caudal neural tube, resulting in neural tube closure defects [37]. This further strengthens the notion, that loss of TFAP2C leads to derepression of somatic programs, specially neuronal cell fate, and loss of germ cells in *Tfap2c* knockout mice [13].

In addition, loss of TFAP2C severely impairs the expression of genes involved in germ cell maintenance and maturation such as *Stella*, *Dazl*, *Immp2l*, *Rhox4a*, *Rhox5*, *Rhox6*, *Rhox9*, *Dmrt1*, *c-Kit*, *Cxcr4* and *Nanos3*. Among them, *Dmrt1*, *c-Kit* and *Nanos3* are direct target genes as determined by ChIP analyses. These data indicate that the lack of germ cell maturation markers is not simply a consequence of the derepressed somatic program. TFAP2C directly orchestrates the expression of the genes relevant for maintenance and maturation of PGCs/gonocytes up to E12.5, the last day expression of *Tfap2c* can be detected [13].

Lack of TFAP2C led to upregulation of cell cycle and pluripotency markers as well as epigenetic regulators. This indicates a role of TFAP2C to retain the capacity of germ cells to repress pluripotency, controlling the cell cycle and epigenetic reprogramming in the germ line. Here, *Cdk6*, *Cdkn1a/p21*, *Fgf4*, *Klf4*, *Dnmt3b* and *Dnmt3l* could be shown to be direct TFAP2C targets, demonstrating that TFAP2C acts as a repressor for these genes. Upon loss of TFAP2C, cell cycle control genes such as *Ccnd1* and *Cdk6* are upregulated. *Ccnd1* (*Cyclin D1*) is expressed in proliferating gonocytes and spermatogonia, suggesting a role for *Ccnd1* in spermatogonial proliferation [38]. *Ccnd1* expression is downregulated at E16.5. Interestingly, the percentage of germ cells, which continue to express *Ccnd1* correlates with the risk of the respective mouse strain to develop teratoma [39]. It is known, that upregulation of *Ccnd1* and its partner molecule *Cdk6* lead to phosphorylation and inactivation of the retinoblastoma gene product (pRB), inducing expression of genes necessary for G1 to S-phase transit [40]. Furthermore, ectopic expression of *Ccnd1* is sufficient to promote tumor formation by mediating growth factor independence and forces quiescent cells to reenter the cell cycle [40], [41]. Of note, a rapid progression from the G1 to S phase of the cell cycle facilitates the maintenance of pluripotency [42], [43]. Hence, we believe that *Tfap2c^−/−^* germ cells might fail to initiate the mitotic arrest and as a consequence do not differentiate further into gonocytes. Interestingly, the direct transcriptional target *Cdkn1a/p21* is upregulated in *Tfap2c^−/−^*PGCLCs and heterozygous E12.5 genital ridges. Direct binding to and repression of *p21* has been described before using MCF-7 breast cancer cells [44]. While upregulation of the cell cycle repressor *p21* seems counterintuitive with regard to the above reported upregulation of *Ccnd1/Cdk6*, it has to be noted that p21 is not only associated with cell cycle arrest [45], [46], [47] but also described as a regulator of p53-dependent and p53-independent apoptosis [48], [49]. Using *Dead End* ^−/−^ (*Dnd1^−/−^*) mice, testicular tumors were observed after repression of Bax-mediated apoptosis [50]. Here, we speculate that *Tfap2c^+/−^* germ cells are less prone to apoptosis due to elevated level of *p21*.

Loss of TFAP2C caused upregulation of the pluripotency associated genes *Eras*, *Fgf4*, *Klf4* and *Nr0b1*. *Fgf4* and *Klf4* are direct transcriptional targets. In addition, it has been reported, that loss of the TFAP2C target gene *Dmrt1* leads to upregulation of pluripotency markers *Sox2*, *Nanog*, *Oct3/4* and *E-cadherin* in testes of E13.5 mice. *Sox2* is a direct transcriptional target of DMRT1 [51]. In addition, we found three OCT3/4 dependent genes [52] *Jam2*, *FoxD3* and *Tdgf1* upregulated in *Tfap2c^−/−^*PGCLCs. Interestingly, expression of *Eras* (*ES-expressing ras*) a gene related to teratoma formation in ESCs [53] was also upregulated. Again, such an upregulation is also found upon deletion of *Dmrt1* [51], indicating that *Dmrt1* as a downstream target of TFAP2C cooperates in balancing pluripotency and germ cell differentiation. We conclude that TFAP2C represses the transcription of pluripotency-associated genes in PGCs.

During germ cell migration loss of global DNA-methylation is observed [54]. Loss of DNA methylation in migrating PGCs occurs from approximately E8.0– E11.5 [55], [56]. TFAP2C seems to contribute to this process by direct binding to regulatory regions and repression of the methyltransferases *Dnmt3b*, *Dnmt3l*, and *ubiquitin-like, containing PHD and RING finger domain 1* (*Uhrf1*). These genes are essential components of the *de novo* methylation machinery [11], [57], [58], [59]. After arrival at the genital ridges (E12.5), when *Tfap2c* is downregulated, the methylation levels are reinstalled [60]. Interestingly, embryonal carcinomas display high levels of DNMT3B and DNMT3L [61], [62], [63], [64]. Hence, the failure to repress the *de novo* methylation indicates, that these cells are prone to transform to EC-like cells. There is an association between DNA methylation and GCC susceptibility due to the fact that the global loss of DNA methylation process overlaps with the critical period for GCC formation [65].

We demonstrated here that *Dmrt1* is a direct target gene of TFAP2C in PGCLCs. Interestingly, mice deficient for *Dmrt1* are sterile and 90% develop GCC [51]. A recent study by Krentz et al. investigated teratoma susceptibility in *Dmrt1^fl/fl^ : Nanos3^Cre/+^* male mice [66]. Interestingly, the authors found *Nanos3* haploinsufficiency to modify the susceptibility of *Dmrt1* deficient male mice by elevating the tumor incidence, clearly demonstrating a role of *Nanos3* in germ cell tumor development. However, in contrast to our results, the study of Krentz et al. could not demonstrate increased teratoma formation by *Nanos3* heterozygosity on its own. As discussed there, this fact could be due to insufficient outbreeding to the 129Sv genetic background. Further, 129S6/Sv : 129S1/Sv mice were used instead of 129S2/Sv as used in our study. Strain specific variation in tumor susceptibility may have an influence on the experimental outcome.

Mice deficient for *Pten* and *Dnd1* also display increased teratoma formation [67], [68], [69]. Since, we did not see downregulation of these genes in *Tfap2c^−/−^*PGCLCs we hypothesize, that these genes act in different pathways. The observed upregulation of *Pten* in *Tfap2c^−/−^*PGCLCs is most likely due to induction of somatic differentiation, since *Pten* has been associated with development of neuronal and synaptic structures [70].

In *Tfap2c^+/−^* male gonads foci with cells displaying continued expression of pluripotency markers *Oct/4*, *Ssea1*, *Sox2* and *Nanog* were observed. In fact, this represents a hallmark critical for transformation of PGCs into embryonal carcinoma (EC) cells between E11.5–13.5 in mice [65]. Usually postmigratory PGCs downregulate pluripotency markers and enter mitotic arrest. However, EC cells show a block of germ cell differentiation and continued proliferation and expression of pluripotency markers also seen in human pediatric cancer.

**Figure 6:**
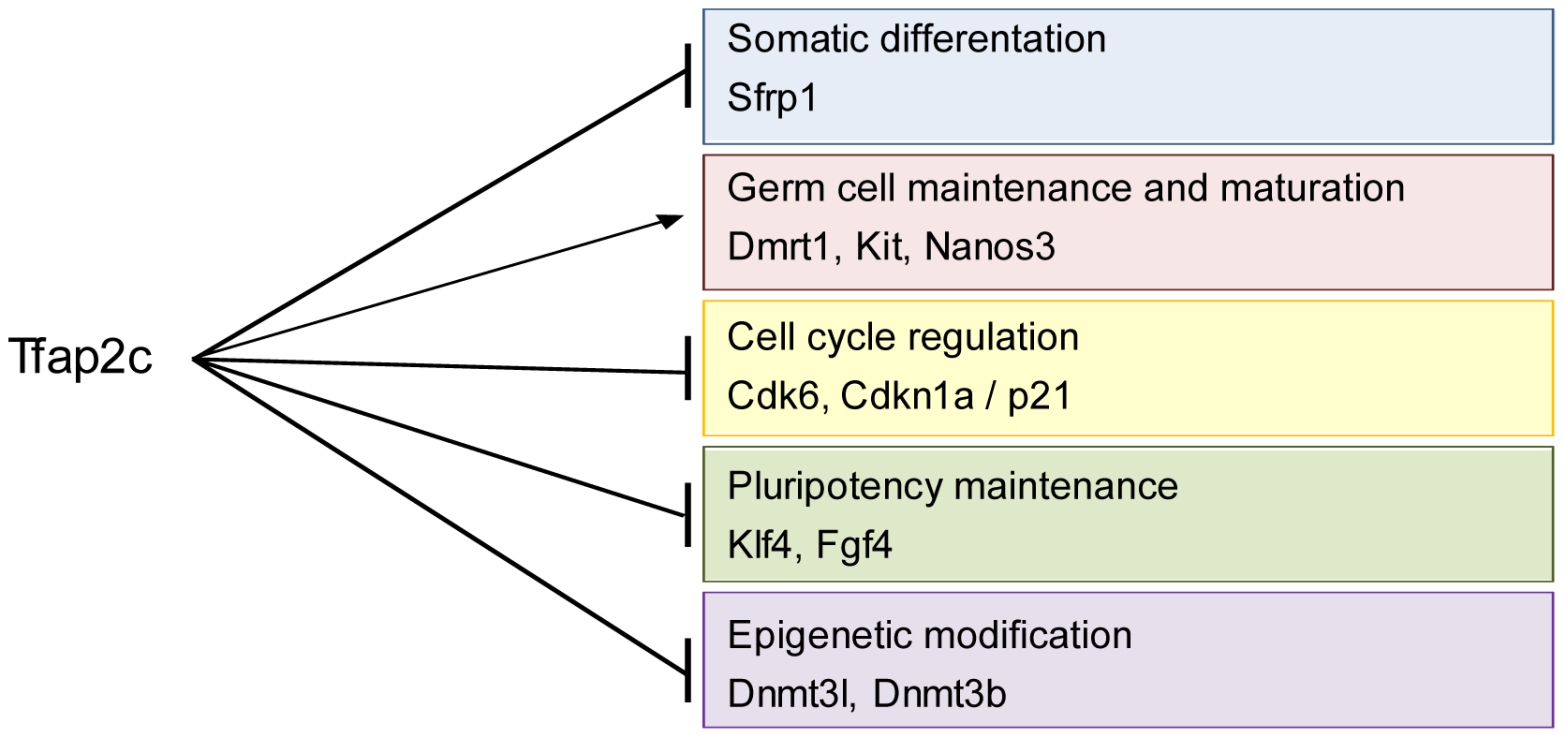
Schematic of the genes and programs regulated by TFAP2C in primordial germ cells. Black arrows indicate pathways transactivated and induced by TFAP2C and black lines with terminal bars indicate pathways repressed by TFAP2C during development of primordial germ cells. Genes listed in respective pathways indicate direct regulation as demonstrated by ChIP analyses.

In conclusion we provide evidence that the transcription factor TFAP2C orchestrates many aspects of the genetic network regulating germ cell development (Fig. 6). Maintenance and maturation of PGC are controlled by TFAP2C by repression of somatic differentiation, cell cycle, epigenetic remodeling, and pluripotency associated genes (Fig. 6). Furthermore, mice heterozygous for *Tfap2c* or its downstream target *Nanos3* display an increase in GCC susceptibility.

## Supporting Information

Figure S1 **Generation of** ***Tfap2c^ctrl^*** **and** ***Tfap2c^−/−^*** **ESCs.** (A) Immunofluorescence staining against TFAP2C and SSEA1 protein in *ctrl* and *Tfap2c^−/−^*ESCs (cell line #1 and #2). Nuclei are stained with Hoechst. Scale bars: 100 µm. (B) Genotyping of *Blimp1mVenus/Tfap2c^flox/flox^* and *Blimp1mVenus/Tfap2c^−/−^* ESC lines by PCR. (C) FACS analysis to identify *Blimp1mVenus* positive PGCLCs. Population P3 showing *mVenus* positive cells. 8.1% *Blimp1mVenus* positive cells in ctrl whereas 3.2% positive cells measured in *Tfap2c^−/−^* cells.
(TIF)

Figure S2 ***In vitro*** **differentiation of** ***Tfap2c^−/−^*** **and** ***ctrl*** **PGCLCs.** (A) Brightfield pictures of #1-*ctrl* ESCs and after 1 and 2 days of *in vitro* differentiation into EpiLCs. EpiLCs show a flattened epithelial-like structure (A, middle). Scale bars: 100 µm (B) ESC and Epiblast markers were analysed by RT-PCR of RNA from #1-*ctrl*; #1-*Tfap2c^−/−^* and #2-*ctrl*; #2-*Tfap2c^−/−^* ESCs and EpiLCs. *β-Actin* served as control. (C) Brightfield pictures show #1-*ctrl* and #1-*Tfap2c^−/−^* EBs. PGCLC induction is indicated by *Blimp1mVenus* fluorescence. Scale bars: 100 µm (D) Graph showing the percentage and standard deviation of PGCLCs generation efficiency, measured by FACS for #1-*ctrl* (9.3%) and #2-*ctrl* (3%). Efficiency of PGCLC formation is lower in *Tfap2c^−/−^* cells (#1∶2.7% and #2∶1.5%). Significance * *P*≤0.05. (E) RT-PCR from RNA of #1-*ctrl*; #1-*Tfap2c^−/−^* and #2-*ctrl*; #2-*Tfap2c^−/−^* PGCLCs. Expression of early germ cell markers (*Stella/Dppa3*, *Prdm14*, *Nanos3*) is reduced in *Tfap2c^−/−^*PGCLCs. As expected no signal was detected in *Tfap2c^−/−^*PGCLCs. *β-Actin* served as control.
(TIF)

Table S1 Deregulated genes in *Tfap2c^−/−^*PGCLCs (Fold-change >1.5 in log2 scale).
(XLSX)

Table S2 Deregulated genes in *Tfap2c^−/−^*ESCs (Fold-change >1.5 in log2 scale).
(XLSX)

Table S3 Commonly deregulated genes in *Tfap2c^−/−^*ESCs and -PGCLCs (Fold-change >1.5 in log2 scale).
(XLSX)

Table S4 Location of TFAP2/TFAP2C binding sites and primer sequences.
(XLSX)

Table S5 Primer sequences for RT-PCR.
(DOCX)

Table S6 Primer sequences for qRT-PCR.
(DOCX)

Table S7 List of abbreviations.
(DOCX)

Data S1 Gene Ontology analysis for >1.5 fold-change in log2 scale for *Tfap2c^−/−^*PGCLCs in comparison to *ctrl* PGCLCs.
(PDF)

## References

[1] Oosterhuis JW, Looijenga LHJ (2005) Testicular germ-cell tumours in a broader perspective. Nature Reviews Cancer 5: 210–222. Available: http://www.ncbi.nlm.nih.gov/pubmed/15738984. Accessed 8 March 2013. 10.1038/nrc1568 15738984

[2] Schneider DT, Calaminus G, Koch S, Teske C, Schmidt P, et al. (2004) Epidemiologic analysis of 1,442 children and adolescents registered in the German germ cell tumor protocols. Pediatric blood & cancer 42: 169–175. Available: http://www.ncbi.nlm.nih.gov/pubmed/14752882. Accessed 22 March 2013. 10.1002/pbc.10321 14752882

[3] Motzer RJ, Amsterdam a, Prieto V, Sheinfeld J, Murty V V, et al (1998) Teratoma with malignant transformation: diverse malignant histologies arising in men with germ cell tumors. The Journal of urology 159: 133–138 Available: http://www.ncbi.nlm.nih.gov/pubmed/9400455. 9400455 10.1016/s0022-5347(01)64035-7

[4] Ulbright TM (2005) Germ cell tumors of the gonads: a selective review emphasizing problems in differential diagnosis, newly appreciated, and controversial issues. Modern pathology: an official journal of the United States and Canadian Academy of Pathology, Inc 18 Suppl 2: S61–79. Available: http://www.ncbi.nlm.nih.gov/pubmed/15761467. Accessed 27 February 2013. 10.1038/modpathol.3800310 15761467

[5] Pierce GB, Stevens LC, Nakane PK (1967) Ultrastructural Analysis of the Early Development of Teratocarinomas. Journal of the National Cancer Institute Vol 39: 755–773. 18623935

[6] Lawson K a, Dunn NR, Roelen B a J, Zeinstra LM, Davis AM, et al (1999) Bmp4 is required for the generation of primordial germ cells in the mouse embryo. Genes & Development 13: 424–436 Available: http://www.pubmedcentral.nih.gov/articlerender.fcgi?artid=316469&tool=pmcentrez&rendertype=abstract. 10049358 10.1101/gad.13.4.424 PMC316469

[7] Ying Y, Liu XM, Marble a, Lawson K a, Zhao GQ (2000) Requirement of Bmp8b for the generation of primordial germ cells in the mouse. Molecular endocrinology (Baltimore, Md) 14: 1053–1063 Available: http://www.ncbi.nlm.nih.gov/pubmed/10894154. 10.1210/mend.14.7.0479 10894154

[8] Ying Y, Zhao GQ (2001) Cooperation of endoderm-derived BMP2 and extraembryonic ectoderm-derived BMP4 in primordial germ cell generation in the mouse. Developmental biology 232: 484–492. Available: http://www.ncbi.nlm.nih.gov/pubmed/11401407. Accessed 24 March 2013. 10.1006/dbio.2001.0173 11401407

[9] Ohinata Y, Payer B, O’Carroll D, Ancelin K, Ono Y, et al. (2005) Blimp1 is a critical determinant of the germ cell lineage in mice. Nature 436: 207–213. Available: http://www.ncbi.nlm.nih.gov/pubmed/15937476. Accessed 5 March 2013. 10.1038/nature03813 15937476

[10] Kurimoto K, Yabuta Y, Ohinata Y, Shigeta M, Yamanaka K, et al. (2008) Complex genome-wide transcription dynamics orchestrated by Blimp1 for the specification of the germ cell lineage in mice. Genes & Development 22: 1617–1635. Available: http://www.pubmedcentral.nih.gov/articlerender.fcgi?artid=2428060&tool=pmcentrez&rendertype=abstract. Accessed 8 March 2013. 10.1101/gad.1649908 PMC2428060 18559478

[11] Yamaji M, Seki Y, Kurimoto K, Yabuta Y, Yuasa M, et al. (2008) Critical function of Prdm14 for the establishment of the germ cell lineage in mice. Nature Genetics 40: 1016–1022. Available: http://www.ncbi.nlm.nih.gov/pubmed/18622394. Accessed 8 March 2013. 10.1038/ng.186 18622394

[12] Eckert D, Buhl S, Weber S, Jäger R, Schorle H (2005) The AP-2 family of transcription factors. Genome Biology 6: 246. Available: http://www.pubmedcentral.nih.gov/articlerender.fcgi?artid=1414101&tool=pmcentrez&rendertype=abstract. Accessed 8 March 2013. 10.1186/gb-2005-6-13-246 PMC1414101 16420676

[13] Weber S, Eckert D, Nettersheim D, Gillis AJM, Schäfer S, et al. (2010) Critical function of AP-2 gamma/TCFAP2C in mouse embryonic germ cell maintenance. Biology of Reproduction 82: 214–223. Available: http://www.ncbi.nlm.nih.gov/pubmed/19776388. Accessed 8 March 2013. 10.1095/biolreprod.109.078717 19776388

[14] Yabuta Y, Kurimoto K, Ohinata Y, Seki Y, Saitou M (2006) Gene expression dynamics during germline specification in mice identified by quantitative single-cell gene expression profiling. Biology of Reproduction 75: 705–716. Available: http://www.ncbi.nlm.nih.gov/pubmed/16870942. Accessed 8 March 2013. 10.1095/biolreprod.106.053686 16870942

[15] Western PS, Miles DC, Van den Bergen J a, Burton M, Sinclair AH (2008) Dynamic regulation of mitotic arrest in fetal male germ cells. Stem cells (Dayton, Ohio) 26: 339–347. Available: http://www.ncbi.nlm.nih.gov/pubmed/18024419. Accessed 2 March 2013. 10.1634/stemcells.2007-0622 18024419

[16] Toyooka Y, Tsunekawa N, Takahashi Y, Matsui Y, Satoh M, et al (2000) Expression and intracellular localization of mouse Vasa-homologue protein during germ cell development. Mechanisms of development 93: 139–149 Available: http://www.ncbi.nlm.nih.gov/pubmed/10781947. 10781947 10.1016/s0925-4773(00)00283-5

[17] Seligman J, Page DC (1998) The Dazh gene is expressed in male and female embryonic gonads before germ cell sex differentiation. Biochemical and biophysical research communications 245: 878–882 Available: http://www.ncbi.nlm.nih.gov/pubmed/9588208. 9588208 10.1006/bbrc.1998.8530

[18] Seki Y, Yamaji M, Yabuta Y, Sano M, Shigeta M, et al. (2007) Cellular dynamics associated with the genome-wide epigenetic reprogramming in migrating primordial germ cells in mice. Development (Cambridge, England) 134: 2627–2638. Available: http://www.ncbi.nlm.nih.gov/pubmed/17567665. Accessed 28 February 2013. 10.1242/dev.005611 17567665

[19] Hoei-Hansen CE, Nielsen JE, Almstrup K, Sonne SB, Graem N, et al (2004) Transcription factor AP-2gamma is a developmentally regulated marker of testicular carcinoma in situ and germ cell tumors. Clinical Cancer Research 10: 8521–8530 Available: http://www.ncbi.nlm.nih.gov/pubmed/15623634. 15623634 10.1158/1078-0432.CCR-04-1285

[20] Pauls K, Jäger R, Weber S, Wardelmann E, Koch A, et al. (2005) Transcription factor AP-2gamma, a novel marker of gonocytes and seminomatous germ cell tumors. International journal of cancer Journal international du cancer 115: 470–477. Available: http://www.ncbi.nlm.nih.gov/pubmed/15700319. Accessed 8 March 2013. 10.1002/ijc.20913 15700319

[21] Ohinata Y, Sano M, Shigeta M, Yamanaka K, Saitou M (2008) A comprehensive, non-invasive visualization of primordial germ cell development in mice by the Prdm1-mVenus and Dppa3-ECFP double transgenic reporter. Reproduction Cambridge England 136: 503–514. Available: http://www.ncbi.nlm.nih.gov/pubmed/18583473. Accessed 8 March 2013. 10.1530/REP-08-0053 18583473

[22] Werling U, Schorle H (2002) Conditional inactivation of transcription factor AP-2gamma by using the Cre/loxP recombination system. Genesis (New York, NY: 2000) 32: 127–129. Available: http://www.ncbi.nlm.nih.gov/pubmed/11857798. Accessed 8 March 2013. 10.1002/gene.10057 11857798

[23] Kuckenberg P, Buhl S, Woynecki T, Van Fürden B, Tolkunova E, et al. (2010) The transcription factor TCFAP2C/AP-2gamma cooperates with CDX2 to maintain trophectoderm formation. Molecular and Cellular Biology 30: 3310–3320. Available: http://www.ncbi.nlm.nih.gov/pubmed/20404091. Accessed 8 March 2013. 10.1128/MCB.01215-09 PMC2897582 20404091

[24] Hayashi K, Ohta H, Kurimoto K, Aramaki S, Saitou M (2011) Reconstitution of the mouse germ cell specification pathway in culture by pluripotent stem cells. Cell 146: 519–532. Available: http://linkinghub.elsevier.com/retrieve/pii/S0092867411007719. Accessed 5 March 2013. 10.1016/j.cell.2011.06.052 21820164

[25] Payer B, Chuva De Sousa Lopes SM, Barton SC, Lee C, Saitou M, et al. (2006) Generation of stella-GFP transgenic mice: a novel tool to study germ cell development. Genesis New York NY 2000 44: 75–83. Available: http://www.ncbi.nlm.nih.gov/pubmed/16437550. Accessed 8 March 2013. 10.1002/gene.20187 16437550

[26] Young JC, Dias VL, Loveland KL (2010) Defining the window of germline genesis in vitro from murine embryonic stem cells. Biology of Reproduction 82: 390–401. Available: http://www.ncbi.nlm.nih.gov/pubmed/19846600. Accessed 8 March 2013. 10.1095/biolreprod.109.078493 19846600

[27] Nettersheim D, Gillis A, Biermann K, Looijenga LHJ, Schorle H (2011) The Seminoma Cell Line TCam-2 is Sensitive to HDAC Inhibitor Depsipeptide but Tolerates Various Other Chemotherapeutic Drugs and Loss of NANOG Expression. 1042: 1033–1042 doi:10.1002/gcc 10.1002/gcc.20918 21987446

[28] Lim S, Janzer A, Becker A, Zimmer A, Schüle R, et al. (2010) Lysine-specific demethylase 1 (LSD1) is highly expressed in ER-negative breast cancers and a biomarker predicting aggressive biology. Carcinogenesis 31: 512–520. Available: http://www.ncbi.nlm.nih.gov/pubmed/20042638. Accessed 28 February 2013. 10.1093/carcin/bgp324 20042638

[29] Irizarry R a, Hobbs B, Collin F, Beazer-Barclay YD, Antonellis KJ, et al (2003) Exploration, normalization, and summaries of high density oligonucleotide array probe level data. Biostatistics (Oxford, England) 4: 249–264 Available: http://www.ncbi.nlm.nih.gov/pubmed/12925520. 10.1093/biostatistics/4.2.249 12925520

[30] Ashburner M, Ball C, Blake J, Botstein D (2000) Gene Ontology: tool for the unification of biology. Nature … 25: 25–29. Available: http://www.nature.com/ng/journal/v25/n1/abs/ng0500_25.html. Accessed 7 April 2013. 10.1038/75556 PMC3037419 10802651

[31] Eckert D, Nettersheim D, Heukamp LC, Kitazawa S, Biermann K, et al. (2008) TCam-2 but not JKT-1 cells resemble seminoma in cell culture. Cell and Tissue Research 331: 529–538. Available: http://www.ncbi.nlm.nih.gov/pubmed/18008088. Accessed 8 March 2013. 10.1007/s00441-007-0527-y 18008088

[32] Ovcharenko I, Nobrega M a, Loots GG, Stubbs L (2004) ECR Browser: a tool for visualizing and accessing data from comparisons of multiple vertebrate genomes. Nucleic acids research 32: W280–6. Available: http://www.pubmedcentral.nih.gov/articlerender.fcgi?artid=441493&tool=pmcentrez&rendertype=abstract. Accessed 28 February 2013. 10.1093/nar/gkh355 PMC441493 15215395

[33] Rivers EN, Hamilton DW (1986) Morphologic analysis of spontaneous teratocarcinogenesis in developing testes of strain 129/Sv-ter mice. The American journal of pathology 124: 263–280 Available: http://www.pubmedcentral.nih.gov/articlerender.fcgi?artid=1888294&tool=pmcentrez&rendertype=abstract. 3740215 PMC1888294

[34] Suzuki H, Tsuda M, Kiso M, Saga Y (2008) Nanos3 maintains the germ cell lineage in the mouse by suppressing both Bax-dependent and -independent apoptotic pathways. Developmental Biology 318: 133–142. Available: http://www.ncbi.nlm.nih.gov/pubmed/18436203. Accessed 8 March 2013. 10.1016/j.ydbio.2008.03.020 18436203

[35] Schäfer S, Anschlag J, Nettersheim D, Haas N, Pawig L, et al. (2011) The role of BLIMP1 and its putative downstream target TFAP2C in germ cell development and germ cell tumours. International Journal of Andrology 34: e152–e158; discussion e158–e159. Available: http://www.ncbi.nlm.nih.gov/pubmed/21564135. Accessed 8 March 2013. 10.1111/j.1365-2605.2011.01167.x 21564135

[36] Stevens L (1964) Experimental production of testicular teratomas in mice. Proceedings of the National Academy of Sciences of …: 654–661. Available: http://www.ncbi.nlm.nih.gov/pmc/articles/PMC300322/. Accessed 7 April 2013. 10.1073/pnas.52.3.654 PMC300322 14212538

[37] Misra K, Matise MP (2010) A critical role for sFRP proteins in maintaining caudal neural tube closure in mice via inhibition of BMP signaling. Developmental biology 337: 74–83. Available: http://www.ncbi.nlm.nih.gov/pubmed/19850029. Accessed 5 April 2013. 10.1016/j.ydbio.2009.10.015 19850029

[38] Beumer TL, Roepers-Gajadien HL, Gademan IS, Kal HB, De Rooij DG (2000) Involvement of the D-type cyclins in germ cell proliferation and differentiation in the mouse. Biology of reproduction 63: 1893–1898 Available: http://www.ncbi.nlm.nih.gov/pubmed/11090462. 11090462 10.1095/biolreprod63.6.1893

[39] Heaney JD, Anderson EL, Michelson M V, Zechel JL, Conrad P a, et al. (2012) Germ cell pluripotency, premature differentiation and susceptibility to testicular teratomas in mice. Development (Cambridge, England) 139: 1577–1586. Available: http://www.pubmedcentral.nih.gov/articlerender.fcgi?artid=3317965&tool=pmcentrez&rendertype=abstract. Accessed 8 March 2013. 10.1242/dev.076851 PMC3317965 22438569

[40] Deshpande A, Sicinski P, Hinds PW (2005) Cyclins and cdks in development and cancer: a perspective. Oncogene 24: 2909–2915. Available: http://www.ncbi.nlm.nih.gov/pubmed/15838524. Accessed 13 March 2013. 10.1038/sj.onc.1208618 15838524

[41] Musgrove E a, Lee CS, Buckley MF, Sutherland RL (1994) Cyclin D1 induction in breast cancer cells shortens G1 and is sufficient for cells arrested in G1 to complete the cell cycle. Proceedings of the National Academy of Sciences of the United States of America 91: 8022–8026 Available: http://www.pubmedcentral.nih.gov/articlerender.fcgi?artid=44537&tool=pmcentrez&rendertype=abstract. 8058751 10.1073/pnas.91.17.8022 PMC44537

[42] Filipczyk A a, Laslett AL, Mummery C, Pera MF (2007) Differentiation is coupled to changes in the cell cycle regulatory apparatus of human embryonic stem cells. Stem cell research 1: 45–60. Available: http://www.ncbi.nlm.nih.gov/pubmed/19383386. Accessed 10 March 2013. 10.1016/j.scr.2007.09.002 19383386

[43] Singh AM, Dalton S (2009) The cell cycle and Myc intersect with mechanisms that regulate pluripotency and reprogramming. Cell stem cell 5: 141–149. Available: http://www.pubmedcentral.nih.gov/articlerender.fcgi?artid=2909475&tool=pmcentrez&rendertype=abstract. Accessed 3 April 2013. 10.1016/j.stem.2009.07.003 PMC2909475 19664987

[44] Williams CMJ, Scibetta AG, Friedrich JK, Canosa M, Berlato C, et al. (2009) AP-2gamma promotes proliferation in breast tumour cells by direct repression of the CDKN1A gene. the The European Molecular Biology Organization Journal 28: 3591–3601. Available: http://www.pubmedcentral.nih.gov/articlerender.fcgi?artid=2782101&tool=pmcentrez&rendertype=abstract. Accessed 8 March 2013. 10.1038/emboj.2009.290 PMC2782101 19798054

[45] Deng C, Zhang P, Harper JW, Elledge SJ, Leder P (1995) Mice Lacking p21 c ∼ P7/wAF7 Undergo Normal Development, in Gl Checkpoint Control but Are Defective. 82: 875–884. 10.1016/0092-8674(95)90039-x 7664346

[46] Iii ABN, Chen X, Smeets M, Hengst L, Prives C, et al.. (1998) Effects of p21 Cip1/Waf1 at Both the G 1/S and the G 2/M Cell Cycle Transitions: pRb Is a Critical Determinant in Blocking DNA Replication and in Preventing Endoreduplication Effects of p21 Cip1/Waf1 at Both the G 1/S and the G 2/M Cell Cycle. 10.1128/mcb.18.1.629 PMC121530 9418909

[47] Ogryzko V V, Wong P, Howard BH, Ogryzko V V, Wong P, et al.. (1997) WAF1 retards S-phase progression primarily by inhibition of cyclin-dependent kinases. WAF1 Retards S-Phase Progression Primarily by Inhibition of Cyclin-Dependent Kinases. 17. 10.1128/mcb.17.8.4877 PMC232340 9234744

[48] Helt C, Rancourt R (2001) p53-dependent induction of p21Cip1/WAF1/Sdi1 protects against oxygen-induced toxicity. Toxicological … 222: 214–222. Available: http://toxsci.oxfordjournals.org/content/63/2/214.short. Accessed 7 April 2013. 10.1093/toxsci/63.2.214 11568365

[49] Javelaud D, Wietzerbin J, Delattre O, Besanc Ë (2000) Induction of p21 Waf1/Cip1 by TNFa requires NF-kB activity and antagonizes apoptosis in Ewing tumor cells. Oncogene 1: 61–68. 10.1038/sj.onc.1203246 10644980

[50] Cook MS, Coveney D, Batchvarov I, Nadeau JH, Capel B (2009) BAX-mediated cell death affects early germ cell loss and incidence of testicular teratomas in Dnd1(Ter/Ter) mice. Developmental biology 328: 377–383. Available: http://www.pubmedcentral.nih.gov/articlerender.fcgi?artid=2689365&tool=pmcentrez&rendertype=abstract. Accessed 7 April 2013. 10.1016/j.ydbio.2009.01.041 PMC2689365 19389346

[51] Krentz AD, Murphy MW, Kim S, Cook MS, Capel B, et al (2009) The DM domain protein DMRT1 is a dose-sensitive regulator of fetal germ cell proliferation and pluripotency. Proceedings of the National Academy of Sciences of the United States of America 106: 22323–22328 Available: http://www.pubmedcentral.nih.gov/articlerender.fcgi?artid=2799724&tool=pmcentrez&rendertype=abstract. 20007774 10.1073/pnas.0905431106 PMC2799724

[52] Sharov A a, Masui S, Sharova L V, Piao Y, Aiba K, et al. (2008) Identification of Pou5f1, Sox2, and Nanog downstream target genes with statistical confidence by applying a novel algorithm to time course microarray and genome-wide chromatin immunoprecipitation data. BMC genomics 9: 269. Available: http://www.pubmedcentral.nih.gov/articlerender.fcgi?artid=2424064&tool=pmcentrez&rendertype=abstract. Accessed 24 March 2013. 10.1186/1471-2164-9-269 PMC2424064 18522731

[53] Takahashi K, Mitsui K, Yamanaka S (2003) Role of ERas in promoting tumour-like properties in mouse embryonic stem cells. 423: 541–545 doi:10.1038/nature01633.1 10.1038/nature01646 12774123

[54] Hackett J a, Zylicz JJ, Surani MA (2012) Parallel mechanisms of epigenetic reprogramming in the germline. Trends in genetics: TIG 28: 164–174. Available: http://www.ncbi.nlm.nih.gov/pubmed/22386917. Accessed 4 April 2013. 10.1016/j.tig.2012.01.005 22386917

[55] Sasaki H, Matsui Y (2008) Epigenetic events in mammalian germ-cell development: reprogramming and beyond. Nature Reviews Genetics 9: 129–140. Available: http://www.ncbi.nlm.nih.gov/pubmed/18197165. Accessed 27 February 2013. 10.1038/nrg2295 18197165

[56] Seki Y, Hayashi K, Itoh K, Mizugaki M, Saitou M, et al. (2005) Extensive and orderly reprogramming of genome-wide chromatin modifications associated with specification and early development of germ cells in mice. Developmental biology 278: 440–458. Available: http://www.ncbi.nlm.nih.gov/pubmed/15680362. Accessed 5 March 2013. 10.1016/j.ydbio.2004.11.025 15680362

[57] Kurimoto K, Yamaji M, Seki Y, Saitou M (2008) Specification of the germ cell lineage in mice ND ES NO ST ND ES OS NO ST. Cell Cycle 7: 3514–3518 Available: http://www.landesbioscience.com/journals/cbt/KurimotoCC7-22.pdf. 19001867 10.4161/cc.7.22.6979

[58] Bostick M, Kim JK, Estève P-O, Clark A, Pradhan S, et al. (2007) UHRF1 plays a role in maintaining DNA methylation in mammalian cells. Science (New York, NY) 317: 1760–1764. Available: http://www.ncbi.nlm.nih.gov/pubmed/17673620. Accessed 5 March 2013. 10.1126/science.1147939 17673620

[59] Kato Y, Kaneda M, Hata K, Kumaki K, Hisano M, et al. (2007) Role of the Dnmt3 family in de novo methylation of imprinted and repetitive sequences during male germ cell development in the mouse. Human molecular genetics 16: 2272–2280. Available: http://www.ncbi.nlm.nih.gov/pubmed/17616512. Accessed 5 March 2013. 10.1093/hmg/ddm179 17616512

[60] Liao H-F, Tai K-Y, Chen WS-C, Cheng LCW, Ho H-N, et al. (2012) Functions of DNA methyltransferase 3-like in germ cells and beyond. Biology of the cell under the auspices of the European Cell Biology Organization 104: 571–587. Available: http://www.ncbi.nlm.nih.gov/pubmed/22671959. Accessed 1 March 2013. 10.1111/boc.201100109 22671959

[61] Skotheim RI, Lind GE, Monni O, Nesland JM, Abeler VM, et al. (2005) Differentiation of human embryonal carcinomas in vitro and in vivo reveals expression profiles relevant to normal development. Cancer research 65: 5588–5598. Available: http://www.ncbi.nlm.nih.gov/pubmed/15994931. Accessed 14 March 2013. 10.1158/0008-5472.CAN-05-0153 15994931

[62] Sperger JM, Chen X, Draper JS, Antosiewicz JE, Chon CH, et al (2003) Gene expression patterns in human embryonic stem cells and human pluripotent germ cell tumors. Proceedings of the National Academy of Sciences of the United States of America 100: 13350–13355 Available: http://www.pubmedcentral.nih.gov/articlerender.fcgi?artid=263817&tool=pmcentrez&rendertype=abstract. 14595015 10.1073/pnas.2235735100 PMC263817

[63] Almstrup K, Hoei-Hansen CE, Nielsen JE, Wirkner U, Ansorge W, et al. (2005) Genome-wide gene expression profiling of testicular carcinoma in situ progression into overt tumours. British journal of cancer 92: 1934–1941. Available: http://www.pubmedcentral.nih.gov/articlerender.fcgi?artid=2361756&tool=pmcentrez&rendertype=abstract. Accessed 10 June 2013. 10.1038/sj.bjc.6602560 PMC2361756 15856041

[64] Biermann K, Heukamp LC, Steger K, Zhou H, Franke FE, et al (2007) Genome-wide expression profiling reveals new insights into pathogenesis and progression of testicular germ cell tumors. Cancer genomics & proteomics 4: 359–367 Available: http://www.ncbi.nlm.nih.gov/pubmed/17993720. 17993720

[65] Stevens L (1967) Origin of testicular teratomas from primordial germ cells in mice. Journal of the National Cancer Institute: 549–552. Available: http://jnci.oxfordjournals.org/content/38/4/549.short. Accessed 5 April 2013. 6025005

[66] Krentz AD, Murphy MW, Zhang T, Sarver AL, Jain S, et al. (2013) Interaction between DMRT1 function and genetic background modulates signaling and pluripotency to control tumor susceptibility in the fetal germ line. Developmental biology: 1–13. Available: http://www.ncbi.nlm.nih.gov/pubmed/23473982. Accessed 16 March 2013. 10.1016/j.ydbio.2013.02.014 PMC3630265 23473982

[67] Kimura T (2003) Conditional loss of PTEN leads to testicular teratoma and enhances embryonic germ cell production. Development 130: 1691–1700. Available: http://dev.biologists.org/cgi/doi/10.1242/dev.00392. Accessed 7 April 2013. 10.1242/dev.00392 12620992

[68] Youngren KK, Coveney D, Peng X, Bhattacharya C, Schmidt LS, et al. (2005) The Ter mutation in the dead end gene causes germ cell loss and testicular germ cell tumours. Nature 435: 360–364. Available: http://www.pubmedcentral.nih.gov/articlerender.fcgi?artid=1421521&tool=pmcentrez&rendertype=abstract. Accessed 8 April 2013. 10.1038/nature03595 PMC1421521 15902260

[69] Noguchi T, Noguchi M (1985) A recessive mutation (ter) causing germ cell deficiency and a high incidence of congenital testicular teratomas in 129/Sv-ter mice. Journal of the National Cancer Institute 75: 385–392 Available: http://www.ncbi.nlm.nih.gov/pubmed/3860691. 3860691

[70] Fraser MM, Bayazitov IT, Zakharenko SS, Baker SJ (2008) Phosphatase and tensin homolog, deleted on chromosome 10 deficiency in brain causes defects in synaptic structure, transmission and plasticity, and myelination abnormalities. Neuroscience 151: 476–488. Available: http://www.pubmedcentral.nih.gov/articlerender.fcgi?artid=2278004&tool=pmcentrez&rendertype=abstract. Accessed 8 April 2013. 10.1016/j.neuroscience.2007.10.048 PMC2278004 18082964

