# Correction: Transcription Factor TFAP2C Regulates Major Programs Required for Murine Fetal Germ Cell Maintenance and Haploinsufficiency Predisposes to Teratomas in Male Mice

**DOI:** 10.1371/annotation/579ac618-0ed0-4451-a4fc-10bad989024b

**Published:** 2013-08-29

**Authors:** Jana Schemmer, Marcos J. Araúzo-Bravo, Natalie Haas, Sabine Schäfer, Susanne N. Weber, Astrid Becker, Dawid Eckert, Andreas Zimmer, Daniel Nettersheim, Hubert Schorle

The affiliations of authors Astrid Becker and Andreas Zimmer were incorrect. These author are instead affiliated with:

University of Bonn, Institute of Molecular Psychiatry, Bonn, Germany 

