# Supplementary figures and images for: Transcription Factor TFAP2C Regulates Major Programs Required for Murine Fetal Germ Cell Maintenance and Haploinsufficiency Predisposes to Teratomas in Male Mice
Source: PLoS One. 2013 Aug 13;8(8):e71113. doi: 10.1371/journal.pone.0071113 (PMC3742748; doi:10.1371/journal.pone.0071113)

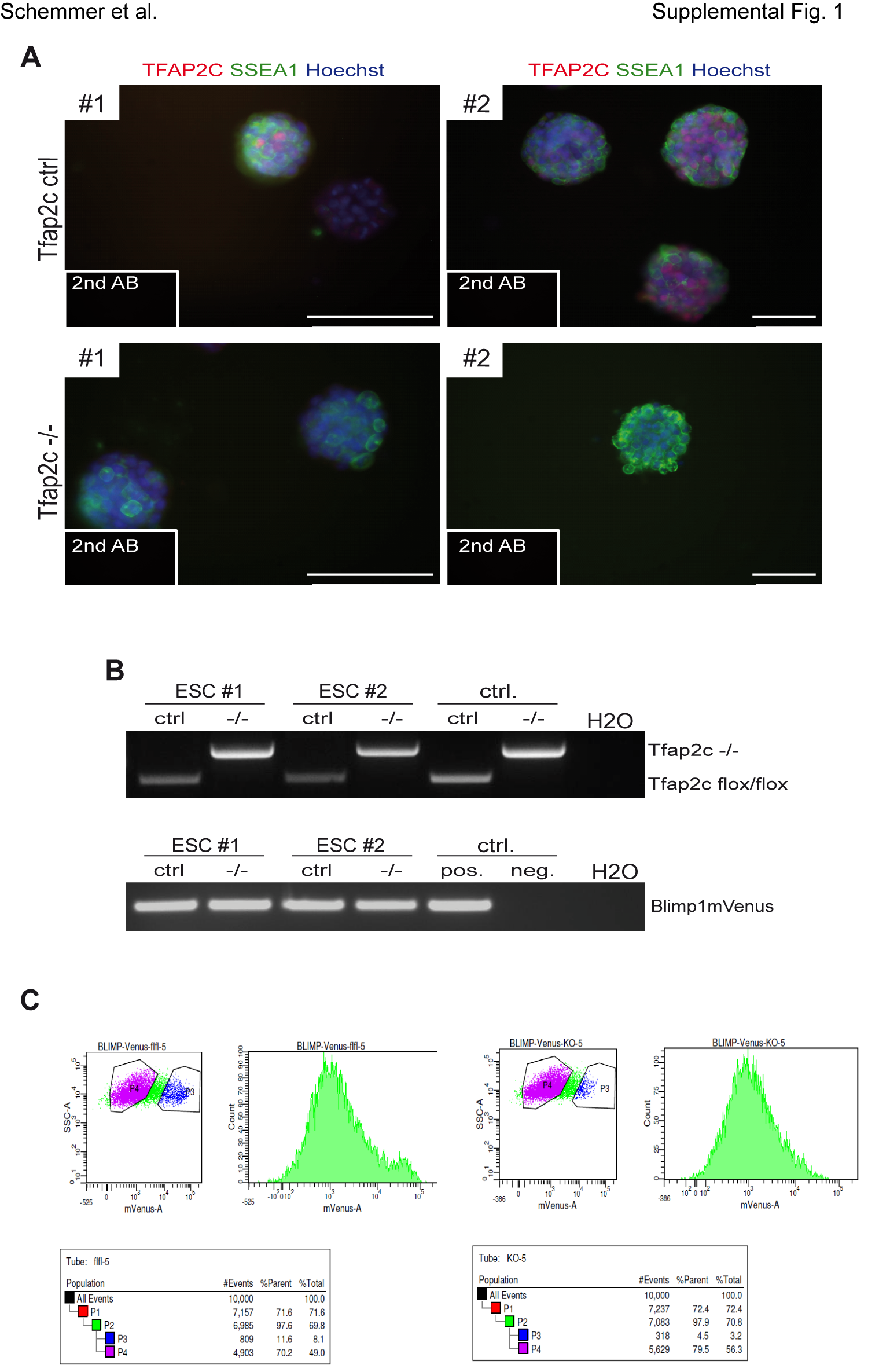

Supplement: Figure S1 — Generation of Tfap2cctrl and Tfap2c−/− ESCs. (A) Immunofluorescence staining against TFAP2C and SSEA1 protein in ctrl and Tfap2c−/−ESCs (cell line #1 and #2). Nuclei are stained with Hoechst. Scale bars: 100 µm. (B) Genotyping of Blimp1mVenus/Tfap2cflox/flox and Blimp1mVenus/Tfap2c−/− ESC lines by PCR. (C) FACS analysis to identify Blimp1mVenus positive PGCLCs. Population P3 showing mVenus positive cells. 8.1% Blimp1mVenus positive cells in ctrl whereas 3.2% positive cells measured in Tfap2c−/− cells. (TIF) [file pone.0071113.s001.tif]

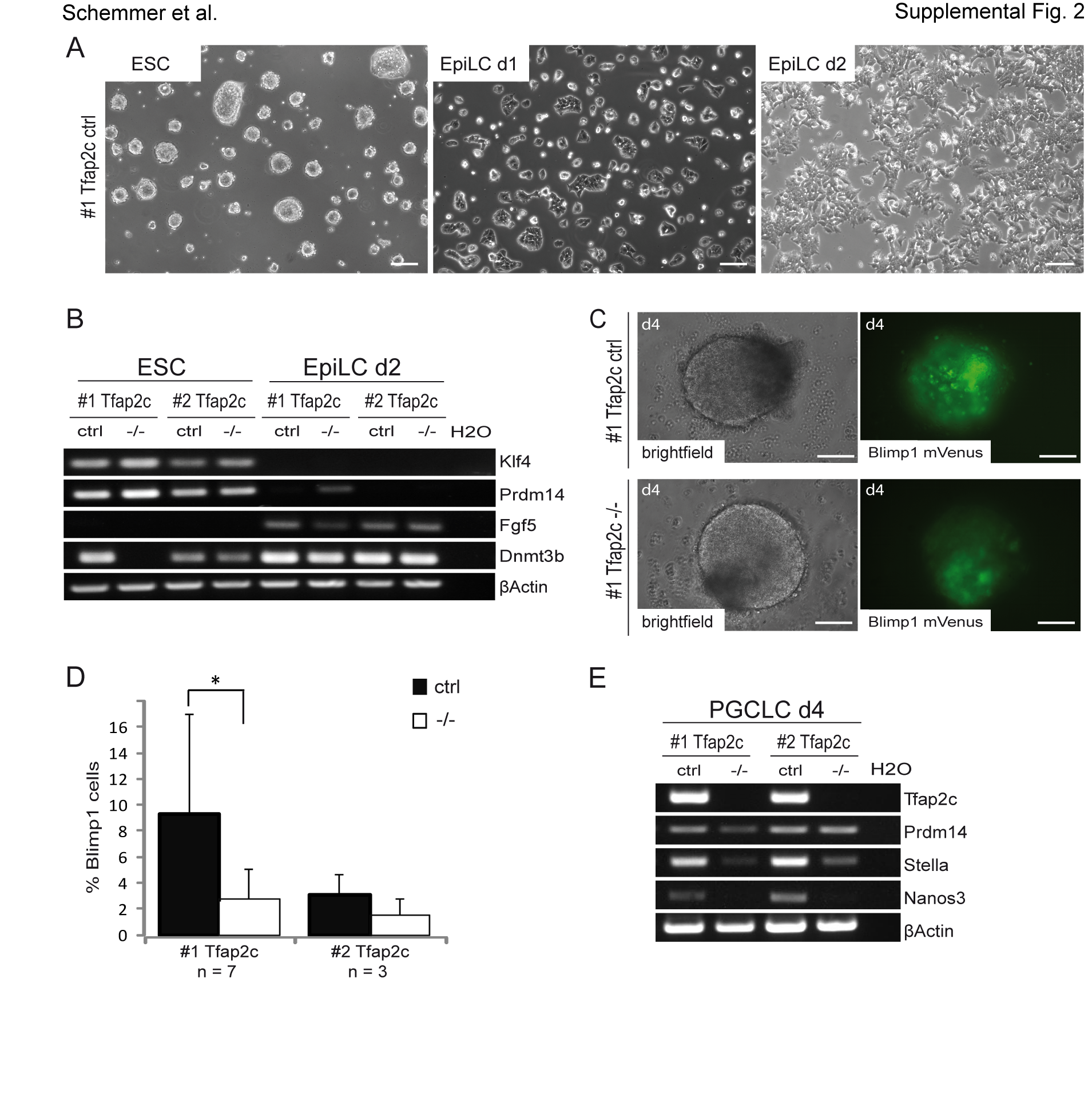

Supplement: Figure S2 — In vitro differentiation of Tfap2c−/− and ctrl PGCLCs. (A) Brightfield pictures of #1-ctrl ESCs and after 1 and 2 days of in vitro differentiation into EpiLCs. EpiLCs show a flattened epithelial-like structure (A, middle). Scale bars: 100 µm (B) ESC and Epiblast markers were analysed by RT-PCR of RNA from #1-ctrl; #1-Tfap2c−/− and #2-ctrl; #2-Tfap2c−/− ESCs and EpiLCs. β-Actin served as control. (C) Brightfield pictures show #1-ctrl and #1-Tfap2c−/− EBs. PGCLC induction is indicated by Blimp1mVenus fluorescence. Scale bars: 100 µm (D) Graph showing the percentage and standard deviation of PGCLCs generation efficiency, measured by FACS for #1-ctrl (9.3%) and #2-ctrl (3%). Efficiency of PGCLC formation is lower in Tfap2c−/− cells (#1∶2.7% and #2∶1.5%). Significance * P≤0.05. (E) RT-PCR from RNA of #1-ctrl; #1-Tfap2c−/− and #2-ctrl; #2-Tfap2c−/− PGCLCs. Expression of early germ cell markers (Stella/Dppa3, Prdm14, Nanos3) is reduced in Tfap2c−/−PGCLCs. As expected no signal was detected in Tfap2c−/−PGCLCs. β-Actin served as control. (TIF) [file pone.0071113.s002.tif]
