# Supplementary material for: Transcription Factor TFAP2C Regulates Major Programs Required for Murine Fetal Germ Cell Maintenance and Haploinsufficiency Predisposes to Teratomas in Male Mice
Source: PLoS One. 2013 Aug 13;8(8):e71113. doi: 10.1371/journal.pone.0071113 (PMC3742748; doi:10.1371/journal.pone.0071113)
Supplement: Table S5 — Primer sequences for RT-PCR. (DOCX) [file pone.0071113.s007.docx]

**Supplement-Table S5: mouse RT-PCR Primer**

| beta Act-F  beta Act-R | 5´- GGTCAGAAGGACTCCTATGT -3´  5´- ATGAGGTAGTCTGTCAGGTC -3´ |
| --- | --- |
| Blimp1-F  Blimp1-R | 5´-CACACAGGAGAGAAGCCACA -3´  5´- TCGAAGGTGGGTCTTGAGAT-3´ |
| c-Kit-F  c-Kit-R | 5´- AAGATGAACCCTCAGCCTCA -3´  5´- CGTCTCCTGGCGTTCATAAT-3´ |
| Dnmt3b-F  Dnmt3b-R | 5´- TGCGTCGTTCAGACAGTAGG -3´  5´- GCCCTTGTTGTTGGTGACTT -3´ |
| Dmrt1-F  Dmrt1-R | 5‘- AAGGCCCCTCCTACTCAGAA -3‘  5‘- GAGGGAGACCAAGCCAGAAT -3‘ |
| Fgf5-F  Fgf5-R | 5‘- TTGCGACCCAGGAGCTTAAT-3‘  5‘- CTACGCCTCTTTATTGCAGC-3‘ |
| Gapdh-F  Gapdh-R | 5‘- GGTGCTGAGTATGTCGTGGA- 3‘  5‘- TTGGCTCCACCCTTCAAGT- 3‘ |
| Klf4-F  Klf4-R | 5´- GCGAGTCTGACATGGCTGT -3´  5´- GAGTTCCTCACGCCAACG -3´ |
| Nanog-F  Nanog-R | 5‘- GGTGGCAGAAAAACCAGTG- 3‘  5‘- GCAATGGATGCTGGGATACT- 3‘ |
| Nanos3-F  Nanos3-R | 5´- TCCCGTGCCATCTATCAG -3´  5´- GGATGTTGAGGCAACACC -3´ |
| Oct3/4-F  Oct3/4-R | 5´- CTAGCATTGAGAACCGTGTGAG -3´  5´- GAAAGGTGTCCTGTAGCCTCAT -3´ |
| Prdm14-F  Prdm14-R | 5´- GCCTGAACAAGCACATGAGA -3´  5´- AGGAAGCCTTTCCCACAAAT -3´ |
| Sox2-F  Sox2-R | 5‘- GAACGTTCATGGTATGGT-3‘  5‘- TTGCTGATCTCCGAGTTGT- 3‘ |
| Stella-F  Stella-R | 5´- GTCGGTGCTGAAAGACCCTA -3´  5´- GATTTCCCAGCACCAGAAAA -3´ |
| Tfap2c F  Tfap2c R | 5´- ATTTCGATGACCAAGAACCCTTTG -3´  5´- ACGAGAGACGTGAGGAGAGTGAC -3´ |
