# Supplementary material for: Transcription Factor TFAP2C Regulates Major Programs Required for Murine Fetal Germ Cell Maintenance and Haploinsufficiency Predisposes to Teratomas in Male Mice
Source: PLoS One. 2013 Aug 13;8(8):e71113. doi: 10.1371/journal.pone.0071113 (PMC3742748; doi:10.1371/journal.pone.0071113)
Supplement: Table S6 — Primer sequences for qRT-PCR. (DOCX) [file pone.0071113.s008.docx]

**Supplement Table S6: qRT-PCR Primer murin**

| bActin-F  bActin-R | 5´- TGTTACCAACTGGGACGACA -3´  5´- GGGGTGTTGAAGGTCTCAAA -3´ |
| --- | --- |
| Ccnd1-F  Ccnd1-R | 5´- tctttccagagtcatcaagtgtg -3´  5´- tcctcctcagtggccttg -3´ |
| c-Kit-F  c-Kit-R | 5´- gacgctgaccccttgaaa -3´  5´- gacttgggtttctgctcagg -3´ |
| Dazl-F  Dazl-R | 5´- tgctggagagcagaggagtt -3´  5´- tgaacattcattgggcaaaa -3´ |
| Dmrt1-F  Dmrt1-R | 5´- ctgatggccgagaacagc -3´  5´- ttctccatatgccctctgct -3´ |
| Dnmt3b-F  Dnmt3b-R | 5´- TGCGTCGTTCAGACAGTAGG -3´  5´- GCCCTTGTTGTTGGTGACTT -3´ |
| Dnmt3l-F  Dnmt3l-R | 5´- tctcacggagtggactgct -3´  5´- actggctgtctcttccatgc -3´ |
| Fgf4-F  Fgf4-R | 5´- gcaagctcttcggtgtgc -3´  5´- cgtaggattcgtaggcgttg -3´ |
| Gapdh-F  Gapdh-R | 5‘- GGTGCTGAGTATGTCGTGGA- 3‘  5‘- TTGGCTCCACCCTTCAAGT- 3‘ |
| Klf4-F  Klf4-R | 5´- gcgagtctgacatggctgt -3´  5´- gagttcctcacgccaacg -3´ |
| Nanog-F  Nanog-R | 5‘- GGTGGCAGAAAAACCAGTG- 3‘  5‘- GCAATGGATGCTGGGATACT- 3‘ |
| Nanos3-F  Nanos3-R | 5´- GCCAGGGCTACACTTCTGTC -3´  5´- CTTCCTGCCACTTTTGGAAC -3´ |
| p21-F  p21-R | 5´- TCTGAGCGGCCTGAAGATT -3´  5´- TGCGCTTGGAGTGATAGAAA -3´ |
| Rhox5-F  Rhox5-R | 5´- gatggggacaaggatagtgg -3´  5´- cagtgccctcagcaactg -3´ |
| Sox2-F  Sox2-R | 5‘- GAACGTTCATGGTATGGT-3‘  5‘- TTGCTGATCTCCGAGTTGT- 3‘ |
| Stella-F  Stella-R | 5´- CTTTGTTGTCGGTGCTGAAA -3´  5´- TCCCGTTCAAACTCATTTCC -3´ |
| Tfap2c-F  Tfap2c-R | 5´- AAGCGGTGGCTGACTATTTAA -3´  5´- CAGGCTGAAATGAGACAAACAG -3´ |

**human qRT-PCR Primer**

| hCCND1-F  hCCND1-R | 5´- tcacacgcttcctctccag -3´  5´- tggggtccatgttctgct -3´ |
| --- | --- |
| hDNMT3L-F  hDNMT3L-R | 5´- agcaactgggtgtgctacct -3´  5´- cgaacatctcaaggggattc -3´ |
| hDMRT1-F  hDMRT1-R | 5´- caagaagtgcaacctgatcg -3´  5´- tctctcttttgacaagcagctc -3´ |
| hDNMT3B-F  hDNMT3B-R | 5´- CCAGCTCTTACCTTACCATC -3´  5´- CAGACATAGCCTGTCGCTTG -3´ |
| hGAPDH-F  hGAPDH-R | 5´- TGCCAAATATGATGACATCAAGAA -3´  5´- GGAGTGGGTGTCGCTGTTG -3´ |
| hHOXA5-F  hHOXA5-R | 5´- GAACTCCTTCTCCAGCTCCA -3´  5´- GCGCAAGCTGCACATAAGTC -3´ |
| hC-KIT-F  hC-KIT-R | 5´- gggatgcagatcccctaaa -3´  5´- gacagaattgatccgcacag -3´ |
| hTFAP2C-F  hTFAP2C-R | 5´- GGCCCAGCAACTGTGTAAAGA -3´  5´- GCAGTTCTGTATGTTCGTCTCCAA -3´ |
