# Supplementary material for: Transcription Factor TFAP2C Regulates Major Programs Required for Murine Fetal Germ Cell Maintenance and Haploinsufficiency Predisposes to Teratomas in Male Mice
Source: PLoS One. 2013 Aug 13;8(8):e71113. doi: 10.1371/journal.pone.0071113 (PMC3742748; doi:10.1371/journal.pone.0071113)
Supplement: Table S7 — List of abbreviations. (DOCX) [file pone.0071113.s009.docx]

**Supplement-Table S7: List of abbreviations**

| **AP-2** | Activator protein-2 |
| --- | --- |
| **Bmp4** | Bone morphogenetic protein 4 |
| **Bmp8b** | Bone morphogenetic protein 8b |
| **Ccnd1** | Cyclin D1 |
| **Cdk6** | Cyclin-dependent kinase 6 |
| **Cdkn1a / p21** | Cyclin-dependent kinase inhibitor 1A (p21) |
| **cDNA** | Complementary DNA |
| **ChIP** | Chromatin-immunoprecipitation |
| **CIS** | Carcinoma in situ |
| **Ctrl** | Control |
| **Cxcr4** | Chemokine (C-X-C motif) receptor 4 |
| **Dazl** | Deleted in azoospermia-like |
| **DMEM** | Dulbecco’s modified eagle medium |
| **Dmrt1** | Doublesex and mab-3 related transcription factor 1 |
| **Dnmt3b** | DNA (cytosine-5-)-methyltransferase 3 beta |
| **Dnmt3l** | DNA (cytosine-5-)-methyltransferase 3-like |
| **E** | Embryonic day |
| **EB** | Embryoid body |
| **EC** | Embryonal carcinoma |
| **Egf** | Epidermal growth factor |
| **EpiLC** | Epiblast like cell |
| **Eras** | ES cell expressed Ras |
| **ESC** | Embryonic stem cell |
| **FACS** | Fluorescence activated cell sorting |
| **FCS** | - Fetal calf serum |
| **Fgf4** | Fibroblast growth factor 4 |
| **FoxD3** | Forkhead box D3 |
| **GCC** | Germ cell cancer |
| **GO** | Gene ontology |
| **Hoxa5** | Homeobox A5 |
| **IgG** | Immunoglobulin G |
| **Imm2lp** | IMP2 inner mitochondrial membrane peptidase-like |
| **Jam2** | Junction adhesion molecule 2 |
| **Klf4** | Kruppel-like factor 4 |
| **Lif** | Leukemia inhibitory factor |
| **mRNA** | Messenger RNA |
| **Nanos3** | Nanos homolog 3 (Drosophila) |
| **Oct3/4** | Octamer-binding protein 3/4 |
| **PGC** | Primordial germ cell |
| **PGCLC** | Primordial germ cells-like cell |
| **Prdm1/Blimp1** | PR domain zinc finger protein 1 / B lymphocyte-induced maturation protein 1 |
| **Prdm14** | PR domain zinc finger protein 14 |
| **qPCR** | Quantitative polymerase chain reaction |
| **qRT-PCR** | Quantitative reverse transcriptase polymerase chain reaction |
| **Rhox4a** | Reproductive homeobox 4a |
| **Rhox5** | Reproductive homeobox 5 |
| **Rhox6** | Reproductive homeobox 6 |
| **Rhox9** | Reproductive homeobox 9 |
| **Scf** | Stem cell factor |
| **SDS** | Sodium dodecyl sulfate |
| **Sfrp1** | Secreted frizzled-related protein 1 |
| **siRNA** | Short interference ribonucleic acid |
| **Sox2** | SRY (sex determining region Y)-box containing gene 2 |
| **Ssea1** | Stage-specific embryonic antigen 1 |
| **Stella / Dppa3** | Developmental pluripotency associated 3 |
| **Tdgf1** | Teratocarcinoma-derived growth factor 1 |
| **TSS** | Transcription start site |
| **WT** | Wildtype |
