# Supplementary material for: Transcription Factor TFAP2C Regulates Major Programs Required for Murine Fetal Germ Cell Maintenance and Haploinsufficiency Predisposes to Teratomas in Male Mice
Source: PLoS One. 2013 Aug 13;8(8):e71113. doi: 10.1371/journal.pone.0071113 (PMC3742748; doi:10.1371/journal.pone.0071113)
Supplement: Data S1 — Gene Ontology analysis for >1.5 fold-change in log2 scale for Tfap2c−/−PGCLCs in comparison to ctrl PGCLCs. (PDF) [file pone.0071113.s010.pdf]

Supplemental Data S1: Gene Ontology analysis for 1.5 fold change in log2 scale for Tfap2c$^{-/-}$ (KO) PGCLCs in comparison to ctrl PGCLCs.

1 Introduction

List of samples:

1. #1 / #2 **ESC Ctrl**,
2. #1 / #2 **ESC KO**
3. #1 / #2 **PGC Ctrl**
4. #1 / #2 **PGC KO**
5. **ESC**, arrays: ESC-1, ESC-2, ESC-3 as control

2 Differentially expressed genes between PGC KO and PGC CTRL

2.1 Up-regulated in PGC KO in relation to PGC CTRL

Number of probes fulfilling the condition: 247.

In the following figures, the expression of the 247 probes is distributed into 3 heat maps. Each one with 83 probes. The last one with 81 probes.

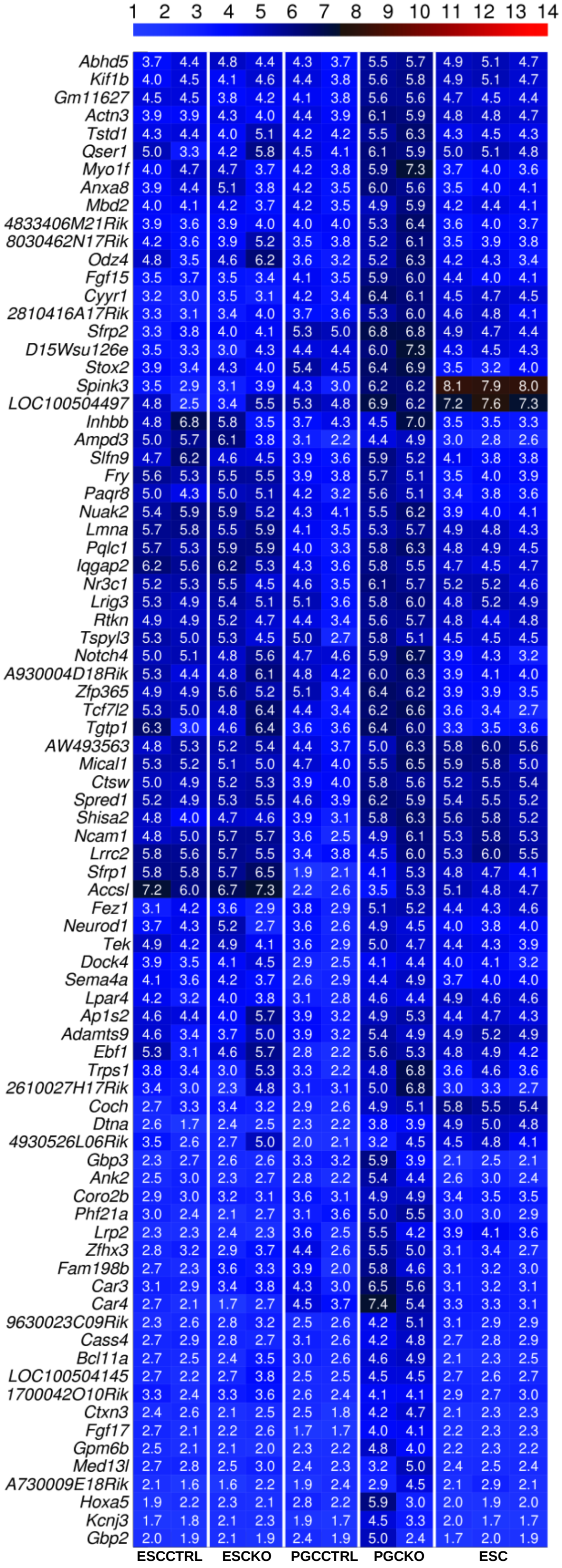

Figure 1: Heat map number 1 (83 probes of 247). PGC KO->-PGC CTRL-Log2(2.8284).

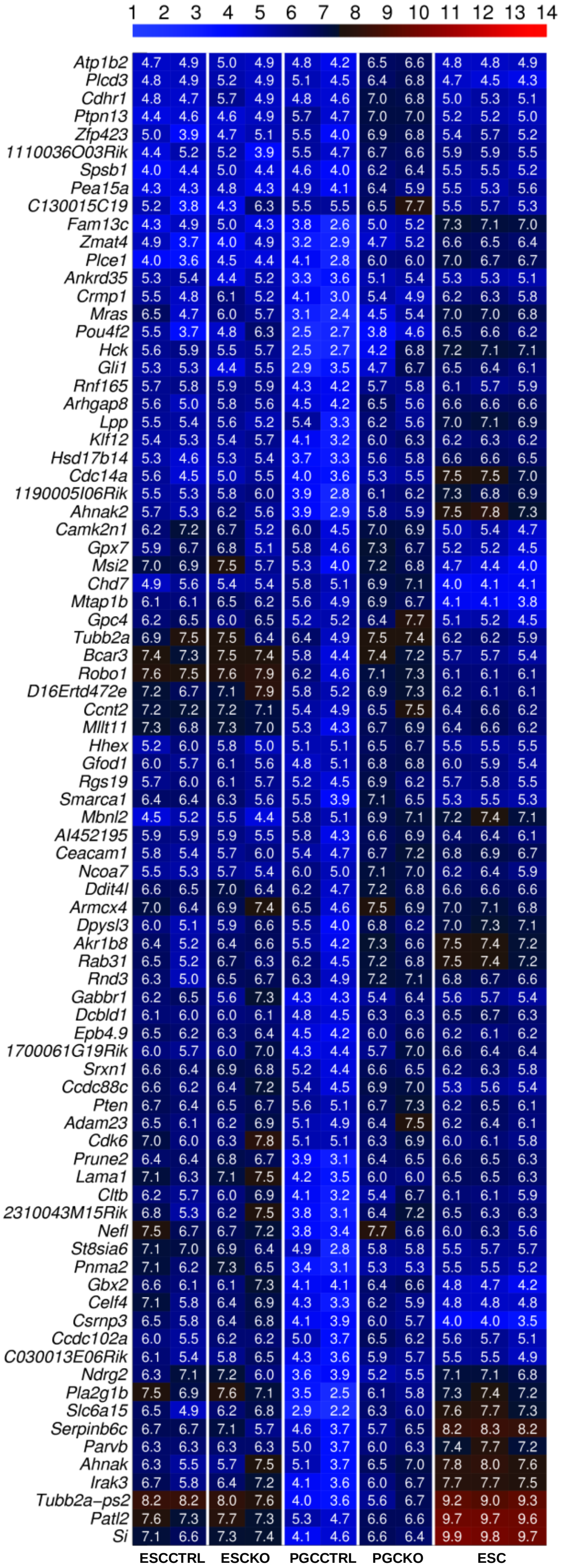

Figure 2: Heat map number 2 (83 probes of 247). PGC KO->-PGC CTRL-Log2(2.8284).

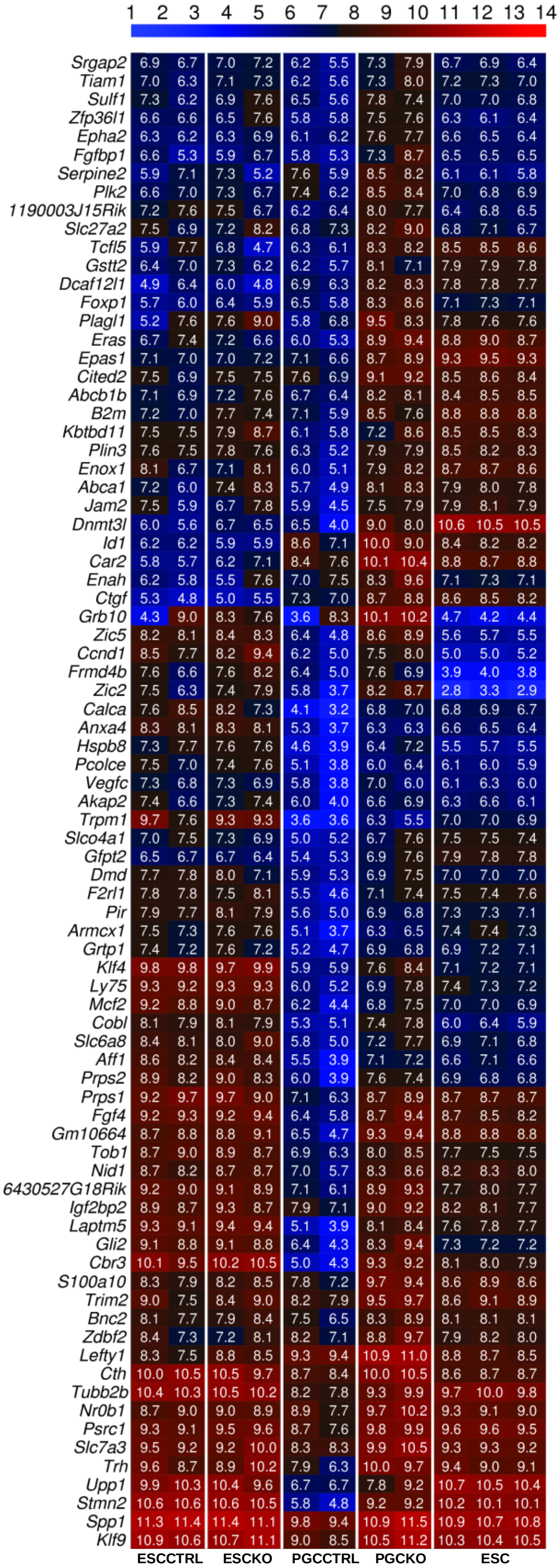

Figure 3: Heat map number 3 (81 probes of 247). PGC KO->-PGC CTRL-Log2(2.8284).

2.2 GO enrichment analysis of PGC KO->-PGC CTRL-Log2(2.8284)

Number of probes in the signal set 247
Number of unique probes in the signal set 247
Number of probes in the background set 45101
Number of unique probes in the background set 21390

Table 1: GO terms of PGC KO->-PGC CTRL-Log2(2.8284)-molecular-function

GO	Ter
GO:0000166	nucleotide binding (3.1%)
GO:0003824	catalytic activity (3.1%)
GO:0017076	purine nucleotide binding (8.4%)
GO:0005515	protein binding (9.6%)
GO:0005488	binding (9.8%)
GO:0043167	ion binding (11%)
Others	Others (55%)

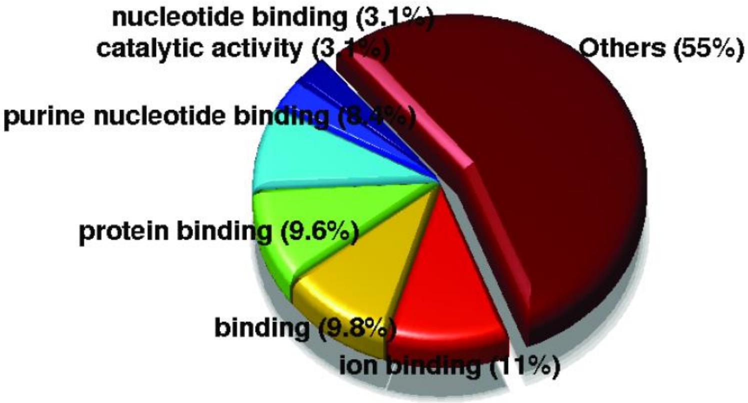

Figure 4: Pie graph of PGC KO->-PGC CTRL-Log2(2.8284)-molecular-function

Table : GO terms of PGC KO->-PGC CTRL-Log2(2.8284)-biological-process

GO	Ter
GO:0050789	regulation of biological process (3.1%)
GO:0023051	regulation of signaling (3.4%)
GO:0009889	regulation of biosynthetic process (3.4%)
GO:0050793	regulation of developmental process (3.6%)
GO:0016043	cellular component organization (4%)
GO:0060255	regulation of macromolecule metabolic process (4.1%)
GO:0050896	response to stimulus (5.6%)
GO:0032502	developmental process (9.4%)
GO:0050794	regulation of cellular process (9.6%)
Others	Others (54%)

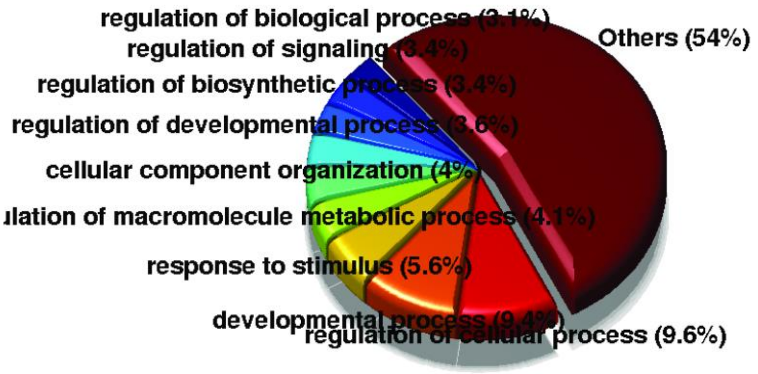

Figure 5: Pie graph of PGC KO->-PGC CTRL-Log2(2.8284)-biological-process

Table : GO terms of PGC KO->-PGC CTRL-Log2(2.8284)-cellular-component

GO	Ter
GO:0044459	plasma membrane part (3.1%)
GO:0044422	organelle part (3.2%)
GO:0043232	intracellular non-membrane-bounded organelle (3.3%)
GO:0044425	membrane part (3.4%)
GO:0042995	cell projection (3.6%)
GO:0043234	protein complex (4%)
GO:0043227	membrane-bounded organelle (4.9%)
GO:0044444	cytoplasmic part (5.5%)
GO:0043229	intracellular organelle (5.7%)
GO:0043226	organelle (5.7%)
GO:0031224	intrinsic to membrane (5.9%)
GO:0016020	membrane (6.7%)
GO:0043231	intracellular membrane-bounded organelle (11%)
GO:0044424	intracellular part (11%)
Others	Others (23%)

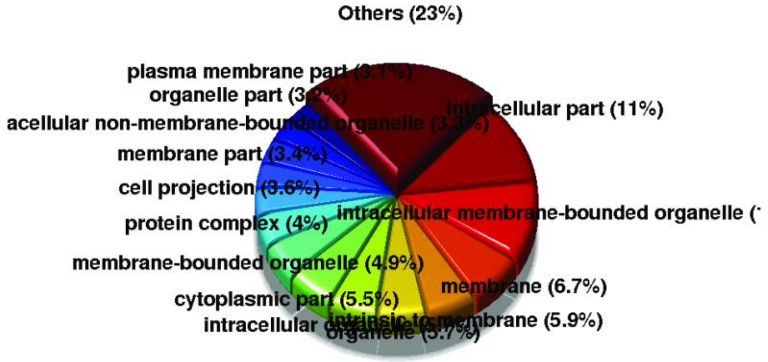

Figure 6: Pie graph of PGC KO->-PGC CTRL-Log2(2.8284)-cellular-component

Molecular-function-PGC KO->-PGC CTRL-Log2(2.8284)

Table : Significant GO enrichment terms for Molecular-function-PGC KO->-PGC CTRL-Log2(2.8284)

Count Ratio	*p*-val	GO id	GO term	Genes
11/211	0.00037468	GO:0003682	chromatin binding	Chd7 Foxp1 Gli1 Gli2 Hhex Mbd2 Nr3c1 Phf21a Pou4f2 Smarca1 Trps1

10/202	0.00097897	GO:0016564 transcription repressor activity	Cited2 Dnmt3l Foxp1 Hhex Id1 Klf4 Nr0b1 Trps1 Zfhx3 Zfp423
14/375	0.0020397	GO:0019001 guanyl nucleotide binding	Dock4 Eras Gbp2 Gbp3 Mras Prps1 Prps2 Rab31 Rnd3 Rtkn Tgtp1 Tubb2a Tubb2a-ps2 Tubb2b
16/458	0.0021479	GO:0019904 protein domain specific binding	Akap2 Ccdc88c Cited2 Dmd Dock4 Dpysl3 Ebf1 Enah Lrp2 Mbd2 Mical1 Ncam1 Nefl Nr0b1 Pten Robo1
6/102	0.0036182	GO:0015631 tubulin binding	Ccdc88c Fez1 Gli1 Kif1b Mtap1b Psrc1
14/400	0.0037731	GO:0030528 transcription regulator activity	Cited2 Ebf1 Epas1 Gbx2 Hhex Hoxa5 Id1 Klf9 Neurod1 Nr3c1 Pou4f2 Smarca1 Tcfl5 Zfhx3
8/171	0.0041485	GO:0003924 GTPase activity	Eras Gbp2 Gbp3 Mras Tgtp1 Tubb2a Tubb2a-ps2 Tubb2b
7/138	0.0044018	GO:0008083 growth factor activity	Ctgf Fgf15 Fgf17 Fgf4 Inhbb Lefty1 Vegfc
5/77	0.00484	GO:0008017 microtubule binding	Ccdc88c Gli1 Kif1b Mtap1b Psrc1
22/774	0.0055559	GO:0001071 nucleic acid binding transcription factor activity	Aff1 Cited2 Csrnp3 Ebf1 Epas1 Foxp1 Gbx2 Gli1 Gli2 Hhex Hoxa5 Id1 Klf12 Klf4 Neurod1 Nr0b1 Nr3c1 Pou4f2 Tcf7l2 Trps1 Zfhx3 Zfp423
5/85	0.0075837	GO:0070851 growth factor receptor binding	Fgf15 Fgf17 Fgf4 Pten Vegfc
12/360	0.010293	GO:0005525 GTP binding	Dock4 Eras Gbp2 Gbp3 Mras Rab31 Rnd3 Rtkn Tgtp1 Tubb2a Tubb2a-ps2 Tubb2b
16/569	0.017403	GO:0043565 sequence-specific DNA binding	Epas1 Foxp1 Gbx2 Gli2 Hhex Hoxa5 Klf4 Mbd2 Neurod1 Nr0b1 Nr3c1 Pou4f2 Tcf7l2 Trps1 Zfhx3 Zfp423
5/106	0.019251	GO:0017124 SH3 domain binding	Dock4 Dpysl3 Enah Lrp2 Mical1
22/878	0.022505	GO:0005102 receptor binding	Abca1 Calca Ctgf Dmd Fgf15 Fgf17 Fgf4 Grb10 Inhbb Lama1 Lefty1 Nr0b1 Nr3c1 Pla2g1b Pten Serpine2 Spp1 Spred1 Tcf7l2 Tiam1 Trh Vegfc
14/501	0.026259	GO:0008092 cytoskeletal protein binding	Actn3 Ccdc88c Coro2b Dmd Enah Epb4.9 Fez1 Gli1 Kif1b Mtap1b Myo1f Parvb Psrc1 Trim2
11/367	0.028403	GO:0030695 GTPase regulator activity	Arhgap8 Bcar3 Dock4 Grtp1 Iqgap2 Mcf2 Plce1 Rgs19 Rtkn Srgap2 Tiam1
4/82	0.030322	GO:0003729 mRNA binding	Celf4 Igf2bp2 Mbd2 Zfp36l1
4/82	0.030631	GO:0016298 lipase activity	Abhd5 Pla2g1b Plcd3 Plce1
19/766	0.036637	GO:0030234 enzyme regulator activity	Arhgap8 Bcar3 Camk2n1 Ccnd1 Dnmt3l Dock4 Grtp1 Iqgap2 Klf4 Mcf2 Plce1 Rgs19 Rtkn Serpinb6c Serpine2 Sfrp2 Spink3 Srgap2 Tiam1
16/633	0.044822	GO:0019899 enzyme binding	Abca1 Camk2n1 Ccnd1 Ceacam1 Dmd Dnmt3l Dock4 Epas1 Fez1 Ncam1 Nefl Pea15a Plce1 Rtkn Tcf7l2 Zfhx3

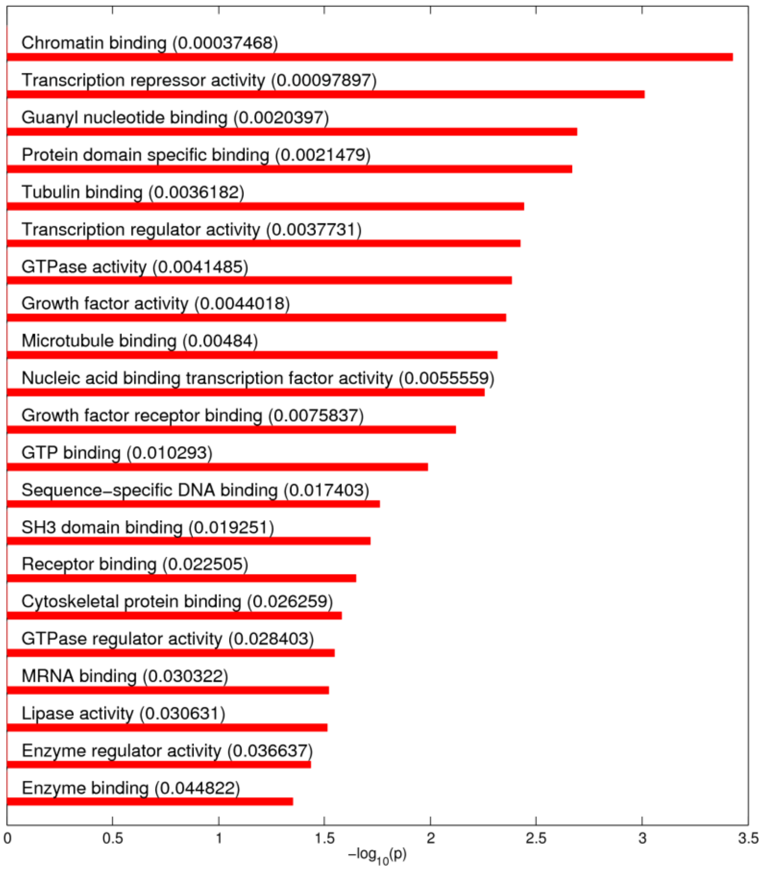

Figure 7: Plot bar of the -log10(p) of the significant enriched terms of Molecular-function-PGC KO->-PGC CTRL-Log2(2.8284). The longer the bar, the higher is the statistical significance of the enrichment (in parenthesis are written the p-values)

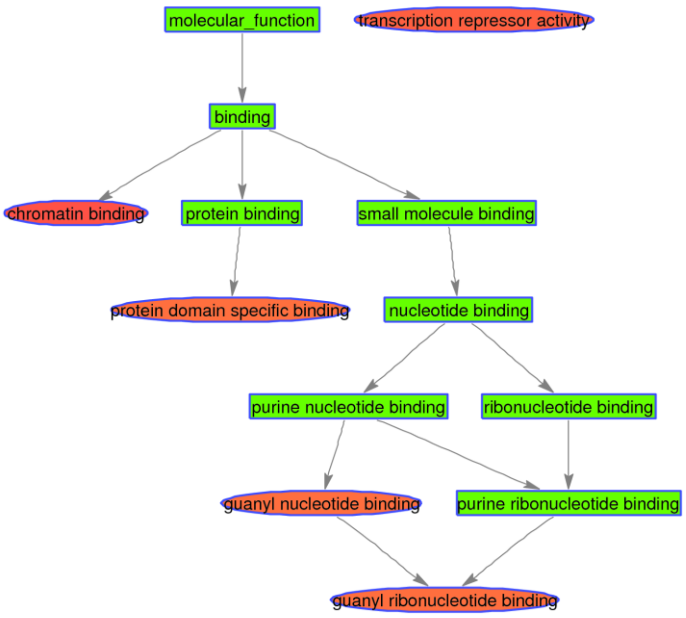

Figure 8: Direct acyclic graph (DAG) associated to the 5 top higher significant enriched gene ontology terms. The higher the significance of the term the more red is the box framing the term. The significant enriched terms are encircled by ellipses.

Biological-process-PGC KO->-PGC CTRL-Log2(2.8284)

Table : Significant GO enrichment terms for Biological-process-PGC KO->-PGC CTRL-Log2(2.8284)

**Count Ratio**	***p*-val**	**GO id**	**GO term**	**Genes**
37/923	7.1306e□05	GO:0050793	regulation of developmental process	Adamts9 Akap2 Car2 Ccnd1 Cdk6 Dpysl3 Epha2 Fez1 Fgf4 Gli2 Hhex Hoxa5 Id1 Irak3 Klf4 Lama1 Lmna Mtap1b Ncam1 Nefl Neurod1 Notch4 Nr0b1 Pou4f2 Pten Robo1 Serpine2 Sfrp1 Sfrp2 Spp1 Tcf7l2 Tcfl5 Tek Tiam1 Tob1 Vegfc Zfhx3
5/30	0.00017221	GO:0021915	neural tube development	Epha2 Gli2 Sfrp1 Sfrp2 Tcf7l2
28/659	0.0001846	GO:0045595	regulation of cell differentiation	Adamts9 Car2 Ccnd1 Cdk6 Dpysl3 Fez1 Fgf4 Gli2 Hoxa5 Klf4 Mtap1b Nefl Neurod1 Notch4 Nr0b1 Pou4f2 Pten Robo1 Serpine2 Sfrp1 Sfrp2 Spp1 Tcf7l2 Tcfl5 Tiam1 Tob1 Vegfc Zfhx3
9/110	0.0002545	GO:0033273	response to vitamin	Abca1 Abcb1b Ccnd1 Klf4 Lefty1 Lrp2 Mtap1b Spp1 Tek
6/49	0.00025791	GO:0001843	neural tube closure	Cobl Enah Sfrp1 Sfrp2 Zic2 Zic5
5/33	0.0002774	GO:0060562	epithelial tube morphogenesis	Gli2 Hhex Hoxa5 Lama1 Nr3c1
32/817	0.00030446	GO:0048513	organ development	Anxa4 Car2 Car4 Ccnd1 Cdk6 Chd7 Cited2 Ctgf Dmd Epas1 Fgf15 Foxp1 Gli1 Gli2 Hhex Hoxa5 Id1 Inhbb Klf4 Lmna Neurod1 Notch4 Nr0b1 Nr3c1 Plcd3 Pten Sfrp1 Sfrp2 Smarca1 Tek Trps1 Zfhx3
42/1196	0.00043351	GO:0030154	cell differentiation	Abhd5 Bcl11a Ccnd1 Cdk6 Chd7 Cited2 Ctgf Enah Epas1 Epha2 Fgf4 Foxp1 Gabbr1 Gli1 Gli2 Hhex Hoxa5 Inhbb Klf4 Lama1 Ndrg2 Neurod1 Notch4 Nr0b1 Paqr8 Pou4f2 Robo1 Sema4a Serpine2 Sfrp1 Sfrp2 Smarca1 Spp1 Tcf7l2 Tubb2a Tubb2a-ps2 Tubb2b Vegfc Zfp36l1 Zfp423 Zic2 Zic5
6/54	0.00044702	GO:0007417	central nervous system development	Chd7 Cited2 Mtap1b Pten Robo1 Zic2

12/196	0.00047635	GO:0002009	morphogenesis of an epithelium	Car2 Cited2 Gbx2 Gli2 Hhex Hoxa5 Lama1 Notch4 Nr3c1 Sfrp1 Sfrp2 Vegfc
4/23	0.00060057	GO:0010165	response to X-ray	Ccnd1 Lrp2 Sfrp1 Sfrp2
57/1799	0.00067964	GO:0048856	anatomical structure development	Abcb1b Anxa4 Car2 Car4 Ccnd1 Cdk6 Chd7 Cited2 Ctgf Dmd Dnmt3l Dpysl3 Enah Epas1 Epha2 Fgf15 Foxp1 Gbx2 Gli1 Gli2 Hhex Hoxa5 Id1 Inhbb Kif1b Klf4 Lama1 Lmna Lrp2 Mcf2 Msi2 Mtap1b Ncam1 Ndrg2 Neurod1 Notch4 Nr0b1 Nr3c1 Plcd3 Plce1 Pou4f2 Prps1 Pten Robo1 Sema4a Serpine2 Sfrp1 Sfrp2 Smarca1 Spp1 Tcf7l2 Tek Trps1 Zfhx3 Zfp423 Zic2 Zic5
4/24	0.00071465	GO:0051385	response to mineralocorticoid stimulus	Ccnd1 Ctgf Nefl Trh
51/1584	0.00087662	GO:0048869	cellular developmental process	Abcb1b Abhd5 Bcl11a Calca Ccnd1 Cdk6 Chd7 Cited2 Ctgf Dmd Enah Epas1 Epha2 Fgf4 Foxp1 Gabbr1 Gli1 Gli2 Hhex Hoxa5 Id1 Inhbb Klf4 Lama1 Lmna Msi2 Mtap1b Ncam1 Ndrg2 Nefl Neurod1 Notch4 Nr0b1 Paqr8 Pou4f2 Robo1 Sema4a Serpine2 Sfrp1 Sfrp2 Smarca1 Spp1 Tcf7l2 Tubb2a Tubb2a-ps2 Tubb2b Vegfc Zfp36l1 Zfp423 Zic2 Zic5
34/955	0.0011004	GO:0009966	regulation of signal transduction	Ccnd1 Ceacam1 Cited2 Ddit4l Fgf15 Fgf4 Fgfbp1 Gli2 Grb10 Hhex Id1 Iqgap2 Irak3 Klf4 Mbd2 Mcf2 Ncam1 Nr0b1 Nr3c1 Plce1 Plk2 Pten Rgs19 Robo1 Serpine2 Sfrp1 Sfrp2 Slc7a3 Spred1 Tcf7l2 Tek Tiam1 Tob1 Vegfc
8/109	0.0011069	GO:0048732	gland development	Cited2 Gli1 Gli2 Hhex Hoxa5 Notch4 Nr0b1 Nr3c1
15/310	0.0013376	GO:0009968	negative regulation of signal transduction	Ccnd1 Ddit4l Grb10 Hhex Irak3 Klf4 Nr0b1 Nr3c1 Pten Rgs19 Robo1 Serpine2 Sfrp1 Sfrp2 Tob1
14/280	0.0013692	GO:0045596	negative regulation of cell differentiation	Ccnd1 Cdk6 Fgf4 Gli2 Hoxa5 Klf4 Notch4 Nr0b1 Sfrp1 Sfrp2 Spp1 Tcf7l2 Tob1 Zfhx3
20/474	0.0014477	GO:0048731	system development	Chd7 Cited2 Dpysl3 Enah Epha2 Gbx2 Gli2 Hoxa5 Id1 Mtap1b Ndrg2 Neurod1 Pou4f2 Prps1 Pten Robo1 Sema4a Serpine2 Zfp423 Zic2
10/168	0.0016146	GO:0030155	regulation of cell adhesion	Calca Cdk6 Cited2 Lama1 Myo1f Nid1 Pten Serpine2 Spp1 Tek
6/69	0.0017155	GO:0033189	response to vitamin A	Abca1 Klf4 Lefty1 Lrp2 Mtap1b Tek
39/1170	0.001751	GO:0023051	regulation of signaling	Ccnd1 Ceacam1 Chd7 Cited2 Ddit4l Fgf15 Fgf4 Fgfbp1 Gabbr1 Gli2 Grb10 Hhex Id1 Inhbb Iqgap2 Irak3 Klf4 Mbd2 Mcf2 Mtap1b Ncam1 Nr0b1 Nr3c1 Plce1 Plk2 Pten Rgs19 Robo1 Serpine2 Sfrp1 Sfrp2 Slc7a3 Spred1 Tcf7l2 Tek Tiam1 Tob1 Trh Vegfc
7/93	0.0018497	GO:0031346	positive regulation of cell projection organization	Dpysl3 Fez1 Mtap1b Nefl Robo1 Serpine2 Tiam1
9/144	0.0018771	GO:0010975	regulation of neuron projection development	Dpysl3 Fez1 Mtap1b Nefl Pten Robo1 Serpine2 Spp1 Tiam1
4/31	0.0019997	GO:0051896	regulation of protein kinase B signaling cascade	Klf4 Pten Tcf7l2 Tek
15/325	0.002167	GO:0045597	positive regulation of cell differentiation	Adamts9 Car2 Fez1 Gli2 Hoxa5 Mtap1b Nefl Neurod1 Pou4f2 Robo1 Sfrp1 Sfrp2 Tiam1 Vegfc Zfhx3
27/737	0.0022092	GO:2000026	regulation of multicellular organismal development	Car2 Ccnd1 Cdk6 Dpysl3 Epha2 Fez1 Gli2 Hhex Hoxa5 Id1 Irak3 Lama1 Mtap1b Ncam1 Nefl Neurod1 Notch4 Pten Robo1 Serpine2 Sfrp1 Sfrp2 Spp1 Tek Tiam1 Vegfc Zfhx3
4/32	0.0022711	GO:0008543	fibroblast growth factor receptor signaling pathway	Ctgf Fgf15 Fgf17 Fgf4
6/73	0.0023449	GO:0035239	tube morphogenesis	Gbx2 Gli2 Hhex Hoxa5 Lama1 Nr3c1
9/149	0.0024126	GO:0001763	morphogenesis of a branching structure	Cited2 Gbx2 Gli2 Hhex Hoxa5 Lama1 Notch4 Sfrp1 Sfrp2
5/52	0.0024908	GO:0071363	cellular response to growth factor stimulus	Ctgf Fez1 Fgf15 Fgf17 Fgf4
8/123	0.0025029	GO:004875	branching morphogenesis	Cited2 Gbx2 Gli2 Hhex Hoxa5 Lama1 Notch4 Sfrp2

		4	of a tube	
8/126	0.0029164	GO:006113 8	morphogenesis of a branching epithelium	Cited2 Gbx2 Hhex Hoxa5 Lama1 Notch4 Sfrp1 Sfrp2
18/434	0.0029366	GO:005109 4	positive regulation of developmental process	Adamts9 Car2 Fez1 Gli2 Hoxa5 Lmna Mtap1b Ncam1 Nefl Neurod1 Pou4f2 Robo1 Sfrp1 Sfrp2 Tek Tiam1 Vegfc Zfhx3

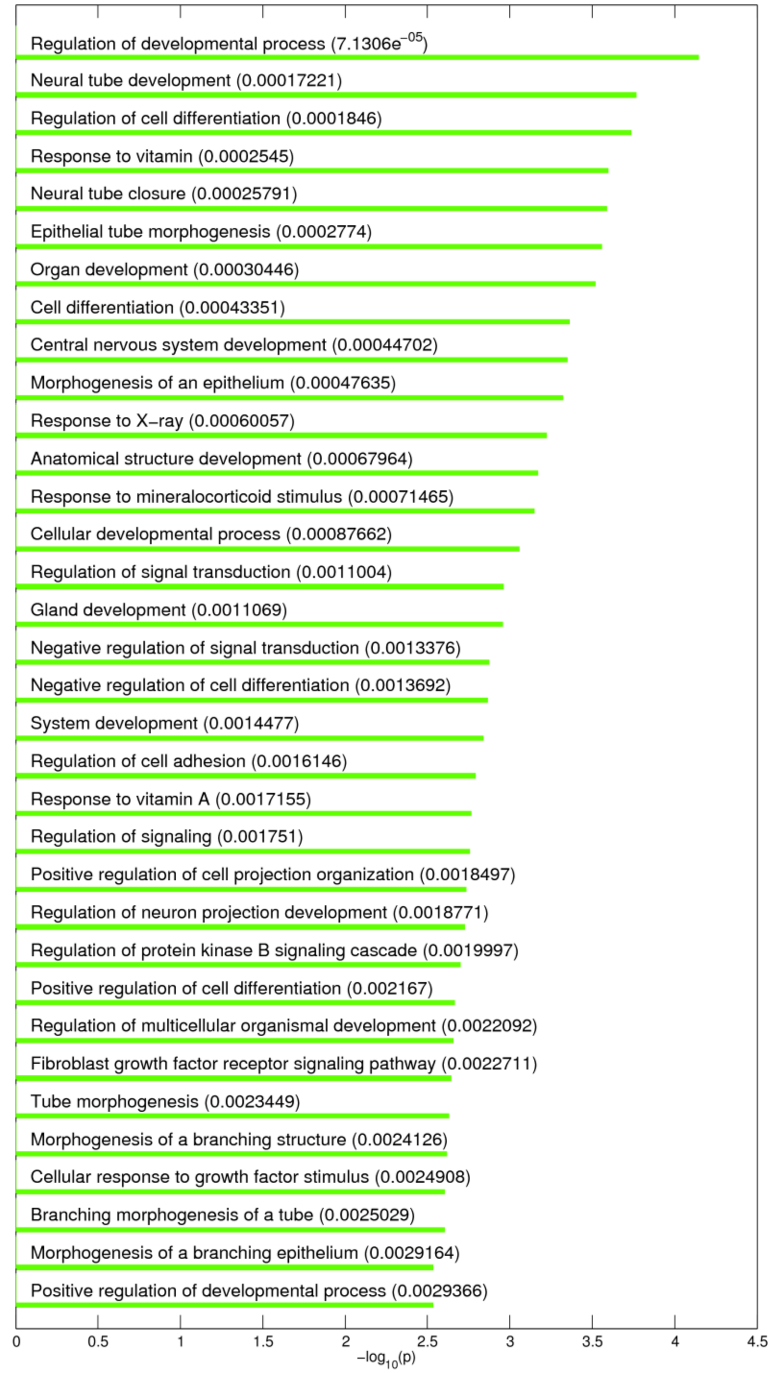

Figure 9: Plot bar of the -log10(p) of the significant enriched terms of Biological-process-PGC KO->-PGC CTRL-Log2(2.8284). The longer the bar, the higher is the statistical significance of the enrichment (in parenthesis are written the p-values)

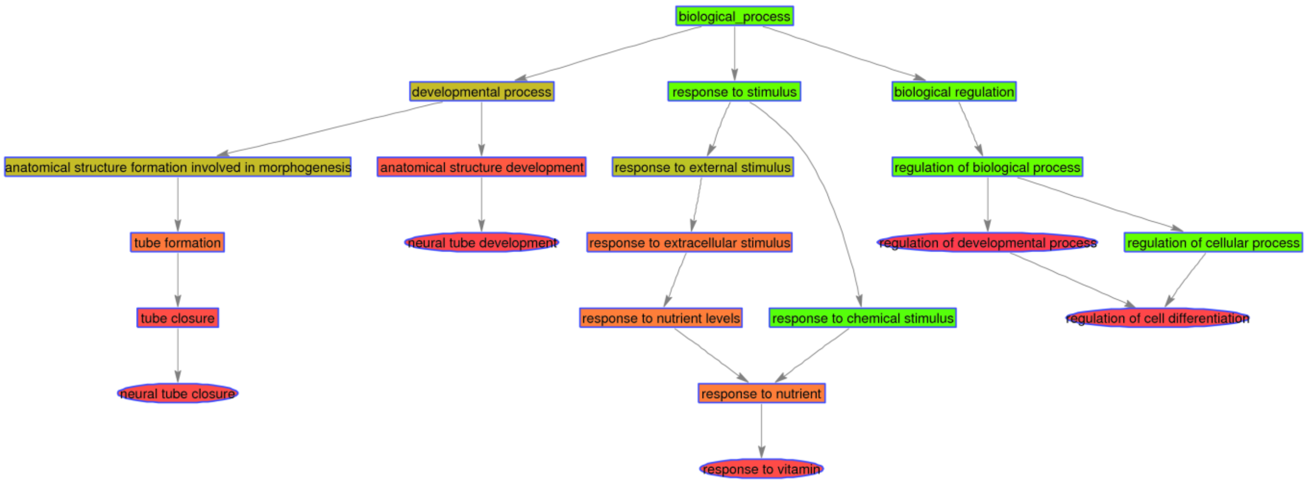

Figure 10: Direct acyclic graph (DAG) associated to the 5 top higher significant enriched gene ontology terms. The higher the significance of the term the more red is the box framing the term. The significant enriched terms are encircled by ellipses.

Cellular-component-PGC KO->-PGC CTRL-Log2(2.8284)

Table : Significant GO enrichment terms for Cellular-component-PGC KO->-PGC CTRL-Log2(2.8284)

**Count Ratio**	***p*-val**	**GO id**	**GO term**	**Genes**
24/838	0.00057365	GO:0042995	cell projection	Calca Camk2n1 Car2 Cdk6 Crmp1 Dock4 Dpysl3 Enah Fez1 Frmd4b Gabbr1 Gli1 Gli2 Lrp2 Mtap1b Ncam1 Nefl Pten Ptpn13 Rgs19 Robo1 Spp1 Stmn2 Tek
30/1203	0.0013145	GO:0044459	plasma membrane part	Abca1 Abcb1b Ank2 Anxa4 Atp1b2 B2m Car2 Car4 Cdhr1 Cltb Dmd Dtna Epha2 F2rl1 Gabbr1 Gpc4 Hck Kcnj3 Laptm5 Lrp2 Ly75 Ncam1 Robo1 S100a10 Slc6a15 Slc6a8 Slc7a3 Slco4a1 Spred1 Tek
19/655	0.0016804	GO:0005615	extracellular space	Adamts9 B2m Calca Car2 Coch Ctgf Dpysl3 Enox1 Fgfbp1 Gpc4 Lama1 Lefty1 Lrp2 Pla2g1b Serpine2 Sfrp2 Spp1 Sulf1 Vegfc
8/178	0.0022893	GO:0030424	axon	Calca Car2 Fez1 Mtap1b Ncam1 Nefl Robo1 Stmn2
26/1089	0.0046504	GO:0000267	cell fraction	Abca1 Camk2n1 Car4 Ccdc88c Dmd Dpysl3 Dtna Fgf4 Gabbr1 Gli2 Gpc4 Id1 Kif1b Lmna Lrp2 Mtap1b Nr0b1 Pea15a Plcd3 Plce1 Prps1 Prps2 Pten Rgs19 Serpine2 Slc27a2
12/382	0.0055512	GO:0031226	intrinsic to plasma membrane	Abca1 Ank2 Car4 Cdhr1 Epha2 F2rl1 Laptm5 Slc6a15 Slc6a8 Slc7a3 Slco4a1 Tek
5/94	0.006436	GO:0030055	cell-substrate junction	Cass4 Dmd Enah Lpp Parvb
13/460	0.010055	GO:0043005	neuron projection	Calca Camk2n1 Car2 Crmp1 Fez1 Gabbr1 Mtap1b Ncam1 Nefl Pten Ptpn13 Robo1 Stmn2
9/278	0.012253	GO:0044297	cell body	Calca Camk2n1 Crmp1 Gabbr1 Mtap1b Ncam1 Ptpn13 Robo1 Serpine2
18/736	0.013251	GO:0005829	cytosol	Abhd5 Car2 Cbr3 Ccnd1 Ctgf Dnmt3l Enah Gbp3 Gli1 Grb10 Gstt2 Mtap1b Nr3c1 Plce1 Psrc1 Srxn1 Tcf7l2 Zfp36l1
35/1705	0.013853	GO:0005622	intracellular	Arhgap8 Bcar3 Bcl11a Bnc2 Calca Ccnd1 Eras Foxp1 Gli1 Gli2 Grtp1 Hspb8 Iqgap2 Klf12 Klf4 Klf9 Mcf2 Mras Ncoa7 Nr3c1 Plagl1 Plce1 Rnd3 Rtkn Srgap2 Tgtp1 Tiam1 Trim2 Trps1 Zfhx3 Zfp365 Zfp423 Zic2 Zic5 Zmat4
20/851	0.014737	GO:0005626	insoluble fraction	Abca1 Camk2n1 Car4 Ccdc88c Dmd Dtna Gabbr1 Gli2 Gpc4 Kif1b Lmna Lrp2 Nr0b1 Pea15a Plcd3 Plce1 Pten Rgs19 Serpine2 Slc27a2
7/196	0.015525	GO:0016324	apical plasma membrane	Abcb1b Ank2 Anxa4 Atp1b2 Car4 Lrp2 Tek
4/84	0.021809	GO:0005925	focal adhesion	Cass4 Enah Lpp Parvb

8/270	0.030882	GO:0043025 neuronal cell body	Calca Camk2n1 Crmp1 Gabbr1 Mtap1b Ncam1 Robo1 Serpine2
5/140	0.037938	GO:0000785 chromatin	Chd7 Cited2 Dnmt3l Klf4 Mbd2
8/281	0.039203	GO:0009986 cell surface	Ceacam1 Dmd Fgfbp1 Kcnj3 Notch4 Robo1 Sulf1 Tek
6/192	0.046114	GO:0045121 membrane raft	Abca1 Dmd Gabbr1 Hck Spred1 Tek

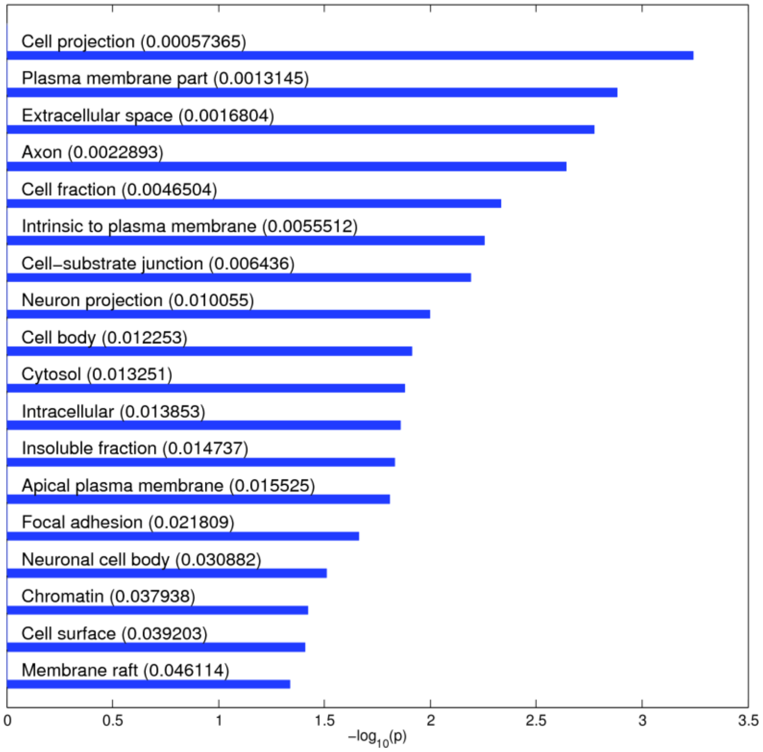

Figure 11: Plot bar of the -log10(p) of the significant enriched terms of Cellular-component-PGC KO->-PGC CTRL-Log2(2.8284). The longer the bar, the higher is the statistical significance of the enrichment (in parenthesis are written the p-values)

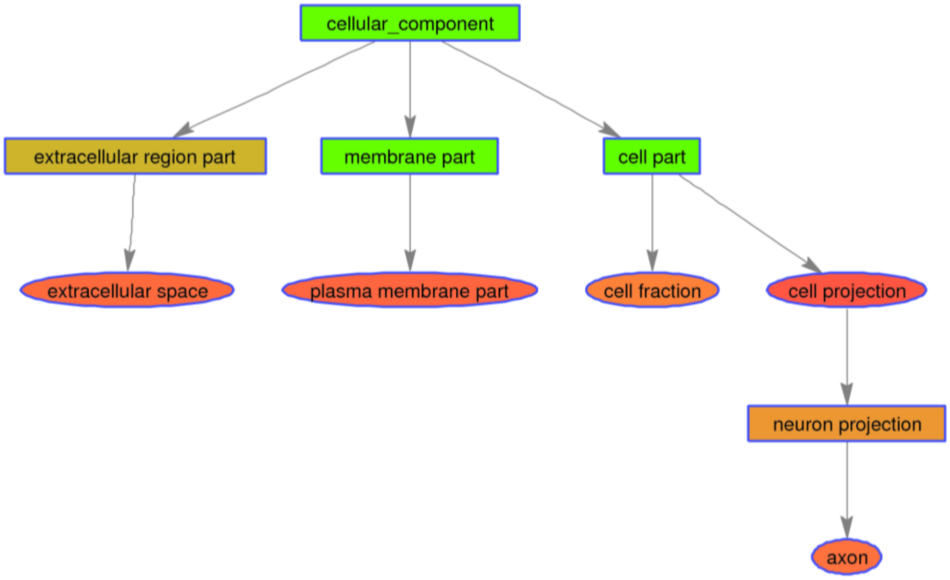

Figure 12: Direct acyclic graph (DAG) associated to the 5 top higher significant enriched gene ontology terms. The higher the significance of the term the more red is the box framing the term. The significant enriched terms are encircled by ellipses.

2.3 Down-regulated in PGC KO in relation to PGC CTRL

Number of probes fulfilling the condition: 208.

In the following figures, the expression of the 208 probes is distributed into 2 heat maps. Each one with 104 probes. The last one with 104 probes.

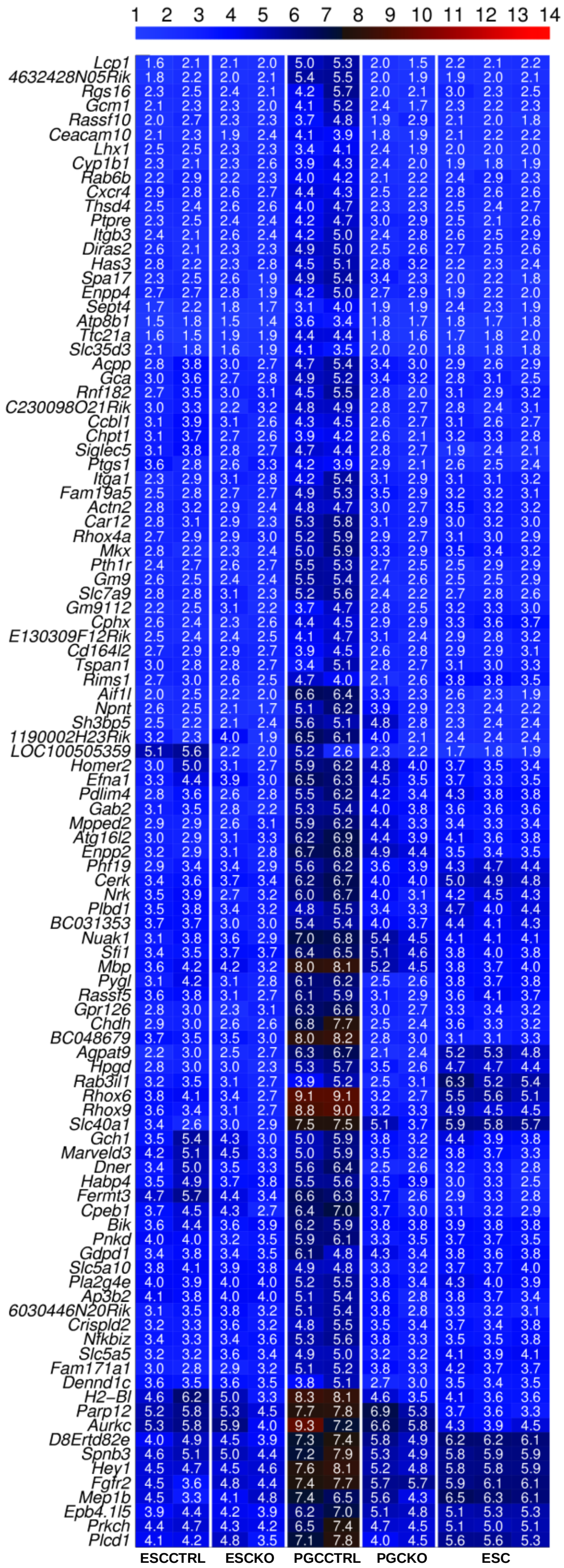

Figure : Heat map number 1 (104 probes of 208). PGC KO-<-PGC CTRL-Log2(2.8284).

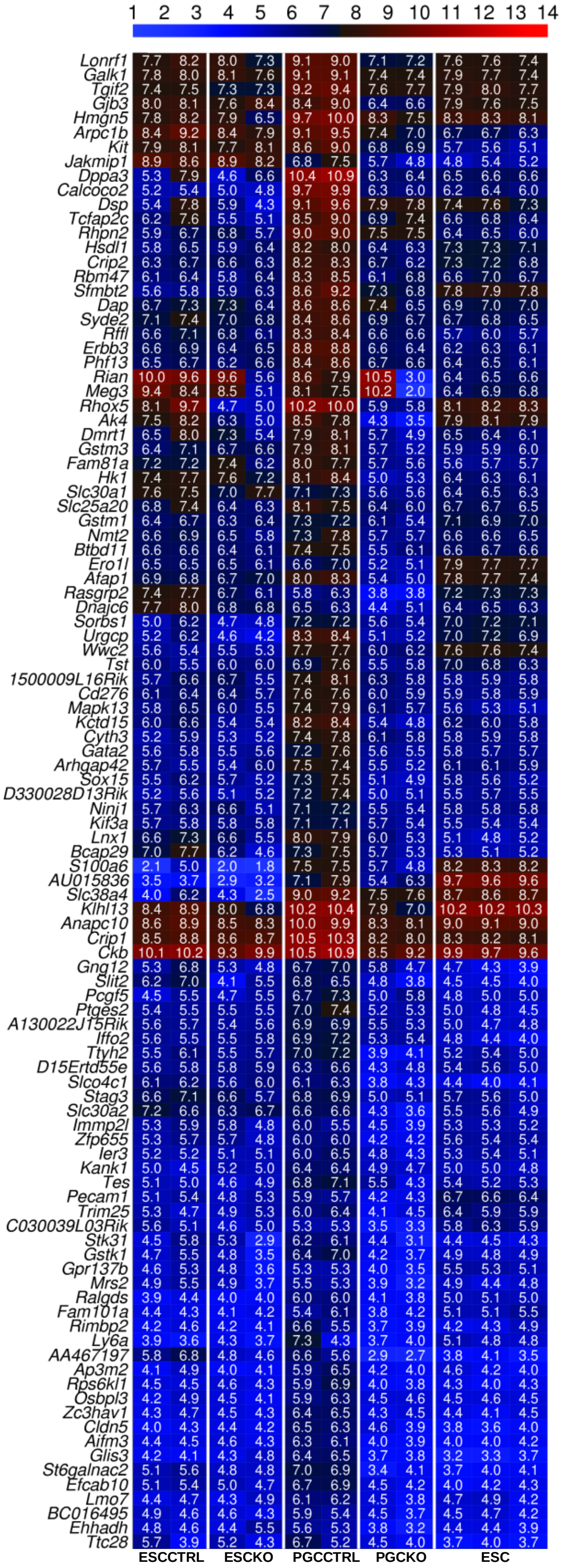

Figure : Heat map number 2 (104 probes of 208). PGC KO-<-PGC CTRL-Log2(2.8284).

2.4 GO enrichment analysis of PGC KO-<-PGC CTRL-Log2(2.8284)

Number of probes in the signal set 208
  Number of unique probes in the signal set 208
  Number of probes in the background set 45101
  Number of unique probes in the background set 21390

Table : GO terms of PGC KO-<-PGC CTRL-Log2(2.8284)-molecular-function

GO	Ter
GO:0000166	nucleotide binding (3%)
GO:0003824	catalytic activity (3.8%)
GO:0005515	protein binding (8.5%)
GO:0017076	purine nucleotide binding (8.5%)
GO:0005488	binding (11%)
GO:0043167	ion binding (13%)
Others	Others (53%)

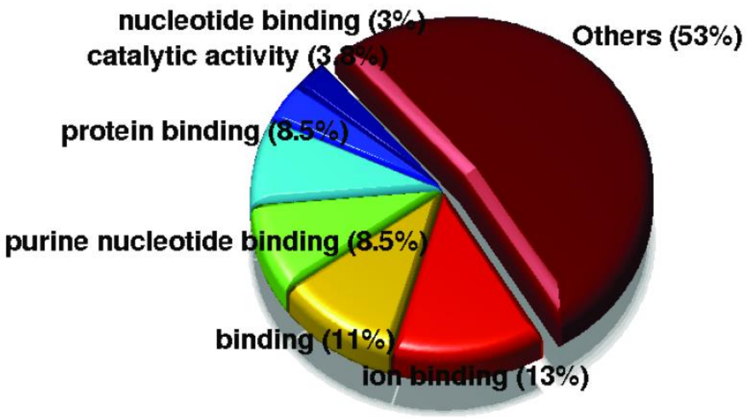

Figure : Pie graph of PGC KO-<-PGC CTRL-Log2(2.8284)-molecular-function

Table : GO terms of PGC KO-<-PGC CTRL-Log2(2.8284)-biological-process

GO	Ter
GO:0009889	regulation of biosynthetic process (3.1%)
GO:0060255	regulation of macromolecule metabolic process (3.2%)
GO:0031323	regulation of cellular metabolic process (3.2%)
GO:0050896	response to stimulus (3.4%)
GO:0016043	cellular component organization (3.6%)
GO:0050789	regulation of biological process (3.6%)
GO:0044237	cellular metabolic process (3.8%)
GO:0006810	transport (3.8%)
GO:0019538	protein metabolic process (3.9%)
GO:0032502	developmental process (8.1%)
GO:0050794	regulation of cellular process (9.7%)
Others	Others (51%)

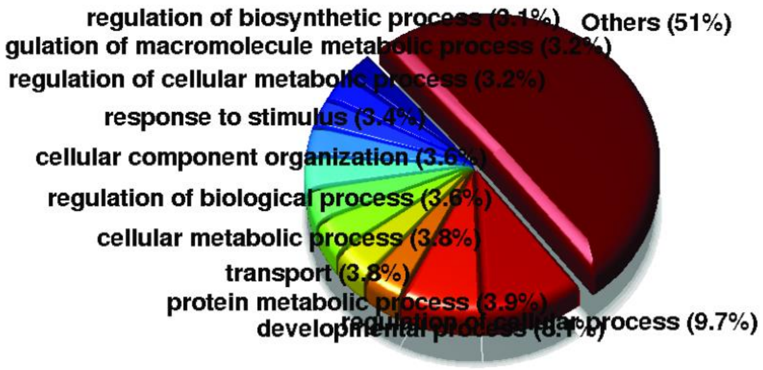

Figure : Pie graph of PGC KO-<-PGC CTRL-Log2(2.8284)-biological-process

Table : GO terms of PGC KO-<-PGC CTRL-Log2(2.8284)-cellular-component

GO	Ter
GO:0044446	intracellular organelle part (3.1%)
GO:0043234	protein complex (3.3%)
GO:0044422	organelle part (3.3%)
GO:0044459	plasma membrane part (3.3%)
GO:0030054	cell junction (3.3%)
GO:0031090	organelle membrane (3.5%)
GO:0044444	cytoplasmic part (4.5%)
GO:0043227	membrane-bounded organelle (4.9%)
GO:0044425	membrane part (4.9%)
GO:0043226	organelle (5.7%)
GO:0043229	intracellular organelle (5.7%)
GO:0031224	intrinsic to membrane (6.7%)
GO:0016020	membrane (8.1%)
GO:0044424	intracellular part (11%)
GO:0043231	intracellular membrane-bounded organelle (11%)
Others	Others (18%)

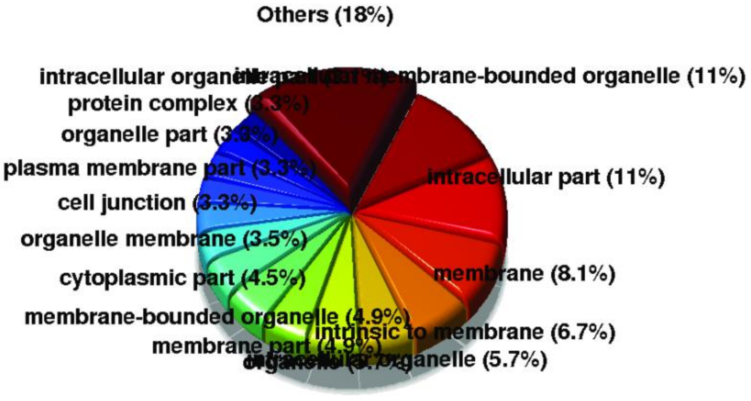

Figure : Pie graph of PGC KO-<-PGC CTRL-Log2(2.8284)-cellular-component

Molecular-function-PGC KO-<-PGC CTRL-Log2(2.8284)

Table : Significant GO enrichment terms for Molecular-function-PGC KO-<-PGC CTRL-Log2(2.8284)

Count Ratio	*p*-val	GO id	GO term	Genes
5/64	0.0007710	GO:0030674	protein binding, bridging	Actn2 Arhgap42 Dsp Gab2 Sorbs1

	7			
5/79	0.0020455	GO:0019887	protein kinase regulator activity	1190002H23Rik Efcab10 Erbb3 Sh3bp5 Spa17
4/54	0.0029211	GO:0005178	integrin binding	Fermt3 Itga1 Itgb3 Npnt
5/106	0.0075256	GO:0017124	SH3 domain binding	Afap1 Arhgap42 Dnajc6 Rims1 Sh3bp5
8/237	0.0082492	GO:0030246	carbohydrate binding	Crispld2 Enpp2 Fgfr2 Galk1 Habp4 Hk1 Siglec5 Slit2
18/814	0.015394	GO:0016301	kinase activity	Ak4 Aurkc BC016495 Cerk Ckb D8Ertd82e Erbb3 Fgfr2 Galk1 Hk1 Jakmip1 Kit Mapk13 Nrk Nuak1 Prkch Rps6kl1 Stk31
8/280	0.021322	GO:0048037	cofactor binding	Aifm3 Ccbl1 Chdh Ehhadh Ero1l Gch1 Hpgd Pygl
32/1721	0.021883	GO:0016740	transferase activity	A130022J15Rik Agpat9 Ak4 Aurkc BC016495 Ccbl1 Cerk Chpt1 Ckb D8Ertd82e Erbb3 Fgfr2 Galk1 Gstk1 Gstm1 Gstm3 Has3 Hk1 Jakmip1 Kit Mapk13 Nmt2 Nrk Nuak1 Parp12 Prkch Pygl Rps6kl1 St6galnac2 Stk31 Tst Zc3hav1
12/501	0.023829	GO:0008092	cytoskeletal protein binding	Actn2 Afap1 Aif1l Arhgap42 Arpc1b Epb4.1l5 Homer2 Jakmip1 Lcp1 Lmo7 S100a6 Spnb3
15/687	0.02817	GO:0016773	phosphotransferase activity, alcohol group as acceptor	Aurkc BC016495 Cerk D8Ertd82e Erbb3 Fgfr2 Galk1 Hk1 Kit Mapk13 Nrk Nuak1 Prkch Rps6kl1 Stk31
6/197	0.032144	GO:0050662	coenzyme binding	Aifm3 Chdh Ehhadh Ero1l Gch1 Hpgd
5/151	0.03421	GO:0001871	pattern binding	Crispld2 Enpp2 Fgfr2 Habp4 Slit2

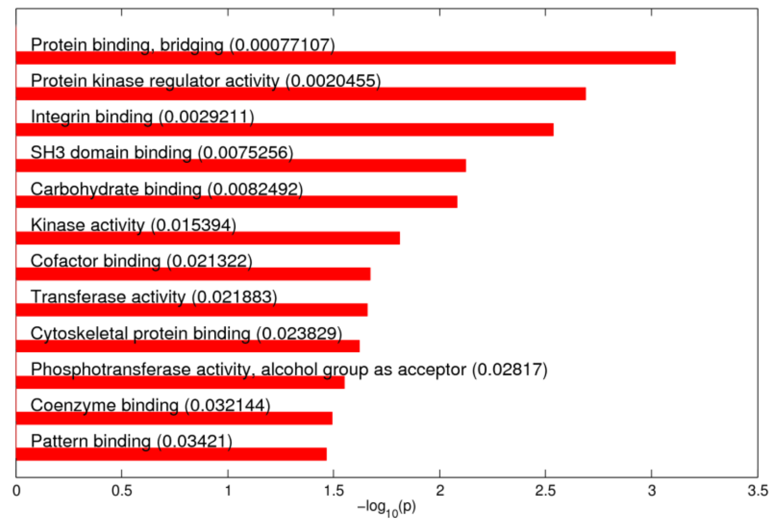

Figure : Plot bar of the -log10(p) of the significant enriched terms of Molecular-function-PGC KO-<-PGC CTRL-Log2(2.8284). The longer the bar, the higher is the statistical significance of the enrichment (in parenthesis are written the p-values)

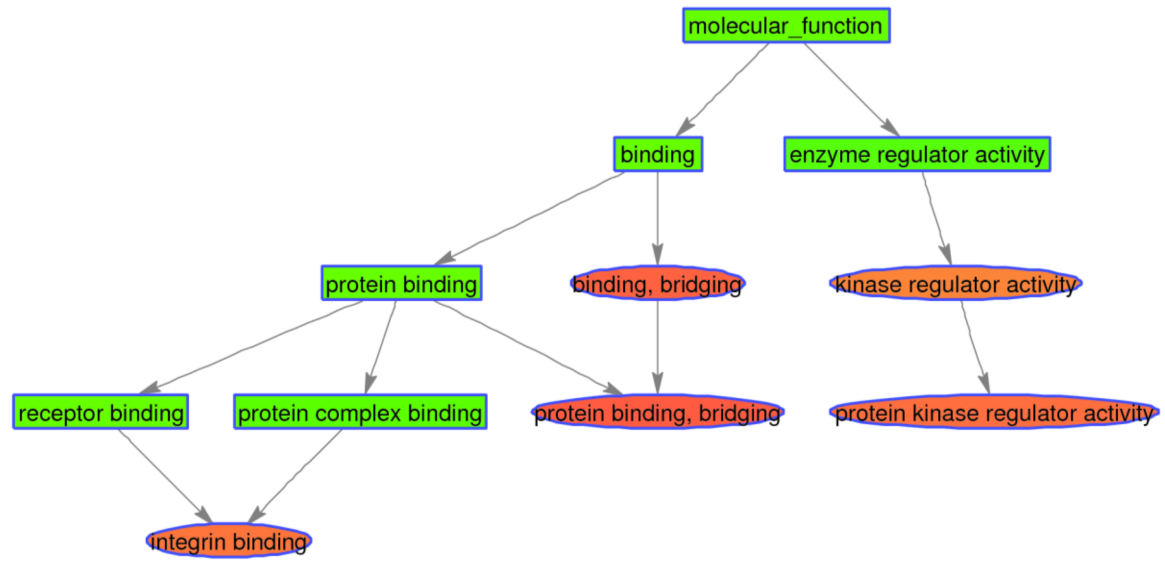

Figure : Direct acyclic graph (DAG) associated to the 5 top higher significant enriched gene ontology terms. The higher the significance of the term the more red is the box framing the term. The significant enriched terms are encircled by ellipses.

Biological-process-PGC KO-<-PGC CTRL-Log2(2.8284)

Table : Significant GO enrichment terms for Biological-process-PGC KO-<-PGC CTRL-Log2(2.8284)

Count Ratio	*p*-val	GO id	GO term	Genes
4/47	0.0012159	GO:0001657	ureteric bud development	Fgfr2 Lhx1 Npnt Slit2
4/49	0.0014283	GO:0034330	cell junction organization	Actn2 Dsp Itgb3 Sorbs1
19/884	0.0069051	GO:0016310	phosphorylation	Aurkc BC016495 Ckb D8Ertd82e Efna1 Erbb3 Fgfr2 Galk1 Hk1 Itga1 Jakmip1 Kit Mapk13 Nrk Nuak1 Prkch Ptpre Rps6kl1 Stk31
6/160	0.0073508	GO:0016042	lipid catabolic process	Ehhadh Enpp2 Homer2 Pla2g4e Plbd1 Plcd1
17/772	0.0079696	GO:0006629	lipid metabolic process	Agpat9 Atp8b1 Cerk Chpt1 Cyp1b1 Ehhadh Enpp2 Gdpd1 Homer2 Hpgd Kit Osbpl3 Pla2g4e Plbd1 Plcd1 Ptges2 Ptgs1
15/657	0.0086626	GO:0006468	protein phosphorylation	Aurkc D8Ertd82e Efna1 Erbb3 Fgfr2 Hk1 Itga1 Kit Mapk13 Nrk Nuak1 Prkch Ptpre Rps6kl1 Stk31
22/1127	0.011887	GO:0006793	phosphorus metabolic process	Acpp Aurkc BC016495 Ckb D8Ertd82e Dnajc6 Efna1 Erbb3 Fgfr2 Galk1 Hk1 Itga1 Jakmip1 Kit Mapk13 Nrk Nuak1 Prkch Ptpre Pygl Rps6kl1 Stk31
7/243	0.017123	GO:0030334	regulation of cell migration	Cxcr4 Efna1 Epb4.1l5 Itgb3 Kit Pecam1 Slit2
7/246	0.018251	GO:0023034	intracellular signal transduction	Dmrt1 Gab2 Plcd1 Prkch Rasgrp2 Rassf5 Sh3bp5
6/196	0.019211	GO:0002009	morphogenesis of an epithelium	Cxcr4 Epb4.1l5 Fgfr2 Gcm1 Npnt Slit2
5/149	0.020617	GO:0001763	morphogenesis of a branching structure	Cxcr4 Fgfr2 Gcm1 Npnt Slit2
6/200	0.021138	GO:0001701	in utero embryonic development	Epb4.1l5 Fgfr2 Gjb3 Kif3a Slc30a1 Slit2
5/151	0.021852	GO:0048858	cell projection morphogenesis	Erbb3 Fgfr2 Itga1 Kif3a Slit2
5/151	0.021923	GO:0090066	regulation of anatomical structure size	Arpc1b Gch1 Itga1 Kank1 Slit2

18/955	0.028686	GO:0032879	regulation of localization	Cxcr4 Efna1 Epb4.1l5 Erbb3 Gab2 Gata2 Hk1 Itgb3 Kctd15 Kif3a Kit Lcp1 Pecam1 Plcd1 Ptgs1 Slc30a1 Slit2 Sorbs1
4/114	0.030141	GO:0010769	regulation of cell morphogenesis involved in differentiation	Efna1 Epb4.1l5 Mbp Slit2
5/164	0.030752	GO:0040017	positive regulation of locomotion	Epb4.1l5 Itgb3 Kit Pecam1 Slit2
5/168	0.033884	GO:0030155	regulation of cell adhesion	Cyth3 Epb4.1l5 Erbb3 Fermt3 Npnt
6/225	0.03645	GO:0048610	cellular process involved in reproduction	Cxcr4 Fgfr2 Kit Rhox5 Sept4 Spa17
12/590	0.038336	GO:0007155	cell adhesion	Cldn5 Dsp Epb4.1l5 Fermt3 Itga1 Itgb3 Lmo7 Ninj1 Npnt Pecam1 Siglec5 Slit2
5/176	0.040794	GO:0044087	regulation of cellular component biogenesis	Arpc1b Epb4.1l5 Kank1 Kit Slit2
4/126	0.042585	GO:0061138	morphogenesis of a branching epithelium	Cxcr4 Fgfr2 Gcm1 Npnt
4/127	0.043802	GO:0032147	activation of protein kinase activity	Efna1 Itga1 Itgb3 Nrk
7/292	0.04418	GO:0003006	developmental process involved in reproduction	Bik Cxcr4 Dmrt1 Fgfr2 Kit Rhox5 Sept4
7/296	0.047299	GO:0007167	enzyme linked receptor protein signaling pathway	Efna1 Erbb3 Fgfr2 Hpgd Kit Ptpre Sorbs1
23/1363	0.048933	GO:0007166	cell surface receptor signaling pathway	Cerk Cxcr4 Dner Efna1 Erbb3 Fgfr2 Gab2 Gng12 Gpr126 Hey1 Homer2 Hpgd Itga1 Itgb3 Kif3a Kit Meg3 Plcd1 Pth1r Ptpre Rgs16 Slit2 Sorbs1

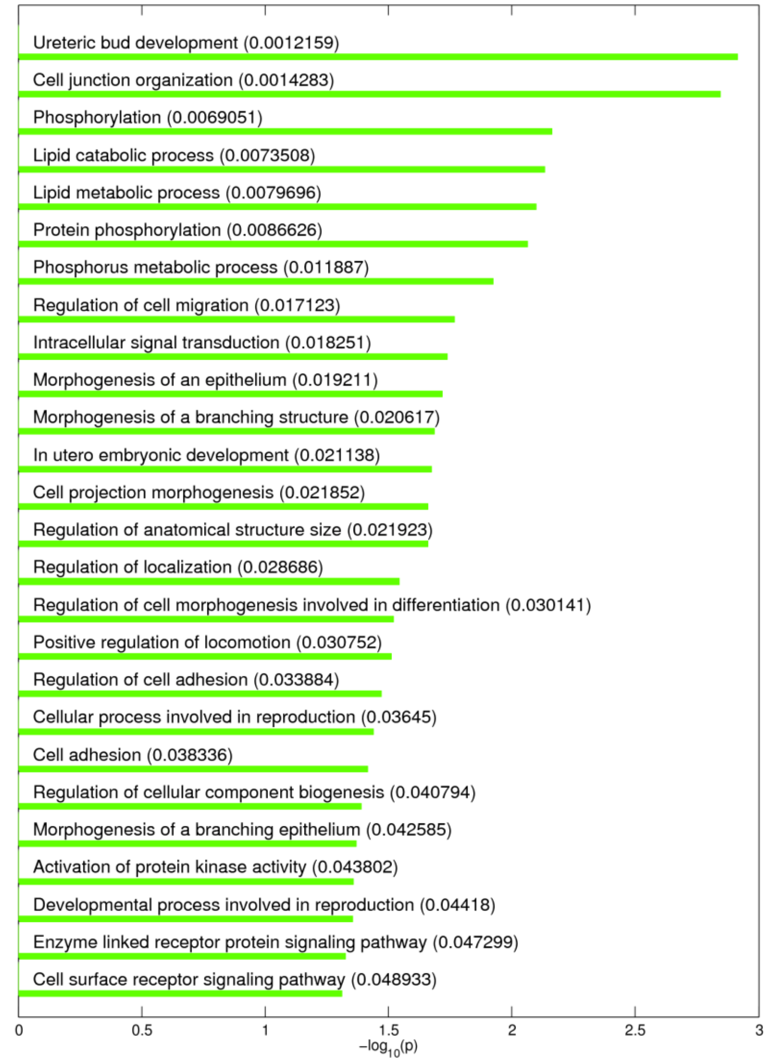

Figure : Plot bar of the -log10(p) of the significant enriched terms of Biological-process-PGC KO-<-PGC CTRL-Log2(2.8284). The longer the bar, the higher is the statistical significance of the enrichment (in parenthesis are written the p-values)

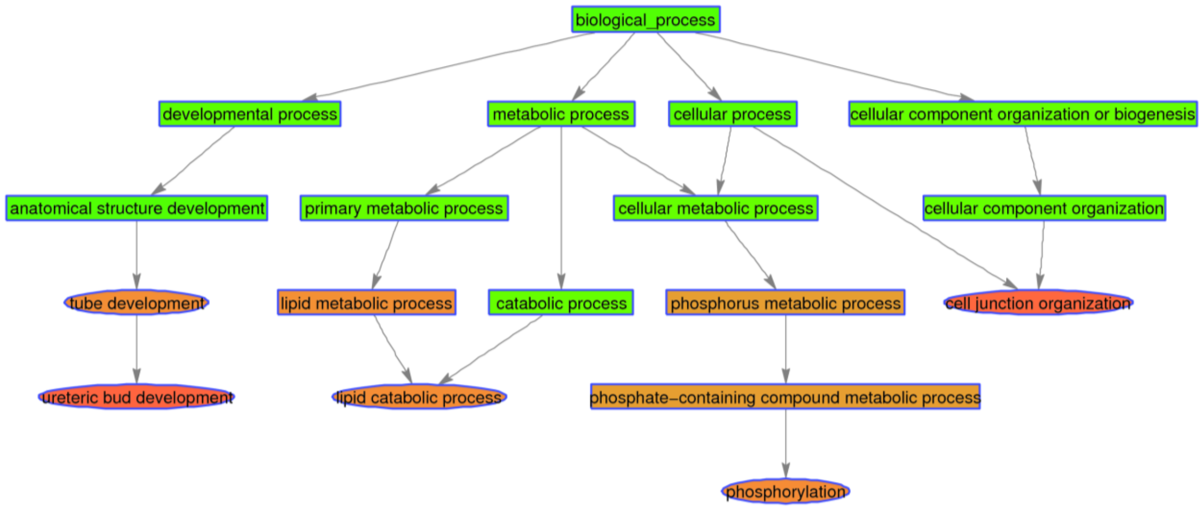

Figure : Direct acyclic graph (DAG) associated to the 5 top higher significant enriched gene ontology terms. The higher the significance of the term the more red is the box framing the term. The significant enriched terms are encircled by ellipses.

Cellular-component-PGC KO-<-PGC CTRL-Log2(2.8284)

Table : Significant GO enrichment terms for Cellular-component-PGC KO-<-PGC CTRL-Log2(2.8284)

Count Ratio	*p*-val	GO id	GO term	Genes
4/22	7.6391e□05	GO:0032587	ruffle membrane	Aif1l Epb4.1l5 Lcp1 Rasgrp2
4/28	0.00020714	GO:0005884	actin filament	Afap1 Aif1l Gng12 Lcp1
17/547	0.0004424	GO:0030054	cell junction	Actn2 Cldn5 Cpeb1 Dsp Epb4.1l5 Fermt3 Gjb3 Homer2 Lcp1 Lmo7 Pecam1 Rasgrp2 Rimbp2 Rims1 Sorbs1 Tes Trim25
7/147	0.0016306	GO:0005912	adherens junction	Actn2 Dsp Epb4.1l5 Lmo7 Sorbs1 Tes Trim25
6/118	0.0023882	GO:0031253	cell projection membrane	Aif1l Atp8b1 Epb4.1l5 Lcp1 Pth1r Rasgrp2
5/84	0.0025726	GO:0005925	focal adhesion	Actn2 Epb4.1l5 Sorbs1 Tes Trim25
95/5867	0.0064927	GO:0016020	membrane	4632428N05Rik 6030446N20Rik Acpp Agpat9 Aif1l Aifm3 Ap3b2 Ap3m2 Atp8b1 Bcap29 Btbd11 Car12 Cd164l2 Cd276 Cerk Chdh Chpt1 Cldn5 Cpeb1 Cxcr4 Cyp1b1 Cyth3 Diras2 Dner E130309F12Rik Efna1 Enpp4 Epb4.1l5 Erbb3 Ero1l Fam19a5 Fgfr2 Gab2 Gca Gdpd1 Gjb3 Gng12 Gpr126 Gpr137b Gstk1 H2-Bl Has3 Hk1 Homer2 Ier3 Immp2l Itga1 Itgb3 Jakmip1 Kctd15 Kit Lcp1 Ly6a Marveld3 Mbp Meg3 Mep1b Mrs2 Ninj1 Npnt Pecam1 Pla2g4e Plcd1 Pnkd Prkch Ptges2 Ptgs1 Pth1r Ptpre Rab6b Rasgrp2 Rffl Rgs16 Rimbp2 Rims1 Rnf182 S100a6...
25/1203	0.0092743	GO:0044459	plasma membrane part	Aif1l Atp8b1 Cd276 Dsp Efna1 Enpp2 Epb4.1l5 Erbb3 Gjb3 Gng12 H2-Bl Hk1 Itga1 Itgb3 Kctd15 Kit Lcp1 Lmo7 Ly6a Pth1r Rasgrp2 S100a6 Slc30a1 Slco4c1 Sorbs1
6/192	0.026694	GO:0045121	membrane raft	Hk1 Itga1 Pecam1 Plcd1 Rgs16 Sorbs1
19/947	0.029137	GO:0044430	cytoskeletal part	1190002H23Rik Actn2 Afap1 Aif1l Arpc1b Aurkc Cpeb1 Fermt3 Gng12 Homer2 Jakmip1 Kif3a Lcp1 Pcgf5 Rassf5 Sept4 Sfi1 Sorbs1 Spnb3
38/2240	0.038426	GO:0005886	plasma membrane	Aif1l Cldn5 Cpeb1 Cxcr4 Cyth3 Diras2 Dner Efna1 Epb4.1l5 Gab2 Gjb3 Gng12 Gpr126 H2-Bl Homer2 Lcp1 Ly6a Mbp Meg3 Pecam1 Prkch Ptgs1 Pth1r Ptpre Rasgrp2 Rgs16 Rimbp2 Rims1 S100a6 Slc30a1 Slc38a4 Slc40a1 Slc5a5 Slco4c1 Slit2 Sorbs1 Tst Ttyh2

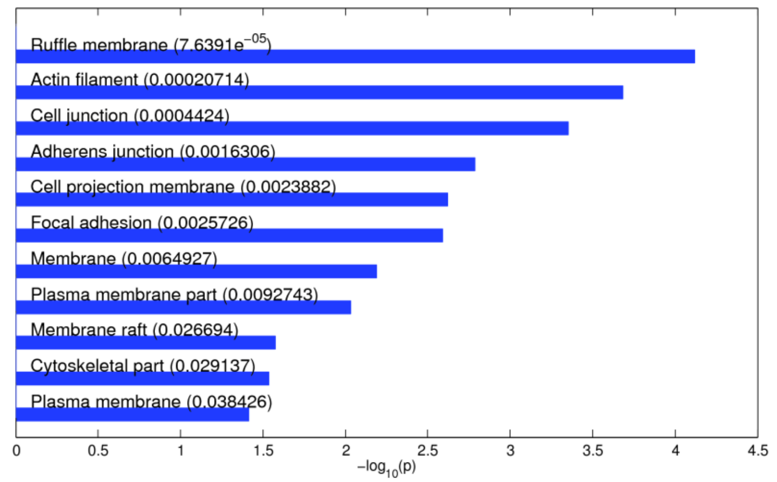

Figure : Plot bar of the -log10(p) of the significant enriched terms of Cellular-component-PGC KO-<-PGC CTRL-Log2(2.8284). The longer the bar, the higher is the statistical significance of the enrichment (in parenthesis are written the p-values)

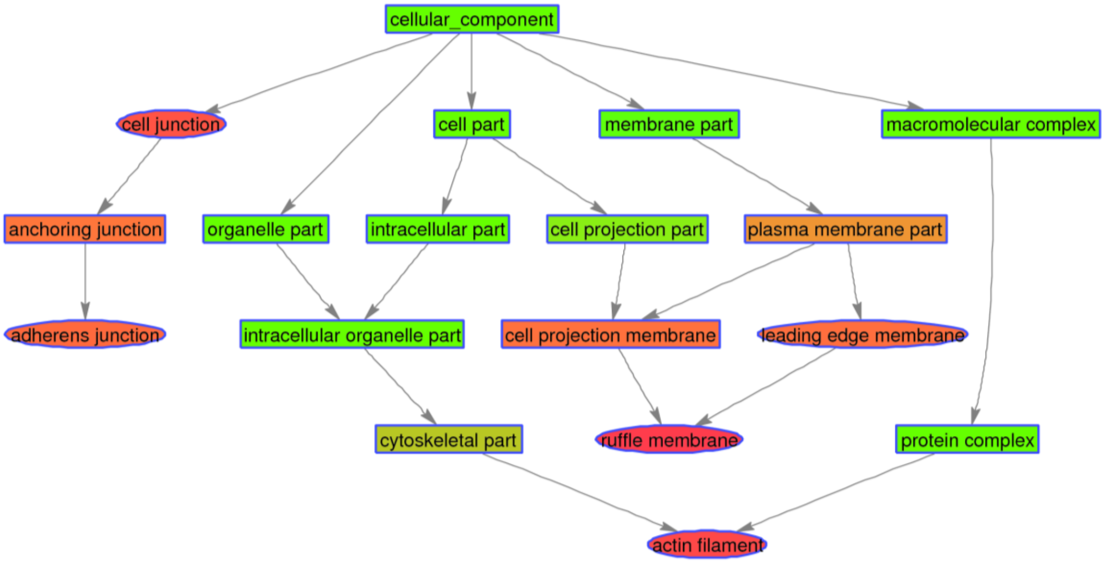

Figure : Direct acyclic graph (DAG) associated to the 5 top higher significant enriched gene ontology terms. The higher the significance of the term the more red is the box framing the term. The significant enriched terms are encircled by ellipses.

2.5 Disregulated (down- or up-regulated) in PGC KO in relation to PGC CTRL

Number of probes fulfilling the condition: 455. (These probes have already been listed in the previous heatmaps).

2.6 GO enrichment analysis of PGC KO-<>-PGC CTRL-Log2(2.8284)

Number of probes in the signal set 455

Number of unique probes in the signal set 455

Number of probes in the background set 45101

Number of unique probes in the background set 21390

Table : GO terms of PGC KO-<>-PGC CTRL-Log2(2.8284)-molecular-function

GO	Ter
GO:0000166	nucleotide binding (3.1%)
GO:0003824	catalytic activity (3.4%)
GO:0017076	purine nucleotide binding (8.5%)
GO:0005515	protein binding (9.1%)
GO:0005488	binding (10%)
GO:0043167	ion binding (12%)
Others	Others (54%)

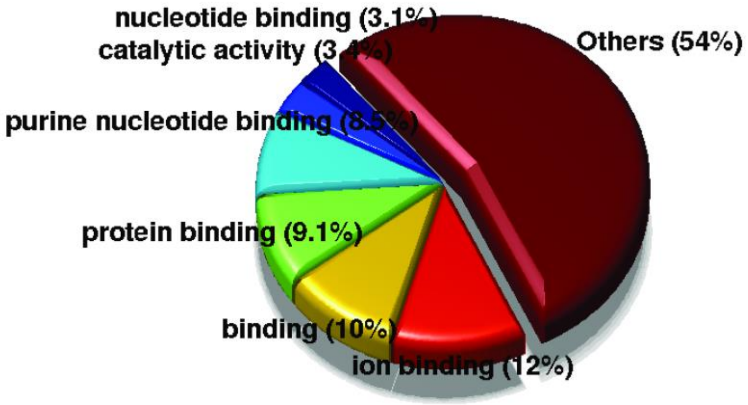

Figure 28: Pie graph of PGC KO-<>-PGC CTRL-Log2(2.8284)-molecular-function

Table : GO terms of PGC KO-<>-PGC CTRL-Log2(2.8284)-biological-process

GO	Ter
GO:0050793	regulation of developmental process (3.1%)
GO:0006810	transport (3.3%)
GO:0050789	regulation of biological process (3.3%)
GO:0009889	regulation of biosynthetic process (3.3%)
GO:0060255	regulation of macromolecule metabolic process (3.8%)
GO:0016043	cellular component organization (3.8%)
GO:0050896	response to stimulus (4.8%)
GO:0032502	developmental process (8.9%)
GO:0050794	regulation of cellular process (9.6%)
Others	Others (56%)

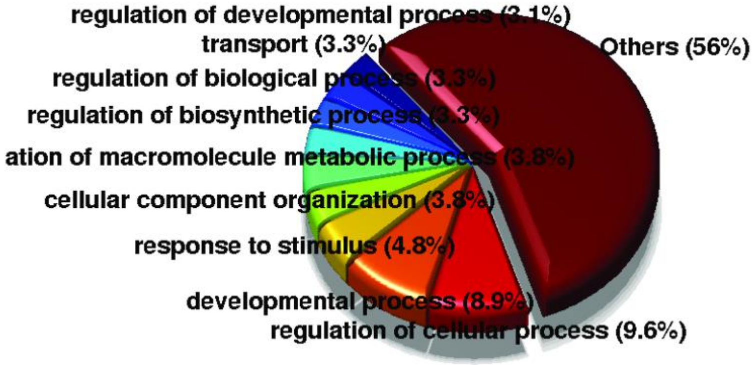

Figure 29: Pie graph of PGC KO-<>-PGC CTRL-Log2(2.8284)-biological-process

Table : GO terms of PGC KO-<>-PGC CTRL-Log2(2.8284)-cellular-component

GO	Ter
GO:0044459	plasma membrane part (3.2%)
GO:0044422	organelle part (3.2%)
GO:0043234	protein complex (3.7%)
GO:0044425	membrane part (4.1%)
GO:0043227	membrane-bounded organelle (4.9%)
GO:0044444	cytoplasmic part (5%)
GO:0043229	intracellular organelle (5.7%)
GO:0043226	organelle (5.7%)
GO:0031224	intrinsic to membrane (6.3%)
GO:0016020	membrane (7.4%)
GO:0043231	intracellular membrane-bounded organelle (11%)
GO:0044424	intracellular part (11%)
Others	Others (29%)

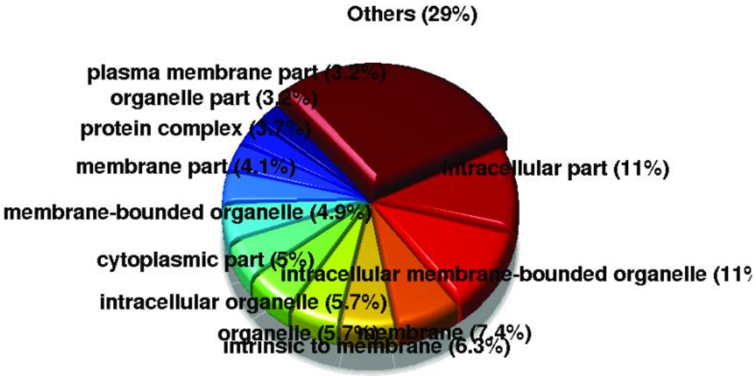

Figure 30: Pie graph of PGC KO-<>-PGC CTRL-Log2(2.8284)-cellular-component

Molecular-function-PGC KO-<>-PGC CTRL-Log2(2.8284)

Table : Significant GO enrichment terms for Molecular-function-PGC KO-<>-PGC CTRL-Log2(2.8284)

Count Ratio	*p*-val	GO id	GO term	Genes
4/16	0.00048901	GO:0004089	carbonate dehydratase activity	Car12 Car2 Car3 Car4
10/106	0.00058826	GO:0017124	SH3 domain binding	Afap1 Arhgap42 Dnajc6 Dock4 Dpysl3 Enah Lrp2 Mical1 Rims1 Sh3bp5
26/458	0.00062166	GO:0019904	protein domain specific binding	Actn2 Afap1 Akap2 Arhgap42 Bik Ccdc88c Cited2 Dmd Dnajc6 Dock4 Dpysl3 Ebf1 Enah Epb4.1l5 Gab2 Lnx1 Lrp2 Mbd2 Mical1 Ncam1 Nefl Nr0b1 Pten Rims1 Robo1 Sh3bp5
7/64	0.0014051	GO:0030674	protein binding, bridging	Actn2 Actn3 Arhgap42 Dsp Gab2 Grb10 Sorbs1
26/501	0.0022429	GO:0008092	cytoskeletal protein binding	Actn2 Actn3 Afap1 Aif1l Arhgap42 Arpc1b Ccdc88c Coro2b Dmd Enah Epb4.1l5 Epb4.9 Fez1 Gli1 Homer2 Jakmip1 Kif1b Lcp1 Lmo7 Mtap1b Myo1f Parvb Psrc1 S100a6 Spnb3 Trim2
6/54	0.0026586	GO:0005178	integrin binding	Ctgf Dmd Fermt3 Itga1 Itgb3 Npnt
36/774	0.0027753	GO:0001071	nucleic acid binding transcription factor activity	Aff1 Cited2 Cphx Csrnp3 Dmrt1 Ebf1 Epas1 Foxp1 Gata2 Gbx2 Gcm1 Gli1 Gli2 Glis3 Hey1 Hhex Hoxa5 Id1 Klf12 Klf4 Lhx1 Mkx Neurod1 Nr0b1 Nr3c1 Pou4f2 Rhox4a Rhox5 Rhox6 Rhox9 Tcf7l2 Tcfap2c Tgif2 Trps1 Zfhx3 Zfp423
4/25	0.0030518	GO:0004364	glutathione transferase activity	Gstk1 Gstm1 Gstm3 Gstt2

8/98	0.0048095	GO:0019207	kinase regulator activity	1190002H23Rik Camk2n1 Ccnd1 Efcab10 Erbb3 Klf4 Sh3bp5 Spa17
20/375	0.0048607	GO:0019001	guanyl nucleotide binding	Ak4 Diras2 Dock4 Eras Gbp2 Gbp3 Gch1 Mras Prps1 Prps2 Rab31 Rab6b Rnd3 Rtkn Sept4 Tgtp1 Tubb2a Tubb2a-ps2 Tubb2b Urgcp
7/79	0.0050111	GO:0019887	protein kinase regulator activity	1190002H23Rik Camk2n1 Ccnd1 Efcab10 Erbb3 Sh3bp5 Spa17
5/44	0.0052111	GO:0016836	hydro-lyase activity	Car12 Car2 Car3 Car4 Ehhadh
13/211	0.0061258	GO:0003682	chromatin binding	Chd7 Foxp1 Gli1 Gli2 Hhex Hmgn5 Mbd2 Nr3c1 Phf21a Pou4f2 Smarca1 Sox15 Trps1
4/32	0.007987	GO:0004601	peroxidase activity	Gpx7 Gstk1 Homer2 Ptgs1
19/367	0.0083003	GO:0030695	GTPase regulator activity	Arhgap42 Arhgap8 Bcar3 Cyth3 Dock4 Grtp1 Iqgap2 Mcf2 Nrk Plce1 Rab3il1 Ralgds Rasgrp2 Rgs16 Rgs19 Rtkn Slit2 Srgap2 Tiam1
5/49	0.0085522	GO:0016209	antioxidant activity	Gpx7 Gstk1 Homer2 Ptgs1 Srxn1
26/564	0.011048	GO:0005509	calcium ion binding	Actn2 Actn3 Aif1l Anxa4 Anxa8 Cdhr1 Dmd Dner Dtna Efcab10 Gca Gch1 Lcp1 Lrp2 Nid1 Notch4 Npnt Pla2g1b Plcd1 Plcd3 Plce1 Rasgrp2 S100a10 S100a6 Slit2 Sulf1
33/766	0.012841	GO:0030234	enzyme regulator activity	1190002H23Rik Aifm3 Arhgap42 Arhgap8 Bcar3 Camk2n1 Ccnd1 Cyth3 Dnmt3l Dock4 Efcab10 Erbb3 Grtp1 Iqgap2 Klf4 Mcf2 Nrk Plce1 Rab3il1 Ralgds Rasgrp2 Rgs16 Rgs19 Rtkn Serpinb6c Serpine2 Sfrp2 Sh3bp5 Slit2 Spa17 Spink3 Srgap2 Tiam1
19/400	0.020036	GO:0030528	transcription regulator activity	Cited2 Ebf1 Epas1 Gata2 Gbx2 Hey1 Hhex Hoxa5 Id1 Klf9 Lhx1 Mkx Neurod1 Nr3c1 Pou4f2 Smarca1 Sox15 Tcfl5 Zfhx3
19/401	0.020636	GO:0042803	protein homodimerization activity	Actn2 Actn3 Ccbl1 Foxp1 Gca Gstm1 Hhex Hk1 Inhbb Irak3 Nr0b1 Nr3c1 Prps1 Prps2 Ptpre Pygl S100a6 Slit2 Zfp423
25/569	0.02219	GO:0043565	sequence-specific DNA binding	Cphx Epas1 Foxp1 Gata2 Gbx2 Gli2 Hhex Hoxa5 Klf4 Lhx1 Mbd2 Mkx Neurod1 Nr0b1 Nr3c1 Pou4f2 Rhox4a Rhox5 Rhox6 Rhox9 Tcf7l2 Tgif2 Trps1 Zfhx3 Zfp423
5/61	0.022337	GO:0004714	transmembrane receptor protein tyrosine kinase activity	Epha2 Erbb3 Fgfr2 Kit Tek
6/82	0.023163	GO:0016298	lipase activity	Abhd5 Pla2g1b Pla2g4e Plcd1 Plcd3 Plce1
9/150	0.023931	GO:0005085	guanyl-nucleotide exchange factor activity	Bcar3 Cyth3 Dock4 Mcf2 Plce1 Rab3il1 Ralgds Rasgrp2 Tiam1
4/43	0.024769	GO:0016667	oxidoreductase activity, acting on a sulfur group of donors	Ero1l Gstk1 Ptges2 Srxn1
9/151	0.025201	GO:0001871	pattern binding	Crispld2 Ctgf Dpysl3 Enpp2 Fgfr2 Habp4 Ncam1 Serpine2 Slit2
8/131	0.029348	GO:0005539	glycosaminoglycan binding	Crispld2 Ctgf Dpysl3 Fgfr2 Habp4 Ncam1 Serpine2 Slit2
5/65	0.030036	GO:0015171	amino acid transmembrane transporter activity	Slc38a4 Slc6a8 Slc7a3 Slc7a9 Slco4a1
15/310	0.032248	GO:0003779	actin binding	Actn2 Actn3 Afap1 Aif1l Arpc1b Coro2b Dmd Enah Epb4.9 Homer2 Lcp1 Mtap1b Myo1f Parvb Spnb3
5/68	0.036242	GO:0004620	phospholipase activity	Pla2g1b Pla2g4e Plcd1 Plcd3 Plce1
12/237	0.038024	GO:0030246	carbohydrate binding	Crispld2 Ctgf Dpysl3 Enpp2 Fgfr2 Galk1 Habp4 Hk1 Ncam1 Serpine2 Siglec5 Slit2
26/634	0.045395	GO:0046983	protein dimerization activity	Actn2 Actn3 Bik Ccbl1 Epas1 Erbb3 Foxp1 Gca Gstm1 Hhex Hk1 Inhbb Irak3 Itga1 Neurod1 Nr0b1 Nr3c1 Prps1 Prps2 Ptpre Pygl Robo1 S100a6 Slit2 Tcfap2c Zfp423

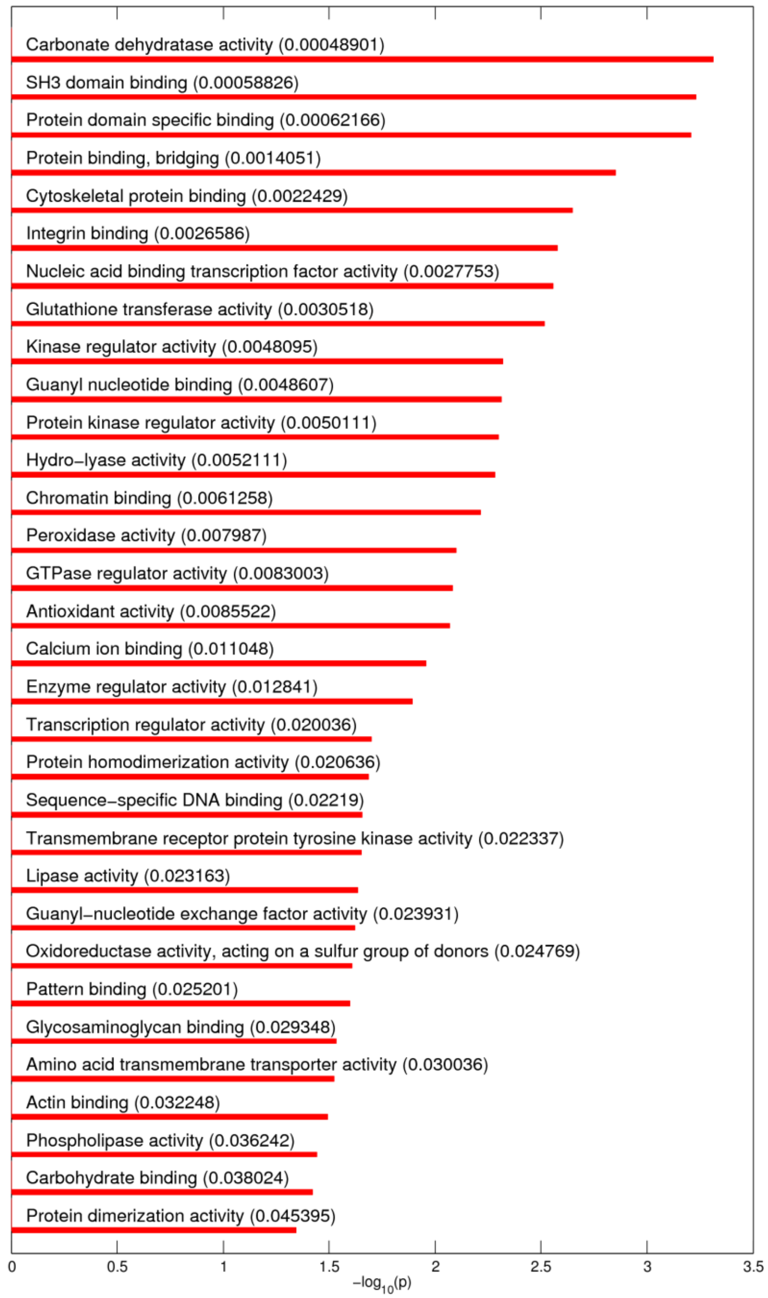

Figure 31: Plot bar of the -log10(p) of the significant enriched terms of Molecular-function-PGC KO-<>-PGC CTRL-Log2(2.8284). The longer the bar, the higher is the statistical significance of the enrichment (in parenthesis are written the p-values)

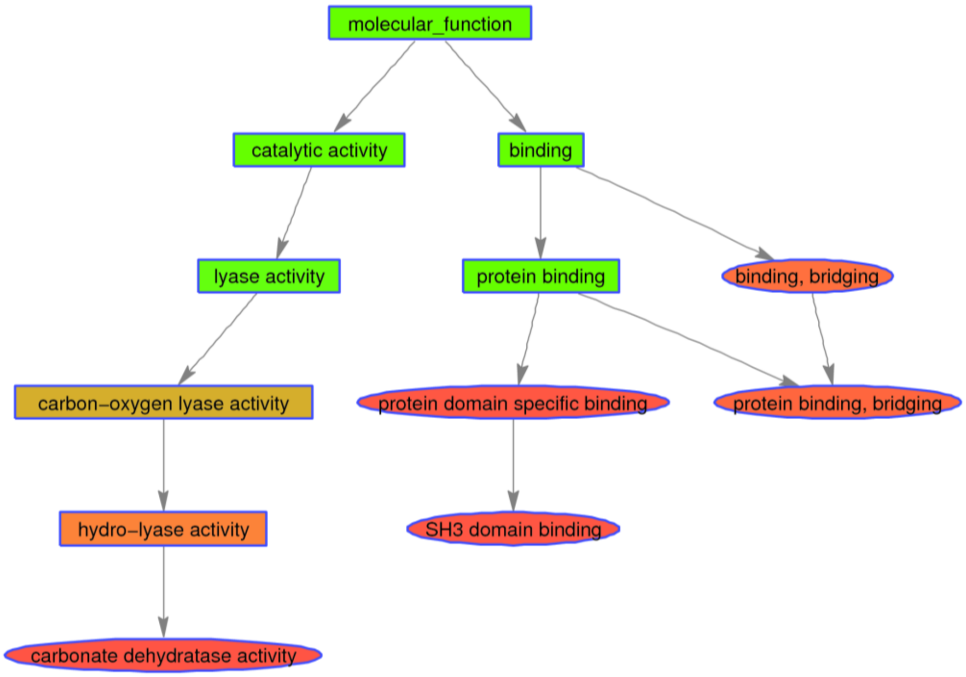

Figure 32: Direct acyclic graph (DAG) associated to the 5 top higher significant enriched gene ontology terms. The higher the significance of the term the more red is the box framing the term. The significant enriched terms are encircled by ellipses.

Biological-process-PGC KO-<>-PGC CTRL-Log2(2.8284)

Table : Significant GO enrichment terms for Biological-process-PGC KO-<>-PGC CTRL-Log2(2.8284)

Count Ratio	*p*-val	GO id	GO term	Genes
18/196	4.3137e□05	GO:0002009	morphogenesis of an epithelium	Car2 Cited2 Cxcr4 Epb4.1l5 Fgfr2 Gbx2 Gcm1 Gli2 Hhex Hoxa5 Lama1 Notch4 Npnt Nr3c1 Sfrp1 Sfrp2 Slit2 Vegfc
47/817	8.0174e□05	GO:0048513	organ development	Anxa4 Bik Car2 Car4 Ccnd1 Cdk6 Chd7 Cited2 Ckb Crip2 Ctgf Cxcr4 Dmd Epas1 Erbb3 Fgf15 Fgfr2 Foxp1 Gata2 Gjb3 Gli1 Gli2 Hhex Hoxa5 Id1 Inhbb Kif3a Kit Klf4 Lhx1 Lmna Mkx Neurod1 Notch4 Nr0b1 Nr3c1 Plcd1 Plcd3 Pten Sept4 Sfrp1 Sfrp2 Slit2 Smarca1 Tek Trps1 Zfhx3
62/1196	0.00014464	GO:0030154	cell differentiation	Abhd5 Bcl11a Ccnd1 Cdk6 Chd7 Cited2 Ctgf Dmrt1 Dner Dsp Efna1 Enah Epas1 Epha2 Erbb3 Ero1l Fgf4 Fgfr2 Foxp1 Gab2 Gabbr1 Gata2 Gcm1 Gli1 Gli2 Hey1 Hhex Hoxa5 Inhbb Kit Klf4 Lama1 Lhx1 Ndrg2 Neurod1 Notch4 Npnt Nr0b1 Paqr8 Pou4f2 Ptgs1 Pth1r Robo1 Sema4a Serpine2 Sfrp1 Sfrp2 Slco4c1 Slit2 Smarca1 Sox15 Spp1 Tcf7l2 Tcfap2c Tubb2a Tubb2a-ps2 Tubb2b Vegfc Zfp36l1 Zfp423 Zic2 Zic5
77/1584	0.00020161	GO:0048869	cellular developmental process	Abcb1b Abhd5 Bcl11a Calca Ccnd1 Cdk6 Chd7 Cited2 Ctgf Cxcr4 Dmd Dmrt1 Dner Dsp Efna1 Enah Epas1 Epb4.1l5 Epha2 Erbb3 Ero1l Fgf4 Fgfr2 Foxp1 Gab2 Gabbr1 Gata2 Gcm1 Gli1 Gli2 Hey1 Hhex Hoxa5 Id1 Inhbb Itga1 Kif3a Kit Klf4 Lama1 Lhx1 Lmna Msi2 Mtap1b Ncam1 Ndrg2 Nefl Neurod1 Notch4 Npnt Nr0b1 Paqr8 Pou4f2 Ptgs1 Pth1r Rhox5 Robo1 Sema4a Sept4 Serpine2 Sfrp1 Sfrp2 Slco4c1 Slit2 Smarca1 Sox15 Spp1 Tcf7l2 Tcfap2c Tubb2a Tubb2a-ps2 Tubb2b Vegfc Zfp36l1 Zfp423 Zic2 Zic5
14/149	0.00021012	GO:0001763	morphogenesis of a branching structure	Cited2 Cxcr4 Fgfr2 Gbx2 Gcm1 Gli2 Hhex Hoxa5 Lama1 Notch4 Npnt Sfrp1 Sfrp2 Slit2
15/168	0.0002309	GO:0030155	regulation of cell adhesion	Calca Cdk6 Cited2 Cyth3 Epb4.1l5 Erbb3 Fermt3 Lama1

				Myo1f Nid1 Npnt Pten Serpine2 Spp1 Tek
6/33	0.00031546	GO:0060562	epithelial tube morphogenesis	Gli2 Hhex Hoxa5 Lama1 Nr3c1 Slit2
50/955	0.00045668	GO:0009966	regulation of signal transduction	Afap1 Agpat9 Ccnd1 Ceacam1 Cited2 Cyth3 Ddit4l Efna1 Erbb3 Fgf15 Fgf4 Fgfbp1 Fgfr2 Gli2 Grb10 Hey1 Hhex Id1 Iqgap2 Irak3 Itga1 Itgb3 Kit Klf4 Mbd2 Mcf2 Ncam1 Npnt Nr0b1 Nr3c1 Plce1 Plk2 Pten Ptpre Ralgds Rasgrp2 Rgs16 Rgs19 Robo1 Serpine2 Sfrp1 Sfrp2 Slc7a3 Slit2 Spred1 Tcf7l2 Tek Tiam1 Tob1 Vegfc
5/24	0.0004717	GO:0051385	response to mineralocorticoid stimulus	Ccnd1 Ctgf Nefl Ptgs1 Trh
12/126	0.00048434	GO:0061138	morphogenesis of a branching epithelium	Cited2 Cxcr4 Fgfr2 Gbx2 Gcm1 Hhex Hoxa5 Lama1 Notch4 Npnt Sfrp1 Sfrp2
83/1799	0.00059484	GO:0048856	anatomical structure development	Abcb1b Anxa4 Bik Car2 Car4 Ccnd1 Cdk6 Chd7 Cited2 Ckb Crip2 Ctgf Cxcr4 Dmd Dner Dnmt3l Dpysl3 Dsp E130309F12Rik Enah Epas1 Epb4.1l5 Epha2 Erbb3 Fgf15 Fgfr2 Foxp1 Gata2 Gbx2 Gjb3 Gli1 Gli2 Gng12 Hhex Hoxa5 Id1 Inhbb Kif1b Kif3a Kit Klf4 Lama1 Lhx1 Lmna Lrp2 Mcf2 Mkx Msi2 Mtap1b Ncam1 Ndrg2 Neurod1 Ninj1 Notch4 Npnt Nr0b1 Nr3c1 Plcd1 Plcd3 Plce1 Pou4f2 Prps1 Pten Pth1r Robo1 Sema4a Sept4 Serpine2 Sfrp1 Sfrp2 Slc30a1 Slit2 Smarca1 Sox15 Spp1 Tcf7l2 Tek Tgif2 Trps1 Zfhx3 Zfp423 Zic2 Zic5
37/659	0.00063854	GO:0045595	regulation of cell differentiation	Adamts9 Car2 Ccnd1 Cd276 Cdk6 Dpysl3 Efna1 Epb4.1l5 Fez1 Fgf4 Fgfr2 Gli2 Hoxa5 Itgb3 Kit Klf4 Mbp Mtap1b Nefl Neurod1 Notch4 Nr0b1 Pou4f2 Pten Robo1 Serpine2 Sfrp1 Sfrp2 Slit2 Spp1 Tcf7l2 Tcfl5 Tgif2 Tiam1 Tob1 Vegfc Zfhx3
48/923	0.00067213	GO:0050793	regulation of developmental process	Adamts9 Akap2 Car2 Ccnd1 Cd276 Cdk6 Dpysl3 Efna1 Epb4.1l5 Epha2 Fez1 Fgf4 Fgfr2 Gata2 Gli2 Hhex Hoxa5 Id1 Irak3 Itgb3 Kit Klf4 Lama1 Lmna Mbp Mtap1b Ncam1 Nefl Neurod1 Notch4 Nr0b1 Pou4f2 Pten Robo1 Serpine2 Sfrp1 Sfrp2 Slit2 Sox15 Spp1 Tcf7l2 Tcfl5 Tek Tgif2 Tiam1 Tob1 Vegfc Zfhx3
58/1170	0.00068443	GO:0023051	regulation of signaling	Afap1 Agpat9 Ccnd1 Ceacam1 Chd7 Cited2 Cpeb1 Cyth3 Ddit4l Efna1 Erbb3 Fgf15 Fgf4 Fgfbp1 Fgfr2 Gabbr1 Gli2 Grb10 Hey1 Hhex Id1 Inhbb Iqgap2 Irak3 Itga1 Itgb3 Kit Klf4 Mbd2 Mcf2 Mtap1b Ncam1 Npnt Nr0b1 Nr3c1 Plce1 Plk2 Pten Ptpre Ralgds Rasgrp2 Rgs16 Rgs19 Rims1 Robo1 Serpine2 Sfrp1 Sfrp2 Slc30a1 Slc7a3 Slit2 Spred1 Tcf7l2 Tek Tiam1 Tob1 Trh Vegfc
7/52	0.00076952	GO:0071363	cellular response to growth factor stimulus	Ctgf Epb4.1l5 Fez1 Fgf15 Fgf17 Fgf4 Fgfr2
9/82	0.00077427	GO:0045785	positive regulation of cell adhesion	Calca Cdk6 Cited2 Cyth3 Epb4.1l5 Nid1 Npnt Spp1 Tek
7/54	0.00097316	GO:0007417	central nervous system development	Chd7 Cited2 Dner Mtap1b Pten Robo1 Zic2
4/17	0.00098253	GO:0051412	response to corticosterone stimulus	Ccnd1 Nefl Ptgs1 Trh
4/17	0.000984	GO:0090287	regulation of cellular response to growth factor stimulus	Grb10 Hhex Slit2 Vegfc
20/296	0.0011046	GO:0007167	enzyme linked receptor protein signaling pathway	Akap2 Ctgf Efna1 Epha2 Erbb3 Fgf15 Fgf17 Fgf4 Fgfr2 Grb10 Hpgd Id1 Inhbb Kit Pten Ptpre Sorbs1 Tek Tiam1 Vegfc
5/29	0.0012035	GO:0006730	one-carbon metabolic process	Car12 Car2 Car3 Car4 Gch1
4/18	0.0012462	GO:0033280	response to vitamin D	Abcb1b Lrp2 Spp1 Trim25
8/72	0.0013233	GO:0050770	regulation of axonogenesis	Efna1 Mbp Mtap1b Nefl Robo1 Slit2 Spp1 Tiam1
11/123	0.0013586	GO:0048754	branching morphogenesis of a tube	Cited2 Cxcr4 Gbx2 Gli2 Hhex Hoxa5 Lama1 Notch4 Npnt Sfrp2 Slit2
5/30	0.0014216	GO:0016331	morphogenesis of embryonic epithelium	Epb4.1l5 Fgfr2 Gli2 Hhex Vegfc

5/30	0.0014238	GO:0021915	neural tube development	Epha2 Gli2 Sfrp1 Sfrp2 Tcf7l2
12/144	0.0016203	GO:0010975	regulation of neuron projection development	Dpysl3 Efna1 Fez1 Mbp Mtap1b Nefl Pten Robo1 Serpine2 Slit2 Spp1 Tiam1
15/202	0.00163	GO:0007169	transmembrane receptor protein tyrosine kinase signaling pathway	Ctgf Efna1 Epha2 Erbb3 Fgf15 Fgf17 Fgf4 Fgfr2 Grb10 Kit Pten Sorbs1 Tek Tiam1 Vegfc
5/31	0.0016686	GO:0046324	regulation of glucose import	Erbb3 Fgf15 Grb10 Pea15a Sorbs1
10/109	0.0017699	GO:0048732	gland development	Cited2 Fgfr2 Gata2 Gli1 Gli2 Hhex Hoxa5 Notch4 Nr0b1 Nr3c1
10/110	0.0018991	GO:0033273	response to vitamin	Abca1 Abcb1b Ccnd1 Klf4 Lefty1 Lrp2 Mtap1b Spp1 Tek Trim25
5/32	0.0019438	GO:0008543	fibroblast growth factor receptor signaling pathway	Ctgf Fgf15 Fgf17 Fgf4 Fgfr2
19/292	0.0022394	GO:0003006	developmental process involved in reproduction	Bik Calca Ccnd1 Chd7 Cited2 Cxcr4 Dmrt1 Fgfr2 Gli2 Inhbb Kit Nr0b1 Pten Rhox5 Sept4 Serpine2 Sfrp1 Sfrp2 Tcf7l2
8/78	0.0022735	GO:0048568	embryonic organ development	Cited2 Epas1 Fgfr2 Gata2 Gli2 Hhex Plcd1 Plcd3
60/1285	0.0023357	GO:0048583	regulation of response to stimulus	Afap1 Agpat9 B2m Calca Ccnd1 Cd276 Ceacam1 Cited2 Cxcr4 Cyth3 Ddit4l Efna1 Erbb3 F2rl1 Fgf15 Fgf4 Fgfbp1 Fgfr2 Gab2 Gli2 Grb10 H2-Bl Hey1 Hhex Id1 Iqgap2 Irak3 Itga1 Itgb3 Kit Klf4 Mbd2 Mcf2 Myo1f Ncam1 Npnt Nr0b1 Nr3c1 Plce1 Plk2 Pten Ptpre Ralgds Rasgrp2 Rgs16 Rgs19 Robo1 Serpine2 Sfrp1 Sfrp2 Slc7a3 Slit2 Spp1 Spred1 Tcf7l2 Tek Tiam1 Tob1 Vegfc Zc3hav1
27/474	0.0025059	GO:0048731	system development	Chd7 Cited2 Cxcr4 Dner Dpysl3 E130309F12Rik Enah Epha2 Erbb3 Gbx2 Gli2 Hoxa5 Id1 Lhx1 Mtap1b Ndrg2 Neurod1 Pou4f2 Prps1 Pten Pth1r Robo1 Sema4a Serpine2 Slit2 Zfp423 Zic2

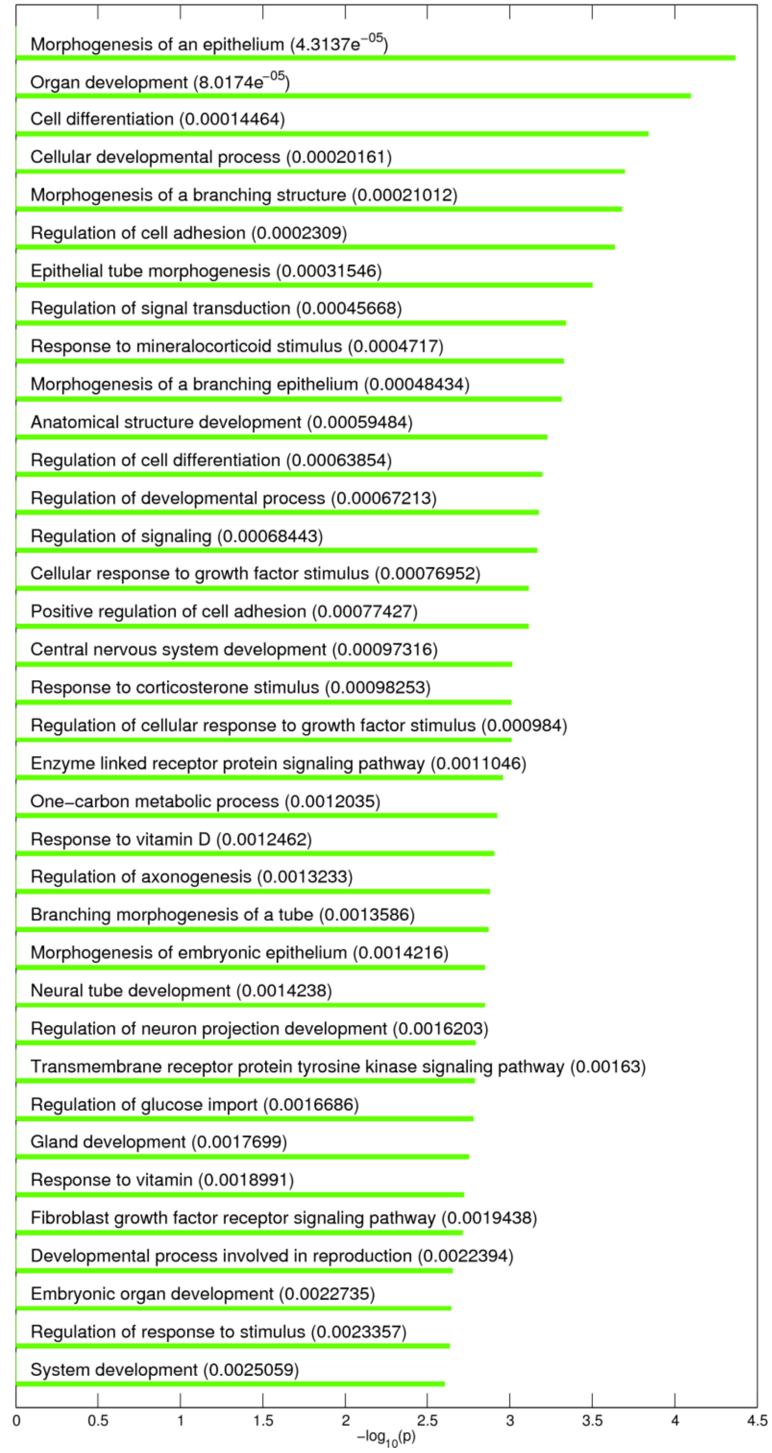

Figure 33: Plot bar of the -log10(p) of the significant enriched terms of Biological-process-PGC KO-<>-PGC CTRL-Log2(2.8284). The longer the bar, the higher is the statistical significance of the enrichment (in parenthesis are written the p-values)

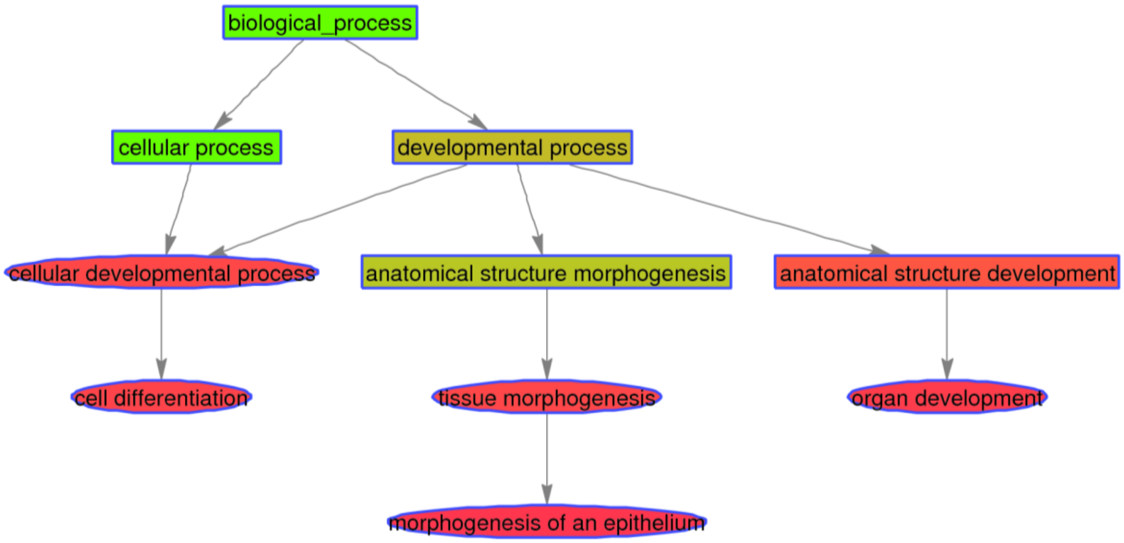

Figure 34: Direct acyclic graph (DAG) associated to the 5 top higher significant enriched gene ontology terms. The higher the significance of the term the more red is the box framing the term. The significant enriched terms are encircled by ellipses.

Cellular-component-PGC KO-<>-PGC CTRL-Log2(2.8284)

Table : Significant GO enrichment terms for Cellular-component-PGC KO-<>-PGC CTRL-Log2(2.8284)

Count Ratio	*p*-val	GO id	GO term	Genes
55/1203	5.5906e□05	GO:0044459	plasma membrane part	Abca1 Abcb1b Aif1l Ank2 Anxa4 Atp1b2 Atp8b1 B2m Car2 Car4 Cd276 Cdhr1 Cltb Dmd Dsp Dtna Efna1 Enpp2 Epb4.1l5 Epha2 Erbb3 F2rl1 Gabbr1 Gjb3 Gng12 Gpc4 H2-Bl Hck Hk1 Itga1 Itgb3 Kcnj3 Kctd15 Kit Laptm5 Lcp1 Lmo7 Lrp2 Ly6a Ly75 Ncam1 Pth1r Rasgrp2 Robo1 S100a10 S100a6 Slc30a1 Slc6a15 Slc6a8 Slc7a3 Slco4a1 Slco4c1 Sorbs1 Spred1 Tek
10/94	0.00011276	GO:0030055	cell-substrate junction	Actn2 Cass4 Dmd Enah Epb4.1l5 Lpp Parvb Sorbs1 Tes Trim25
28/547	0.00057034	GO:0030054	cell junction	Abcb1b Actn2 Ahnak Camk2n1 Cass4 Cldn5 Cpeb1 Dmd Dsp Dtna Enah Epb4.1l5 Fermt3 Gabbr1 Gjb3 Homer2 Jam2 Lcp1 Lmo7 Lpp Parvb Pecam1 Rasgrp2 Rimbp2 Rims1 Sorbs1 Tes Trim25
6/50	0.0011676	GO:0031256	leading edge membrane	Aif1l Epb4.1l5 Gabbr1 Lcp1 Rasgrp2 Robo1
11/147	0.0012404	GO:0005912	adherens junction	Actn2 Cass4 Dsp Enah Epb4.1l5 Lmo7 Lpp Parvb Sorbs1 Tes Trim25
4/22	0.0013777	GO:0032587	ruffle membrane	Aif1l Epb4.1l5 Lcp1 Rasgrp2
36/838	0.0026916	GO:0042995	cell projection	Actn2 Aif1l Calca Camk2n1 Car2 Cdk6 Crmp1 Cyth3 Dner Dock4 Dpysl3 Enah Fermt3 Fez1 Frmd4b Gabbr1 Gli1 Gli2 Itga1 Kif3a Lcp1 Lrp2 Mbp Mtap1b Ncam1 Nefl Pten Ptpn13 Rasgrp2 Rgs19 Robo1 S100a6 Sept4 Spp1 Stmn2 Tek
9/118	0.0028231	GO:0031253	cell projection membrane	Aif1l Atp8b1 Epb4.1l5 Gabbr1 Lcp1 Lrp2 Pth1r Rasgrp2 Robo1
4/28	0.0036285	GO:0005884	actin filament	Afap1 Aif1l Gng12 Lcp1
12/192	0.0038333	GO:0045121	membrane raft	Abca1 Dmd Gabbr1 Hck Hk1 Itga1 Pecam1 Plcd1 Rgs16 Sorbs1 Spred1 Tek
15/281	0.00639	GO:0009986	cell surface	Ceacam1 Dmd Fgfbp1 Fgfr2 Gpr126 H2-Bl Itgb3 Kcnj3 Kit Notch4 Pecam1 Robo1 Slit2 Sulf1 Tek
42/1089	0.0090185	GO:0000267	cell fraction	Abca1 Atp8b1 Camk2n1 Car4 Ccdc88c Cerk Cpeb1 Cyp1b1 Dmd Dpysl3 Dtna Fgf4 Gabbr1 Gch1 Gli2 Gpc4 Id1 Itga1 Itgb3 Jakmip1

				Kif1b Lmna Lrp2 Mapk13 Mep1b Mtap1b Nr0b1 Pea15a Pecam1 Plcd1 Plcd3 Plce1 Prps1 Prps2 Pten Ptgs1 Pygl Rasgrp2 Rgs16 Rgs19 Serpine2 Slc27a2
5/57	0.01175	GO:0001726	ruffle	Cdk6 Cyth3 Frmd4b Lcp1 S100a6
76/2240	0.015546	GO:0005886	plasma membrane	Abca1 Adam23 Aif1l Ank2 Camk2n1 Car4 Cdhr1 Ceacam1 Cldn5 Cpeb1 Cxcr4 Cyth3 Diras2 Dmd Dner Dtna Efna1 Enox1 Epb4.1l5 Epha2 Eras F2rl1 Fez1 Fgfbp1 Gab2 Gabbr1 Gbp2 Gjb3 Gng12 Gpc4 Gpr126 H2-Bl Homer2 Jam2 Lcp1 Lpar4 Lpp Ly6a Mbp Meg3 Mras Mtap1b Ncam1 Notch4 Paqr8 Parvb Pecam1 Plce1 Prkch Ptgs1 Pth1r Ptpn13 Ptpre Rab31 Rasgrp2 Rgs16 Rgs19 Rimbp2 Rims1 S100a6 Sfrp1 Slc30a1 Slc38a4 Slc40a1 Slc5a5 Slc7a3 Slco4c1 Slit2 Sorbs1 Spred1 Tek Tiam1 Trh Trpm1 Tst Ttyh2
36/947	0.018128	GO:0044430	cytoskeletal part	1190002H23Rik Actn2 Actn3 Afap1 Aif1l Arpc1b Aurkc Camk2n1 Cdc14a Cpeb1 Crmp1 Enah Fermt3 Fez1 Gng12 Homer2 Igf2bp2 Jakmip1 Kif1b Kif3a Lcp1 Lmna Mtap1b Myo1f Nefl Pcgf5 Pea15a Psrc1 Rassf5 Sept4 Sfi1 Sorbs1 Spnb3 Tubb2a Tubb2a-ps2 Tubb2b
31/798	0.020459	GO:0005856	cytoskeleton	1190002H23Rik Actn2 Afap1 Aif1l Ank2 Arpc1b Aurkc Calcoco2 Cass4 Cdc14a Coro2b Crmp1 Dmd Dsp Enah Epb4.1l5 Epb4.9 Fez1 Frmd4b Jakmip1 Kif1b Lcp1 Mical1 Mtap1b Myo1f Parvb Psrc1 Ptpn13 Sept4 Sfi1 Sorbs1
10/189	0.023865	GO:0009897	external side of plasma membrane	B2m Cd276 Gpc4 Itga1 Itgb3 Kcnj3 Kit Ly6a Ly75 Ncam1
138/4424	0.024564	GO:0005737	cytoplasm	1190002H23Rik Abhd5 Actn2 Afap1 Aif1l Aifm3 Ak4 Akr1b8 Ank2 Anxa4 Arpc1b Atg16l2 Atp1b2 Aurkc Bcl11a Calca Calcoco2 Car2 Car3 Cass4 Ccbl1 Ccdc88c Ccnd1 Cdc14a Cdk6 Celf4 Cerk Cited2 Ckb Coro2b Cpeb1 Crmp1 Cth Cyth3 Ddit4l Dmd Dnmt3l Dppa3 Dpysl3 Dtna Enah Epas1 Epb4.1l5 Epb4.9 Fez1 Fgfr2 Foxp1 Frmd4b Gab2 Gabbr1 Galk1 Gbp3 Gca Gch1 Gfpt2 Gli1 Gli2 Gpr126 Grb10 Gstm1 Gstm3 Gstt2 H2-Bl Habp4 Hhex Homer2 Hpgd Hspb8 Id1 Igf2bp2 Irak3 Itga1 Jakmip1 Kif1b Kit Lcp1 Lnx1 Lpp Lrp2 Mbd2 Mbnl2 Mbp Mic...
19/448	0.027637	GO:0044463	cell projection part	Aif1l Atp8b1 Calca Cldn5 Cxcr4 Dpysl3 Epb4.1l5 Gabbr1 Kif3a Lcp1 Lrp2 Mbp Mtap1b Nefl Pten Pth1r Rasgrp2 Robo1 Stmn2
32/851	0.029318	GO:0005626	insoluble fraction	Abca1 Atp8b1 Camk2n1 Car4 Ccdc88c Cerk Cpeb1 Cyp1b1 Dmd Dtna Gabbr1 Gli2 Gpc4 Itga1 Itgb3 Jakmip1 Kif1b Lmna Lrp2 Mep1b Nr0b1 Pea15a Plcd1 Plcd3 Plce1 Pten Ptgs1 Rasgrp2 Rgs16 Rgs19 Serpine2 Slc27a2
10/196	0.031058	GO:0016324	apical plasma membrane	Abcb1b Ank2 Anxa4 Atp1b2 Atp8b1 Car4 Erbb3 Lmo7 Lrp2 Tek
26/669	0.032869	GO:0031982	vesicle	Abca1 Acpp Ap1s2 Ap3b2 Ap3m2 Cltb Crispld2 Dpysl3 Gabbr1 Homer2 Itga1 Kif1b Kif3a Kit Lrp2 Pla2g1b Rab6b Rffl Rgs19 Serpine2 Si Slc30a2 Slc40a1 Spp1 Stmn2 Trh
21/517	0.034167	GO:0031988	membrane-bounded vesicle	Abca1 Acpp Crispld2 Dpysl3 Gabbr1 Homer2 Itga1 Kif1b Kif3a Kit Lrp2 Pla2g1b Rab6b Rffl Rgs19 Serpine2 Si Slc30a2 Slc40a1 Spp1 Trh
5/79	0.048171	GO:0044448	cell cortex part	Actn2 Epb4.9 Myo1f Sept4 Spnb3

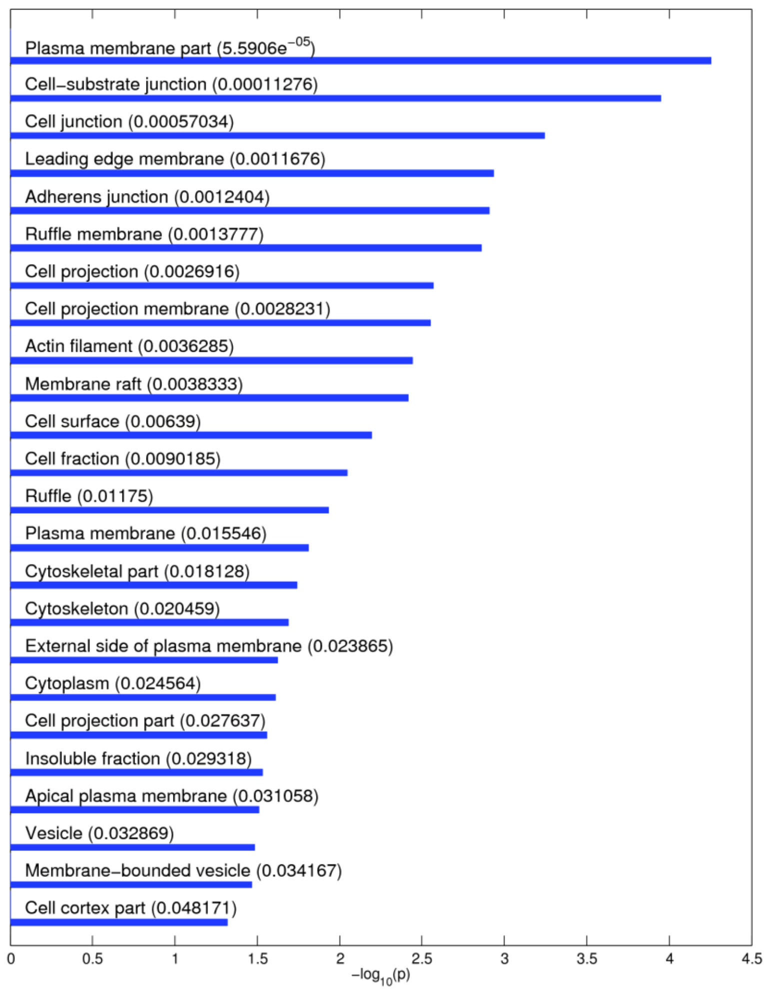

Figure 35: Plot bar of the -log10(p) of the significant enriched terms of Cellular-component-PGC KO-<>-PGC CTRL-Log2(2.8284). The longer the bar, the higher is the statistical significance of the enrichment (in parenthesis are written the p-values)

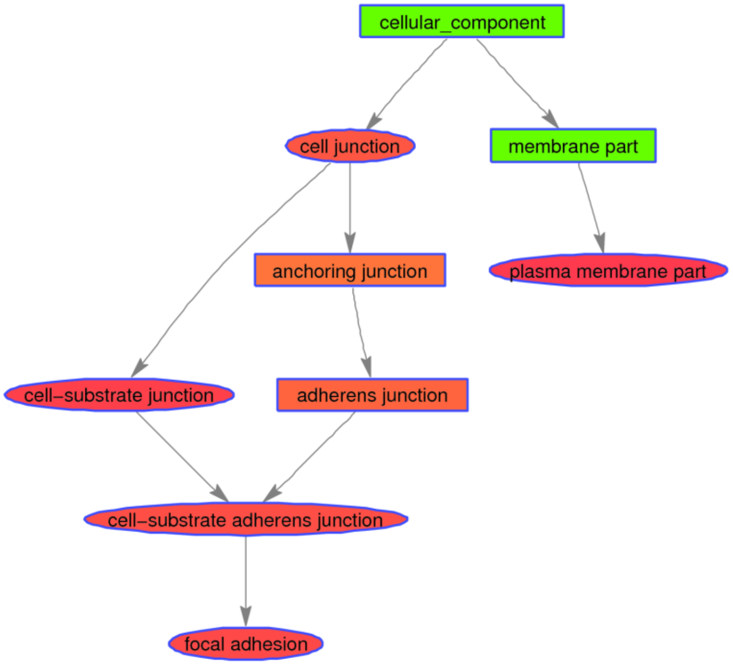

Figure 36: Direct acyclic graph (DAG) associated to the 5 top higher significant enriched gene ontology terms. The higher the significance of the term the more red is the box framing the term. The significant enriched terms are encircled by ellipses.
